# SVD Identifies Transcript Length Distribution Functions from DNA Microarray Data and Reveals Evolutionary Forces Globally Affecting GBM Metabolism

**DOI:** 10.1371/journal.pone.0078913

**Published:** 2013-11-25

**Authors:** Nicolas M. Bertagnolli, Justin A. Drake, Jason M. Tennessen, Orly Alter

**Affiliations:** 1 Scientific Computing and Imaging Institute, University of Utah, Salt Lake City, Utah, United States of America; 2 Department of Bioengineering, University of Utah, Salt Lake City, Utah, United States of America; 3 Department of Human Genetics, University of Utah, Salt Lake City, Utah, United States of America; UCLA-DOE Institute for Genomics and Proteomics, United States of America

## Abstract

To search for evolutionary forces that might act upon transcript length, we use the singular value decomposition (SVD) to identify the length distribution functions of sets and subsets of human and yeast transcripts from profiles of mRNA abundance levels across gel electrophoresis migration distances that were previously measured by DNA microarrays. We show that the SVD identifies the transcript length distribution functions as “asymmetric generalized coherent states” from the DNA microarray data and with no *a-priori* assumptions. Comparing subsets of human and yeast transcripts of the same gene ontology annotations, we find that in both disparate eukaryotes, transcripts involved in protein synthesis or mitochondrial metabolism are significantly shorter than typical, and in particular, significantly shorter than those involved in glucose metabolism. Comparing the subsets of human transcripts that are overexpressed in glioblastoma multiforme (GBM) or normal brain tissue samples from The Cancer Genome Atlas, we find that GBM maintains normal brain overexpression of significantly short transcripts, enriched in transcripts that are involved in protein synthesis or mitochondrial metabolism, but suppresses normal overexpression of significantly longer transcripts, enriched in transcripts that are involved in glucose metabolism and brain activity. These global relations among transcript length, cellular metabolism and tumor development suggest a previously unrecognized physical mode for tumor and normal cells to differentially regulate metabolism in a transcript length-dependent manner. The identified distribution functions support a previous hypothesis from mathematical modeling of evolutionary forces that act upon transcript length in the manner of the restoring force of the harmonic oscillator.

## Introduction

Transcription of messenger RNA (mRNA) associates a cell's genotype with its phenotype in all known organisms. In eukaryotes, unlike prokaryotes, multiple possible mRNAs of, e.g., different lengths, can be and usually are produced, i.e., transcribed and processed in the cell's nucleus and modified in the cytoplasm, which correspond to just a single gene. This diversity of mRNAs has been suggested as a possible origin of the diversity of eukaryotes in general [1] and of neurons in the human brain in particular [2].

Eukaryotic mRNA transcription usually starts with the binding of DNA-dependent RNA polymerase (RNAP) II at one of several possible sites for the 5′ end of the synthesized transcript, at the DNA-encoded promoter region upstream of the 5′ end of a gene. Transcription proceeds when RNAP II escapes the promoter and commits to elongating the mRNA precursor (pre-mRNA) with a sequence of RNA nucleotides complementary to the nucleotides encoded by the template strand, which encodes the DNA sequence complementary to that of the gene. Processing starts soon after RNAP II commits to elongation, with the capping of the pre-mRNA at its 5′ end by a methylated guanosine. Throughout its synthesis, the pre-mRNA is additionally processed via, e.g., alternative splicing that removes one of multiple possible combinations of DNA-encoded regions from the RNA sequence, or alternative editing that effectively, independent of the DNA template, deletes some nucleotides from and inserts some nucleotides into the RNA sequence. Transcription almost always ends with polyadenylation at one of multiple possible sites for the 3′ end of the pre-mRNA, at the DNA-encoded region downstream of the 3′ end of the gene, where a poly(A) tail, of up to, e.g., 250 adenosines in human and 100 adenosines in the yeast *Saccharomyces cerevisiae*, is added to the transcript. The mRNA transcript is then exported from the nucleus to the cytoplasm, where its sequence is translated according to the genetic code into any number of approximately identical sequences of amino acids, i.e., proteins, and where the mRNA transcript may be degraded at any time, with typical mRNA half-lives of, e.g., several hours in human and 10–20 minutes in yeast.

The lengths of the nascent RNA, pre-mRNA and mRNA transcript contribute to the regulation of mRNA transcription and processing. For example, RNAs lesser than five nucleotides (nt) in length form unstable complexes with RNAP II, and therefore, during transcription initiation, RNAP II usually alternates several times between releasing the RNA and reinitiating transcription before a nascent RNA of more than five nt is polymerized [3]. The nascent RNA has to reach a length of approximately 10 nt before RNAP II can dissociate from the transcription factors that bind it to the DNA promoter and commit to traversing the DNA template [4]. The pre-mRNA has to reach a length of 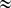
25 nt before the C-terminal end of RNAP II can be hyperphosphorylated to form the elongation complex [5] and the pre-mRNA can be capped at its 5′ end [6].

In another example, only mRNAs that are greater than 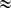
200–300 nt in length are exclusively transported across the nuclear membrane via the mRNA-specific nuclear export pathway [7]. Progressive shortening of intronless mRNAs to less than 200–300 nt in length was found to increase the likelihood of mRNA export via a pathway that is usually reserved for the RNAP II-transcribed uridine-rich small nuclear RNAs (U snRNAs). At lengths 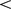
120 nt, mRNAs are exclusively transported via the U snRNA export pathway. It was recently shown that this sorting of RNAs by length is due to the heterotetramer of the heterogeneous nuclear ribonucleoprotein C1/C2 that is essential for export via the mRNA-exclusive pathway, selectively binding to unstructured RNA regions that are 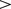
200–300 nt in length [8].

In yet another example, the fruit fly *Drosophila melanogaster* was shown to abort nascent transcripts at each mitosis, and therefore suppress, during early embryonic development, the expression of transcripts that are too long to be completed in a single, rapid embryonic nuclear division cycle, including transcripts that are needed for later developmental stages [9]. In the postembryonic fly, the timing of a gene's activation in response to the steroid hormone ecdysone was shown to be largely determined by the lengths of the gene's mRNA isoforms, where the shorter isoforms are active before the longer ones [10].

We, therefore, propose that evolutionary forces act upon the diversity of eukaryotic mRNA transcript lengths. To search for such evolutionary forces, we use the singular value decomposition (SVD) [11] to identify the length distribution functions of sets and subsets of human and yeast transcripts from profiles of mRNA abundance levels across gel electrophoresis migration distances that were previously measured by DNA microarrays [12], [13]. Comparing subsets of human and yeast transcripts of the same gene ontology (GO) annotations [14], our underlying assumption is that transcripts involved in similar or even conserved pathways in the two organisms may be subject to similar evolutionary forces [15]. Comparing subsets of human transcripts that are overexpressed in either normal brain or glioblastoma multiforme (GBM) tumor tissue samples from The Cancer Genome Atlas [16], [17], our underlying assumption is that similar gene expression in response to the normal brain's transformation to a GBM tumor may be subject to similar evolutionary forces [18].

Note that, in general, while it is possible to estimate some of the statistical moments of a distribution function from data that sample the function, it is not necessarily possible to identify the function from the data [19]. This is because identifying a distribution function is mathematically equivalent to estimating the *infinite* number of moments that are associated with the function. For example, the average and variance, which are defined by the first and second moments, are necessary but not sufficient to identify the function. Therefore, a distribution function for the description of observed diversity is usually derived by assuming an underlying stochastic process, and is tested by its fit, or the fit of its moments, to data. Examples include the Brownian motion [20], the bacterial sensitivity and resistance to viruses [21], and recently, measurements of mRNA expression in single cells [22]–[24].

The SVD of data that sample a distribution function, however, may approximately identify the distribution function from the data and with no *a-priori* assumptions. This is because identifying a distribution function is also equivalent to estimating its eigenfunctions and corresponding eigenvalues. The SVD uncovers in the data unique eigenvectors and corresponding eigenvalues. The *finite* (and, possibly, few) most significant eigenvectors and corresponding eigenvalues – most significant in terms of the fractions of the information that they capture in the data – may approximate the data. If these significant eigenvectors and corresponding eigenvalues fit a series of orthogonal functions and a corresponding series of nonnegative numbers, which are known to be among the eigenfunctions and corresponding eigenvalues, respectively, of a specific distribution function, then this function is identified by the SVD as the distribution function that the data sample.

For example, previously we showed that the few most significant eigenvectors and corresponding eigenvalues, uncovered by the SVD of yeast global transcript length distribution data, fit a series of “asymmetric Hermite functions” and a corresponding geometric series, respectively [25]. From these eigenvectors and eigenvalues it follows that the length distribution function of the global set of yeast transcripts approximately fits an “asymmetric generalized coherent state,” where each transcript's profile fits an “asymmetric Gaussian,” and where the distribution of the peaks of these profiles also fits an asymmetric Gaussian.

We now find that, first, the SVD identifies the length distribution functions of the human and yeast global sets and subsets of transcripts as asymmetric generalized coherent states from the DNA microarray data and with no *a-priori* assumptions. Second, in both human and yeast, transcripts involved in protein synthesis or mitochondrial metabolism are significantly shorter than typical, and in particular, significantly shorter than those involved in glucose metabolism. Third, as a normal tissue is transformed to a tumor tissue, overexpression of significantly short transcripts, enriched in transcripts that are involved in protein synthesis or mitochondrial metabolism, is maintained. However, significantly longer transcripts that are normally overexpressed, enriched in transcripts that are involved in glucose metabolism and brain activity, are suppressed in the tumor.

We propose that it is the GBM tumor's shorter-than-normal brain cell cycle period that limits the production of longer-than-typical transcripts in the GBM tumor cell but not the normal brain cell. The global relations among transcript length, cellular metabolism and tumor development suggest a previously unrecognized physical mode for tumor and normal cells to differentially regulate metabolism in a transcript length-dependent manner. The identified distribution functions support our previous hypothesis from mathematical modeling of evolutionary forces that act upon transcript length in the manner of the restoring force of the harmonic oscillator.

## Methods

### SVD identifies the length distribution functions of human and yeast sets and subsets of transcripts as asymmetric generalized coherent states

Hurowitz *et al* used DNA microarrays to assay the abundance levels of mRNAs from normal human brain tissue in 50 agarose gel slices of two mm each, spanning an electrophoretic migration range of 26–124 mm and the corresponding transcript length range of approximately 6,400–500 nt [12]. Yeast mRNA abundance levels were similarly assayed in 30 gel slices spanning electrophoretic migration of 42–100 mm and transcript lengths of 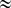
4,500–300 nt [13]. The transcript length distribution data sets we analyze tabulate the mRNA abundance levels of the 4,109 human genes and 3,620 yeast open reading frames (ORFs) with no missing data across the 50 human and 30 yeast DNA microarrays, respectively (Datasets S1 and S2).

**Figure 1:**
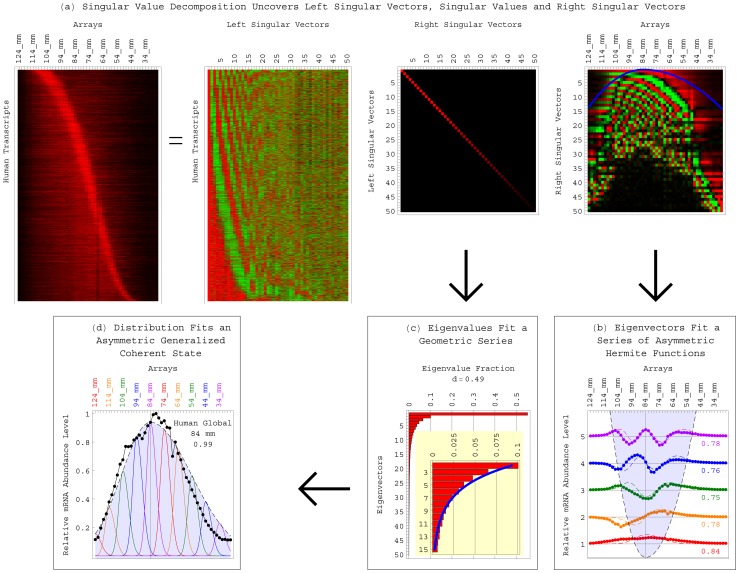
The SVD identifies the length distribution functions of the human and yeast global sets and subsets of transcripts as asymmetric generalized coherent states from the DNA microarray data and with no *a-priori* assumptions. In general, it is not necessarily possible to identify a distribution function from data that sample the function. This is because identifying a distribution function is mathematically equivalent to estimating the *infinite* number of moments that are associated with the function. The SVD of data that sample a distribution function, however, may approximately identify the distribution function from the data and with no *a-priori* assumptions. This is because identifying a distribution function is also equivalent to estimating its eigenfunctions and corresponding eigenvalues. (*a*) The SVD of Equation (1) of the matrix *D* that tabulates the mRNA abundance levels of the human global set of transcripts, in increasing order of the transcript lengths as determined by Hurowitz *et al*, across *X* gel electrophoresis migration distances, uncovers *X* unique left singular vectors, *X* corresponding singular values and *X* corresponding right singular vectors. The orthonormal right singular vectors are also eigenvectors of the matrix 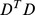, with the eigenvalues proportional to the singular values. The *finite* (and, possibly, few) most significant eigenvectors and corresponding eigenvalues – most significant in terms of the fractions of the information that they capture in the data – may approximate the data. (*b*) The *finite* and few most significant eigenvectors uncovered by the SVD of the human global transcript length distribution data fit a series of orthogonal asymmetric Hermite functions, where the 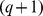
th eigenvector is proportional to the *q*th asymmetric Hermite function of Equations (2) and (3). (*c*) The corresponding eigenvalues and eigenvalue fractions fit a corresponding geometric series. (*d*) The series of asymmetric Hermite functions and the corresponding geometric series are known to be among the eigenfunctions and corresponding eigenvalues, respectively, of the asymmetric generalized coherent state of Equations (4) and (5). Therefore, the asymmetric generalized coherent state, where each transcript's profile fits an asymmetric Gaussian, and where the distribution of the peaks of these profiles also fits an asymmetric Gaussian, is identified by the SVD as the distribution function that the data sample.

Let the matrix *D* tabulate the abundance levels of the set or subset of *P* transcripts across *X* gel slices. The SVD [11],
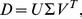
(1)uncovers *X* unique left singular vectors, comprising the columns of the column-wise orthonormal matrix *U*, *X* corresponding singular values, comprising the diagonal of the nonnegative diagonal matrix 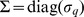, and *X* corresponding right singular vectors, comprising the rows of the orthonormal matrix 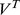 (Figure 1 and Notebook S1). The right singular vectors are also the eigenvectors of the symmetric matrix 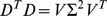, with the corresponding eigenvalues 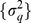. Both the left singular vectors and the right singular vectors, i.e., the eigenvectors, are arranged in decreasing order of the corresponding singular values 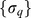, which is also the decreasing order of the eigenvalues 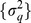 and the eigenvalue fractions, i.e., 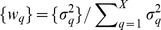. The “normalized Shannon entropy” of *D*, i.e., 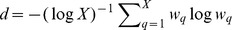, measures the complexity of the data from the distribution of the fractions among the eigenvectors. An entropy of 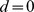 corresponds to an ordered and redundant dataset where just a single eigenvector has a nonzero fraction, such that 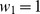 and 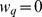 for all 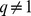. An entropy of 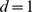 corresponds to a disordered and random dataset where all eigenvectors have identical fractions, such that 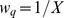 for all 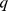.

Consider the *X* unique eigenvectors and corresponding eigenvalues. We find that the most significant eigenvectors fit a series of orthogonal asymmetric Hermite functions, where the 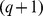
th eigenvector is proportional to the *q*th asymmetric Hermite function.



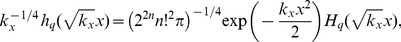
(2)and where 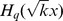 is the *q*th Hermite polynomial. This function generalizes the *q*th eigenfunction of the quantum harmonic oscillator [19], [26] with a “generalized Hooke's constant” 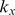 that is asymmetric with respect to the equilibrium 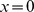,
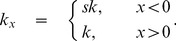
(3)The inflection points of the first through 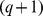
th asymmetric Hermite functions 
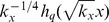
, therefore, sample the “asymmetric parabola” 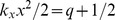 at unit intervals. We also find that the corresponding series of eigenvalues is proportional to the geometric series 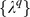.

As we previously showed [25], it follows from these most significant eigenvectors and corresponding eigenvalues that the length distribution function of the set or subset of transcripts is approximately proportional to the asymmetric generalized coherent state 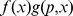, where
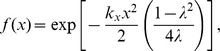


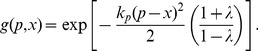
(4)


According to this distribution function [27], [28], the distribution of the peaks of the *P* transcript profiles across the *X* gel slices fits an asymmetric Gaussian 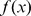 which width is asymmetric with respect to the Gaussian's center at the equilibrium 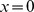, i.e., inversely proportional to 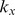. The profile of the 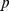
th transcript also fits an asymmetric Gaussian 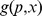 which width is asymmetric with respect to the Gaussian's center at 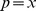 with the same asymmetry *s*, i.e., inversely proportional to the generalized Hooke's constant.
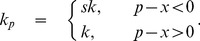
(5)


**Figure 2:**
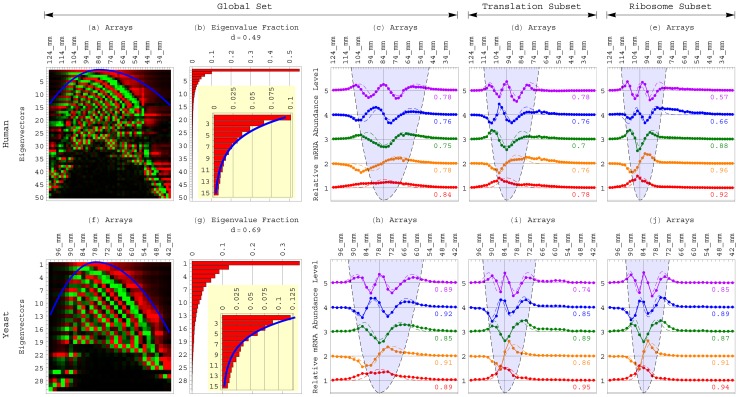
The SVD of the transcript length distribution data of the human and yeast global sets and protein synthesis subsets. (*a*) Raster display of the eigenvectors 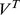 of Equation (1) of the human global set, i.e., 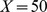 patterns of mRNA abundance level variation across the 50 human DNA microarrays, with overabundance (red), no change in abundance (black) and underabundance (green) around the “ground state” of abundance, which is captured by the first, most significant eigenvector. The inflection points of the 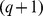
th eigenvector approximately sample the asymmetric parabola 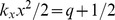 (blue), where 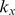 is the generalized Hooke's constant of Equation (3). (*b*) Bar chart of the corresponding eigenvalue fractions 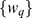, with the normalized Shannon entropy 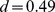. The 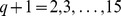 eigenvalues 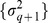 and eigenvalue fractions approximately fit the geometric series 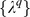 (blue), with 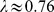. (*c*) Line-joined graphs of the first (red), second (orange), third (green), fourth (blue) and fifth (violet) most significant eigenvectors of the human global set. The 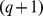
th eigenvector is approximately proportional to the *q*th asymmetric Hermite function 
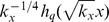
 of Equation (2), where the correlation is in the range of 0.75 to 0.84. The equilibrium 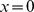 of the asymmetric parabola (dashed and shaded), and therefore also of the corresponding transcript length distribution function, is at the gel migration distance of 84 mm, corresponding to a transcript length of 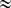
1,700±100 nt. The asymmetry is 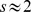. (*d*) Graphs of the first (red) through fifth (violet) eigenvectors of the human translation (GO:0006412) subset. The equilibrium is shifted from that of the human global set to the greater migration distance of 96 mm and lesser transcript length of 1,125±75 nt. The width is lesser than that of the human global set, where the magnitude *k* of the generalized Hooke's constant 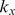 is twice that of the global set, while the asymmetry *s* is similar. (*e*) Eigenvectors of the human ribosome (GO:0005840) subset. The equilibrium is shifted from those of the global set and translation subset to the greater migration distance of 100 mm and lesser transcript length of 975±75 nt. The width is lesser than those of the global set or translation subset, where *k* is three times that of the global set, while *s* is similar. (*f*) Raster display of the 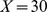 eigenvectors of the yeast global set. (*g*) Bar chart of the corresponding eigenvalue fractions. The 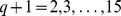 eigenvalues and eigenvalue fractions approximately fit the geometric series 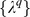 (blue), with 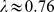 for the yeast global set. (*h*) Line-joined graphs of the first (red) through fifth (violet) eigenvectors of the yeast global set. The 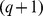
th eigenvector is approximately proportional to the *q*th asymmetric Hermite function, where the correlation is in the range of 0.85 to 0.92. The equilibrium of the transcript length distribution function of the global yeast set is at the gel migration distance of 78 mm and the transcript length of 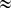
1,025±100 nt. The asymmetry 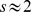 is similar to that of the human global set. (*i*) Eigenvectors of the yeast translation subset. The equilibrium is shifted from that of the yeast global set to the greater migration distance of 84 mm and lesser transcript length of 775±75 nt. The width is lesser than that of the yeast global set, where the magnitude *k* of the generalized Hooke's constant is twice that of the global set, while the asymmetry *s* is similar. (*j*) Eigenvectors of the yeast ribosome subset. The equilibrium is similar to that of the yeast translation subset. The width is lesser than those of the global set or translation subset, where *k* is three times that of the global set, while *s* is similar.

To identify the length distribution functions of the human and yeast global transcript sets, therefore, we use the SVD of Equation (1). Fitting the five most significant eigenvectors of the human and separately the yeast transcript length distribution data sets with the series of asymmetric Hermite functions of Equations (2) and (3) for 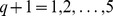, we find that the asymmetry of the generalized coherent state of Equations (4) and (5) is similar for the human and yeast global sets, with 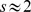 for both organisms (Figure 2). The equilibrium 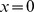 of the human global distribution is at the gel migration distance of 84 mm, which according to Hurowitz *et al* corresponds to a transcript length of approximately 1,700±100 nt. The equilibrium of the yeast global distribution is at the migration distance of 78 mm and a transcript length of 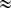
1,025±100 nt. The average correlation between the *q*th asymmetric Hermite function and the 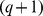
th eigenvector for the five most significant eigenvectors is 0.78 for the human and 0.89 for the yeast. Note that the five most significant eigenvectors capture 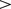
0.8 and 0.7 of the information in the human and yeast data sets, respectively.

Fitting the eigenvalues 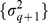 with the geometric series 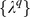 for 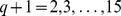, we find that 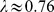 for both the human and yeast. It follows that the ratio of the width of 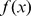, which fits the distribution of the peaks of the transcript profiles, to that of 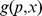, which fits the profile of each transcript, is similar for the human and yeast global sets, with 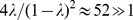 for both organisms. The correlation between the eigenvalues and the geometric series is >0.99 for both organisms.

**Figure 3:**
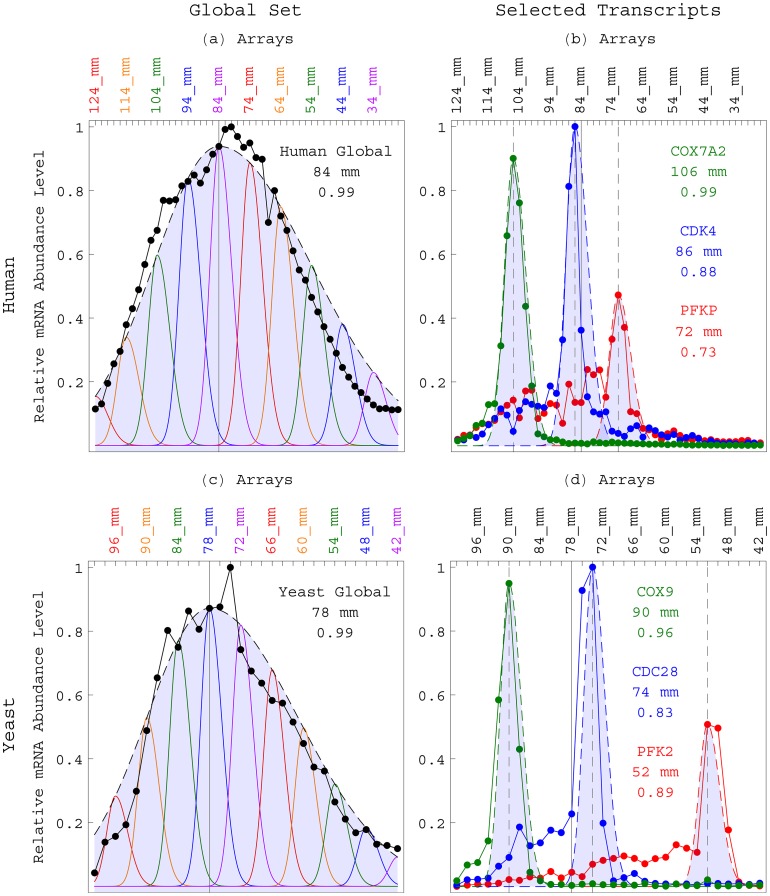
Asymmetric generalized coherent states fit the transcript length distributions of the human and yeast global sets. (*a*) The overall transcript profile of the human global set, i.e., the sum of the profiles of the human transcripts (line-joined), is approximately proportional to the asymmetric generalized coherent state 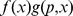 of Equation (4) with 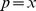, i.e., the asymmetric Gaussian 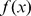 (dashed and shaded), with the equilibrium 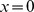 at the migration distance of 84 mm, where the correlation is 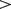
0.99. Graphs of 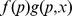 describe the contributions of the subsets of transcript profiles, which peaks 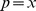 are at the migration distances of 124 (red) through 34 (violet) mm, to the overall transcript profile of the human global set. (*b*) The profiles of the human genes *COX7A2* (green), *CDK4* (blue) and *PFKP* (red) are approximately proportional to the asymmetric Gaussians 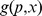 (dashed and shaded) centered at the migration distances of 106, 86 and 72 mm, where the correlations are 0.99, 0.88 and 0.73, respectively. The transcript of *COX7A2*, which is involved in mitochondrial metabolism, is overexpressed in both the normal brain and GBM tumor, at each of the overexpression cutoffs of 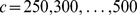. The transcript of *CDK4* is overexpressed in the GBM tumor only. The transcript of *PFKP*, which is involved in glucose metabolism, is overexpressed in the normal brain only. (*c*) The overall transcript profile of the yeast global set (line-joined) is approximately proportional to the asymmetric Gaussian 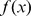 (dashed and shaded), with the equilibrium 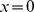 at the migration distance of 78 mm. Graphs of 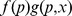 describe the contributions of the subsets of transcript profiles, which peaks 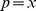 are at the migration distances of 96 (red) through 42 (violet) mm, to the overall transcript profile of the yeast global set. (*d*) The profiles of the yeast genes *COX9* (green), *CDC28* (blue) and *PFK2* (red) are approximately proportional to the asymmetric Gaussian 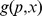 (dashed and shaded) centered at the migration distances of 90, 74 and 52 mm, where the correlations are 0.96, 0.83 and 0.89, respectively. Note that *COX9* is involved in mitochondrial metabolism, whereas *PFK2* is involved in glucose metabolism.

To test the fit of the asymmetric generalized coherent state to the human and yeast transcript length distribution data sets, we calculate the correlation between 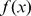 and the overall transcript profile, i.e., the sum of the profiles of the human and separately yeast transcripts (Figure 3). As we previously showed, the overall transcript profile is approximately proportional to the distribution of the peaks of the profiles in the limits of 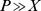 and 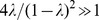. We find that the correlation between the overall transcript profile and 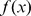 is 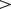
0.99 for both human and yeast.

We also calculate the correlation between 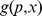 and the transcript profiles of three example pairs of human and yeast genes of the same GO annotations. Consider, for example, the human gene *CDK4* and the yeast gene *CDC28*. These genes encode highly homologous serine/threonine kinases that function as catalytic subunits of cyclin-dependent protein kinase complexes essential to the progression of the cell division cycle in human and yeast, respectively. The peaks 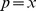 of the transcript profiles of *CDK4* and *CDC28* are near the equilibria of the human and yeast global distributions, at the migration distances of 86 and 74 mm, respectively. This is in agreement with the transcript lengths determined by Hurowitz *et al*, of 1,566 nt for *CDK4* and 1,195 nt for *CDC28*. The human gene *COX7A2* and the yeast gene *COX9* encode isoforms of structural subunits of cytochrome-c oxidase (COX), the terminal enzyme of the mitochondrial respiratory chain. Their transcript profiles are centered at the greater migration distances of 106 and 90 mm, in agreement with the lesser transcript lengths of 735 and 560 nt, respectively. The human *PFKP* and the yeast *PFK2* encode isoforms of phosphofructokinase (PFK), a key enzyme in glycolysis that catalyzes the irreversible conversion of fructose-6-phosphate to fructose-1,6-bisphosphate [29]. Their transcript profiles are centered at the lesser migration distances of 72 and 52 mm, in agreement with the greater transcript lengths of 2,305 and 2,990 nt, respectively. We find that the average correlation between these example gene profiles and 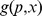 is 0.87 for the human transcripts and 0.89 for the yeast transcripts.

## Results

### Length distributions of subsets of transcripts reveal statistically significant relations, conserved in human and yeast, between a gene's metabolic ontology and its transcript length

To search for evolutionary forces that might act upon transcript length, we use the SVD to similarly identify the length distribution functions of subsets of human and yeast transcripts of the same GO annotations [14]. Our underlying assumption is that transcripts involved in similar or even conserved pathways in the two organisms may be subject to similar evolutionary forces [15]. We find that in both disparate organisms, transcripts involved in protein synthesis or mitochondrial metabolism (including, e.g., the transcripts of the human gene *COX7A2* and the yeast gene *COX9*) are significantly shorter than typical, and in particular, significantly shorter than those involved in glucose metabolism (including, e.g., the transcripts of the human gene *PFKP* and the yeast gene *PFK2*).

For transcripts involved in protein synthesis, we consider the translation (GO:0006412) and ribosome (GO:0005840) subsets. In both human and yeast, we find the equilibria of the global set and the translation and ribosome subset distributions at increasing migration distances, corresponding to decreasing transcript lengths, and with decreasing widths (Figure 2 and Table S1 in Appendix S1). The equilibrium of the human translation subset distribution is shifted six gel slices from that of the human global set to the greater migration distance of 96 mm and lesser transcript length of approximately 1,125±75 nt. The equilibrium of the human ribosome subset is shifted two additional gel slices to the even greater migration distance of 100 mm and lesser transcript length of 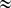
975±75 nt, i.e., 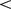
2/3 of the transcript length that corresponds to the equilibrium of the global set. The equilibria of the yeast translation and ribosome subsets overlap, and are shifted three gel slices from that of the global set to the greater migration distance of 84 mm and lesser transcript length of 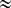
775±75 nt, i.e., 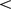
4/5 of the length that corresponds to the equilibrium of the global set. The width of each of these human and yeast transcript subset distributions is lesser than that of the corresponding global set, where the magnitude *k* of the generalized Hooke's constant of each translation and ribosome distribution is twice and three times its magnitude in the corresponding global distribution, respectively, but the asymmetry is similar with 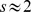 for the translation and ribosome distributions of both organisms.

For transcripts involved in mitochondrial metabolism, we consider the respiratory electron transport chain (ETC) (GO:0022904), mitochondrial respiratory chain complex (MRCC) I (GO:0004129) and COX activity (GO:0005747) subsets (Figure S1 in Appendix S1). The equilibrium of the human respiratory ETC subset is shifted eight gel slices from that of the human global set to the greater migration distance of 100 mm. The equilibria of the human MRCC I and COX activity subsets overlap, and are shifted nine slices to the even greater migration distance of 102 mm and lesser transcript length of 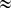
925±75 nt, i.e., 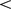
3/5 of the length that corresponds to the equilibrium of the global set. The equilibria of the yeast respiratory ETC and COX activity subsets are shifted two and three gel slices from that of the global set to the greater migration distances of 82 and 84 mm, respectively. The width of each of these human and yeast transcript subset distributions is lesser than that of the corresponding global set.

For transcripts involved in glucose metabolism, we consider the glucose metabolic process (GO:0006006) and glycolysis (GO:0006096) subsets (Figure S2 in Appendix S1). The equilibria of the human glucose metabolic process and glycolysis subsets are shifted four and three gel slices from that of the human global set to the lesser migration distances of 76 and 78 mm and greater transcript lengths of 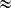
2,175 and 2,050±125 nt, respectively, i.e., 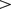
8/5 the length that corresponds to the equilibrium of the human respiratory ETC subset. The equilibria of the yeast glucose metabolic process and glycolysis subsets are both shifted four gel slices from that of the yeast global set to the lesser migration distance of 70 mm and greater transcript length of 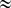
1,425±125, i.e., 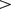
8/5 the length of 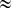
875±75 nt that corresponds to the equilibrium of the yeast respiratory ETC subset. The widths of each of these human and yeast transcript subsets are lesser than that of the corresponding global set.

To assess the significance of the relation between a gene's involvement in protein synthesis or mitochondrial metabolism and a transcript that is shorter than typical, we consider the statistics of the transcript lengths of a subset of *M* genes that is selected from a set of *N* genes. The average and variance of the length of a gene in a set of 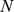 genes, of lengths 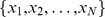, are
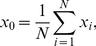
(6)

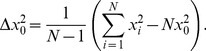
(7)


There are 
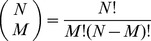
 possible subsets of 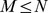 genes in the set. Let the difference between the average transcript length of the *m*th subset of *M* genes, i.e., the average of 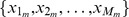, and that of the set be the statistic.
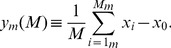
(8)


Since each gene belongs to 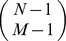 of these subsets, averaging the statistic 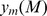 over all possible subsets gives
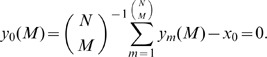
(9)


Similarly, each pair of genes belongs to 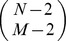 of these subsets, and therefore, the variance of the statistic is
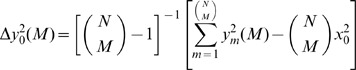


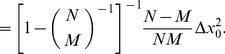
(10)


From Cantelli's inequality, the *P*-value, i.e., the upper bound to the probability that a subset is randomly selected from the set of genes, such that the difference between the average transcript length of this subset and that of the set is 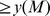 of Equation (8) is
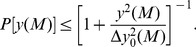
(11)


Note that the *P*-value of Equation (11) depends only on the observed statistic 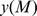 of Equation (8), its average 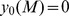 of Equation (9) and its variance 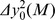 of Equation (10). Therefore, while this *P*-value depends on the first and second moments of the statistic, it is independent of the higher moments of the statistic. We find that for both human and yeast, the *P*-value is 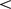
0.05 for the observed difference in the average transcript length of either the translation or ribosome subsets and that of the corresponding global set (Table 1). Similarly, the *P*-value is 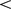
0.05 for the observed difference in the average transcript length of either one of the human subsets of respiratory ETC, MRCC I or COX activity and that of the human global set.

**Table 1:** Human and yeast subsets of average transcript lengths significantly lesser than that of the corresponding global set.

			Human			Yeast		
Transcript Subset			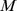	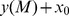	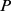 -value	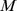	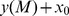	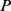 -value
Gene Ontology	Translation		178	2,096	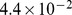	319	1,271	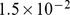
	Ribosome		78	1,582	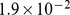	274	1,135	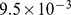
	Respiratory ETC		55	1,460	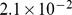			
	MRCC I		25	1,153	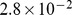			
	COX Activity		14	1,108	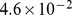			
Normal 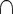 Tumor Overexpression	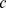	250	200	1,723	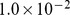			
		300	239	1,779	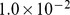			
		350	279	1,823	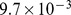			
		400	326	1,833	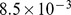			
		450	371	1,860	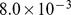			
		500	412	1,917	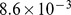			

The *P*-value of Equation (11) is calculated for the average transcript length 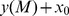 in nucleotides of each human or yeast subset of 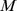 genes relative to the average transcript lengths of 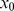 = 2,480 and 1,621 nt of the human and yeast global sets of *N* = 4,109 and 3,620 transcripts, respectively. The subsets of human transcripts that are most abundant in both the normal brain and GBM tumor are considered at each of the overexpression cutoffs of 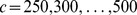.

To assess the significance of the relation between a gene's involvement in glucose metabolism and a transcript that is longer than typical for a gene that is involved in mitochondrial metabolism, we consider the statistics of the transcript lengths of two, possibly overlapping subsets of genes, each independently selected from the union of the two subsets of *N* genes. Let the difference between the average length of the genes in the *l*th subset of *L* genes and the independent *m*th subset of *M* genes be the statistic. From Equation (8), this statistic equals 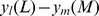. From Equation (9), the average of the statistic is 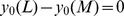. From the independence of the two subsets, the variance of the statistic is the sum of the variances 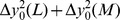, where 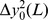 and 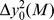 are defined in Equation (10). Therefore, from Cantelli's inequality, the *P*-value that two subsets are randomly and independently selected from the union of the two subsets, such that the difference between the average transcript lengths of these subsets is 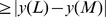 is
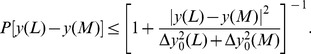
(12)


We find that for both human and yeast, the *P*-value is 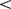
0.05 for the observed difference in the average transcript length of either the glucose metabolic process or glycolysis subsets and that of the corresponding respiratory ETC set (Table 2).

**Table 2:** Human and yeast subsets of average transcript lengths significantly greater than that of the corresponding respiratory electron transport chain (ETC) subset.

			Human			Yeast		
Transcript Subset			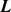	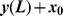	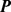 -value	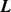	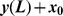	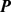 -value
Gene Ontology	Glucose Metabolic Process		100	2,399	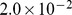	66	1,686	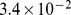
	Glycolysis		29	2,428	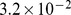	23	1,695	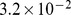
	Neuron Projection		259	2,666	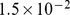			
	Synaptic Transmission		238	2,667	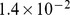			
Tumor\Normal Overexpression	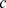	250	135	2,001	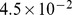			
		300	157	2,051	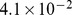			
		350	186	2,194	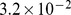			
		400	217	2,310	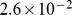			
		450	235	2,386	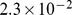			
		500	257	2,401	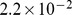			
Normal\Tumor Overexpression		250	102	2,599	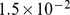			
		300	121	2,683	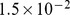			
		350	141	2,586	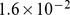			
		400	145	2,620	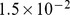			
		450	167	2,631	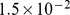			
		500	180	2,603	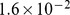			

The *P*-value of Equation (12) is calculated for the average transcript length 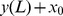 in nucleotides of each human or yeast subset of 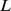 genes relative to the average transcript lengths of 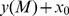 = 1,460 and 995 nt of the human and yeast respiratory ETC subsets of 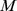 = 55 and 22 transcripts, respectively. The subsets of human transcripts that are most abundant in either the GBM tumor only or the normal brain only are considered at each of the overexpression cutoffs of 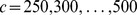.

### Human GBM tumors maintain normal brain overexpression of short transcripts, involved in protein synthesis and mitochondrial metabolism, but suppress longer, normally overexpressed transcripts, involved in glucose metabolism and brain activity

To search for evolutionary forces that might act upon transcript length, we also use the SVD to identify the length distribution functions of subsets of human transcripts that are overexpressed in either normal brain or GBM tumor tissue samples from TCGA [16], [17]. Our underlying assumption is that similar gene expression in response to the normal brain's transformation to a GBM tumor may be subject to similar evolutionary forces [18]. We find that as a normal tissue is transformed to a tumor tissue, overexpression of significantly short transcripts, enriched in transcripts that are involved in protein synthesis or mitochondrial metabolism (including, e.g., the transcript of the human gene *COX7A2*), is maintained. However, significantly longer transcripts that are normally overexpressed, enriched in transcripts that are involved in glucose metabolism (including, e.g., the transcript of the human gene *PFKP*) and brain activity, are suppressed in the tumor.

TCGA used DNA microarrays to assay the abundance levels of mRNAs from ten normal brain tissue samples and 529 GBM tumor samples. The normal brain and GBM tumor gene expression data sets we analyze tabulate the mRNA abundance levels of the 11,631 human genes with at least one start and one end coordinate in the National Center for Biotechnology Information (NCBI) human genome sequence posted at the University of California at Santa Cruz (UCSC) human genome browser [30], [31]. A gene is annotated as overexpressed in either the normal brain or the GBM tumor if it is in the group of 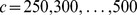 most expressed among the 11,631 genes in at least 20% of the normal or tumor samples, respectively (Dataset S3). A transcript is similarly annotated if it is in the group of 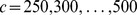 most abundant among the 4,109 transcripts listed in the human transcript length distribution data set in at least 20% of the normal or tumor samples, respectively.

**Figure 4:**
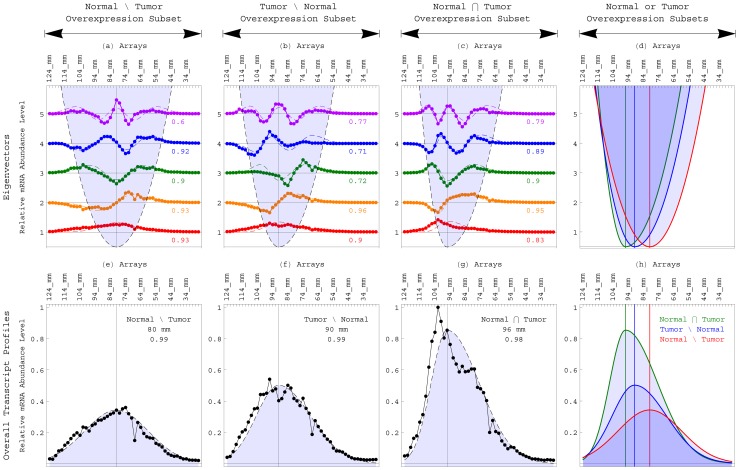
Eigenvectors and overall transcript profiles of the length distribution data of the subsets of human transcripts overexpressed in either the normal brain only, the GBM tumor only or both. (*a*) Line-joined graphs of the first (red), second (orange), third (green), fourth (blue) and fifth (violet) most significant eigenvectors of the subset of human transcripts that are most abundant in the normal brain but not the GBM tumor (including, e.g., *PFKP*), at the overexpression cutoff of 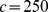. The 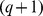
th eigenvector is approximately proportional to the *q*th asymmetric Hermite function, where the correlation is in the range of 0.6 to 0.93. The inflection points of the 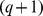
th eigenvector approximately sample the asymmetric parabola 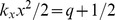 (dashed and shaded). The equilibrium 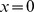 of the asymmetric parabola, and therefore also of the corresponding transcript length distribution function, is shifted from that of the human global set to the lesser migration distance of 80 mm and greater transcript length of 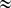
1,875±100 nt. (*b*) Eigenvectors of the subset of transcripts that are most abundant in the GBM tumor but not the normal brain (including, e.g., *CDK4*), at the cutoff of 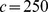. The equilibrium is shifted from those of the normal brain only subset and global set to the greater migration distance of 90 mm and lesser transcript length of 1,375±100 nt. The width of the corresponding length distribution function of the tumor only subset is lesser than that of the normal only subset, where the asymmetry 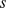 of the generalized Hooke's constant 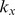 of the GBM tumor only subset is twice that in the normal brain only subset, while the magnitude *k* is similar. (*c*) Eigenvectors of the subset of transcripts that are most abundant in both the normal and tumor (including, e.g., *COX7A2*), at the cutoff of 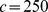. The equilibrium is shifted to the greater migration distance of 96 mm and lesser transcript length of 1,125±75 nt. The width is lesser than those of the normal only subset as well as the tumor only subset, where the asymmetry is four times that in the normal only subset, while the magnitude is similar. (*d*) The asymmetric parabolas that fit the inflection points of the eigenvectors of the length distribution data of the subsets of human transcripts overexpressed in either the normal only (red and shaded), the tumor only (blue and shaded) or both (green and shaded). The equilibria of these parabolas are at increasing migration distances, corresponding to decreasing transcript lengths, and with decreasing widths. (*e*) The overall transcript profile of the subset of human transcripts that are most abundant in the normal brain only, i.e., the sum of the profiles of these transcripts (line-joined), is approximately proportional to the asymmetric Gaussian 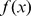 (dashed and shaded), with the equilibrium 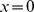 at the migration distance of 80 mm, where the correlation is >0.99. (*f*) The overall profile of the subset of human transcripts that are most abundant in the tumor only (line-joined) is approximately proportional to the asymmetric Gaussian 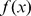 (dashed and shaded), with the equilibrium at 90 mm. (*g*) The overall profile of the subset of human transcripts that are most abundant in both the normal and tumor (line-joined) is approximately proportional to the asymmetric Gaussian 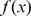 (dashed and shaded), with the equilibrium at 96 mm. (*h*) The asymmetric Gaussians that fit the overall transcript profiles of the length distribution data of the subsets of human transcripts overexpressed in either the normal only (red and shaded), the tumor only (blue and shaded) or both (green and shaded). The equilibria of these Gaussians are at increasing migration distances, corresponding to decreasing transcript lengths.

We find the equilibria of the three mutually exclusive subsets of transcripts that at 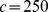 are overexpressed in either the normal brain only, the GBM tumor only or both, at the increasing migration distances of 80, 90 and 96 mm, corresponding to the decreasing transcript lengths of 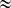
1,875 and 1,375±100 and 1,125±75 nt (Figure 4 and Table S1 in Appendix S1). The transcript length that corresponds to the equilibrium of the subset that is overexpressed in both the normal and tumor is 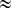
2/3 of the length that corresponds to the equilibrium of the human global set, and 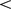
2/3 that of the subset that is overexpressed in the normal only. The lengths that correspond to the equilibria of the subsets that are overexpressed in the tumor only and the normal only are 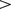
4/3 and 5/3 that of the human respiratory ETC subset, respectively.

**Figure 5:**
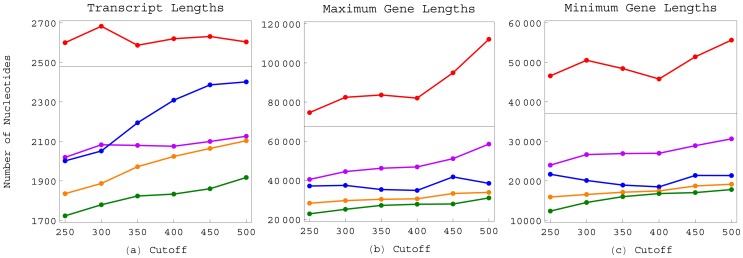
Average transcript and gene lengths of the human subsets overexpressed in the normal brain or the GBM tumor. (*a*) Average transcript lengths of the human subsets that are overexpressed in the normal brain only (red), the normal brain overall (violet), the GBM tumor only (blue), the GBM tumor overall (orange) or both the normal brain and GBM tumor (green), at each of the overexpression cutoffs of 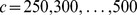, relative to the average transcript length of the global set of 4,109 transcripts (black). (*b*) Average maximum gene lengths of the human subsets that are overexpressed in the normal brain or the GBM tumor at each of the cutoffs, relative to the average maximum gene length of the global set of 11,631 genes. (*c*) Average minimum gene lengths of the human subsets relative to that of the global set.

We also find that at each of the overexpression cutoffs of 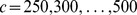, the average length of the subset of transcripts that are overexpressed in the normal brain only is greater than that of the transcripts that are overexpressed in the tumor only (Figure 5). The average length of the transcripts that are overexpressed in the tumor only (including, e.g., the transcript of the human gene *CDK4*), is greater than that of the transcripts that are overexpressed in both the normal and tumor. Note also that the average length of the subset of transcripts that are overexpressed in the normal brain only but not in the GBM tumor is consistently greater than that of the global set of transcripts, even though the average length of the subset of transcripts that are overexpressed in the normal brain overall, regardless of whether they are also overexpressed in the GBM tumor, is consistently lesser than that of the global set of transcripts.

Similarly, at each of the cutoffs, the average maximum and, separately, minimum lengths of the subset of genes – among the 11,631 genes listed in the human gene length distribution data set – that are overexpressed in the normal brain only are greater than those of the genes that are overexpressed in the tumor only. The average maximum and, separately, minimum lengths of the genes that are overexpressed in the tumor only are greater than those of the genes that are overexpressed in both the normal and tumor. The average maximum and minimum lengths of the genes that are overexpressed in the normal brain only but not in the GBM tumor is consistently greater than that of the global set of 11,631 genes, even though the average length of genes that are overexpressed in the normal brain overall, regardless of whether they are also overexpressed in the GBM tumor, is consistently lesser than that of the global set of genes.

**Figure 6:**
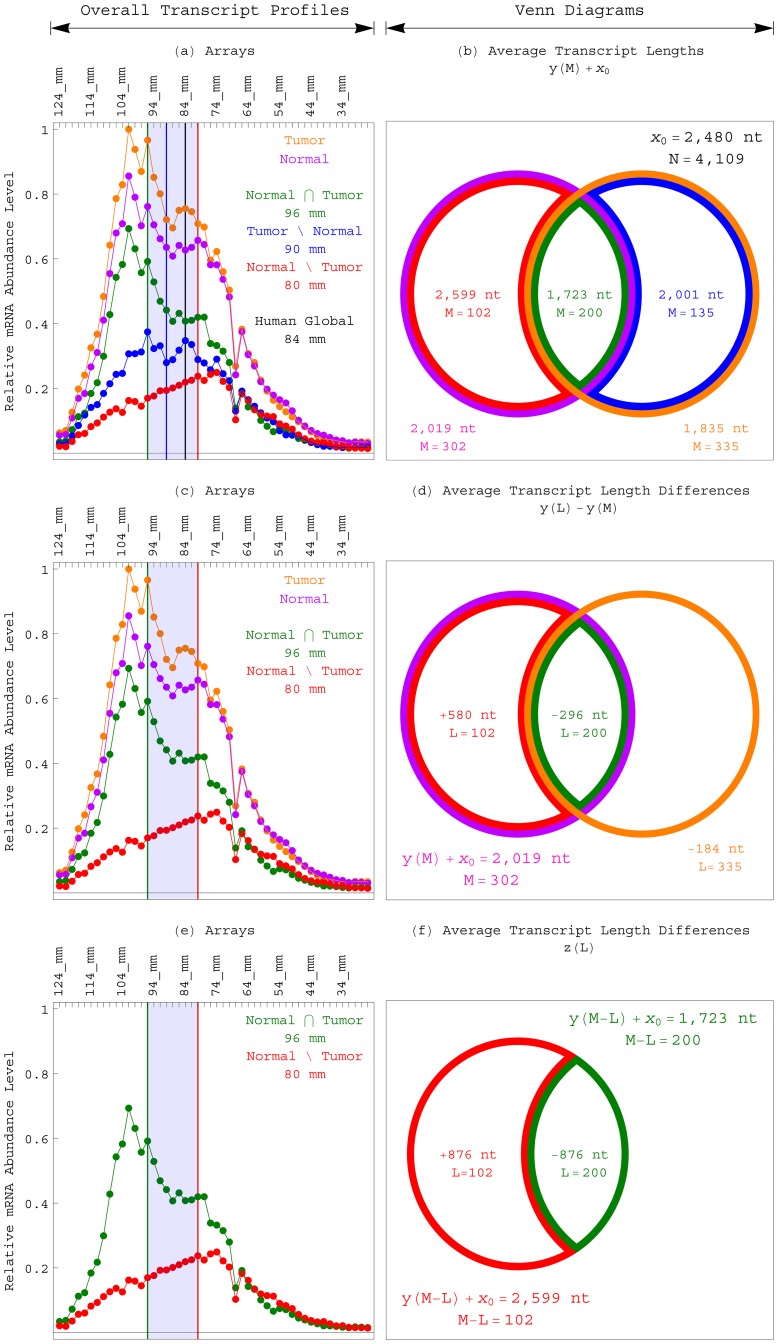
Overall transcript profiles and Venn diagrams of the subsets of human transcripts overexpressed in the normal brain or the GBM tumor. (*a*) The overall transcript profiles of the subsets of human transcripts that are most abundant in the normal brain only (red), the normal brain overall (violet), the GBM tumor only (blue), the GBM tumor overall (orange) or both the normal brain and GBM tumor (green). The equilibria of the profiles of the normal only subset, the human global set, the tumor only subset and the subset of transcripts that are overexpressed in both the normal and tumor are at the increasing migration distances of 80 (red), 84 (black), 90 (blue) and 96 (green) mm, spanning a difference of 16 mm of gel migration distance (shaded), and corresponding to decreasing transcript lengths. (*b*) The average transcript lengths 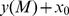 of Equation (8) of the subsets of *M* transcripts each that are most abundant in the normal only (red), the normal overall (violet), the tumor only (blue), the tumor overall (orange) or both the normal and tumor (green), relative to the average transcript length 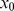 of Equation (6) of the human global set of *N* transcripts, at the overexpression cutoff of 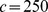. The relation between a gene's overexpression in either the normal overall, the tumor only, the tumor overall or both the normal and tumor and a transcript that is shorter than typical is statistically significant, with the *P*-value of Equation (11) <0.05 for the observed differences in the average transcript lengths of these subsets and that of the human global set (Table 1). (*c*) The overall transcript profiles of the subsets of human transcripts that are most abundant in the normal brain only (red), the normal brain overall (violet), the GBM tumor overall (orange) or both the normal brain and GBM tumor (green). (*d*) The average transcript length differences 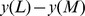 of the subsets of *L* transcripts each that are most abundant in the normal only (red), the tumor overall (orange) or both the normal and tumor (green), relative to the average transcript length 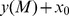 of the normal overall subset of *M* transcripts, at the overexpression cutoff of 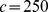. The relations between a gene's overexpression in the tumor overall or in both the normal and tumor and a transcript that is shorter than typical for a gene that is overexpressed in the normal overall are statistically significant, with the *P*-value of Equation (12) <0.05 (Table 2). Similarly, the relation between a gene's overexpression in the normal only and a transcript that is longer than typical for a gene that is overexpressed in the normal overall is statistically significant. (*e*) The overall transcript profiles of the subsets of human transcripts that are most abundant in the normal brain but not the GBM tumor (red) or in both the normal brain and GBM tumor (green). (*f*) The average transcript length differences 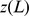 of Equation (13) of the subsets of *L* transcripts that are most abundant in the normal only (red) or in both the normal and tumor (green), relative to the average transcript length 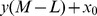 of the subsets of transcripts that are most abundant in both the normal and tumor (green) or in the normal only (red), respectively, at the overexpression cutoff of 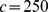. The relation between a gene's overexpression in the normal brain but not the GBM tumor and a transcript that is longer than typical for a gene that is overexpressed in both the normal brain and GBM tumor is statistically significant, with the *P*-value of Equation (15) <0.05.

The relation between a gene's overexpression in both the normal brain and GBM tumor and a transcript that is shorter than typical is statistically significant, with the *P*-value of Equation (11) <0.05 for the observed difference in the average transcript length of the normal and tumor subset and that of the human global set, at each of the overexpression cutoffs of 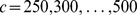 (Figure 6 and Table 1). The relations between a gene's overexpression in the GBM tumor only or the normal brain only and a transcript that is longer than typical for a gene that is involved in mitochondrial metabolism are also statistically significant, with the *P*-value of Equation (12) <0.05 for the observed differences in the average transcript length of either the tumor only subset or the normal only subset and that of the human respiratory ETC subset, at each of the cutoffs (Table 2).

To assess the significance of the relation between a gene's overexpression in the normal brain only and a transcript that is longer than typical for a gene that is overexpressed in both the normal brain and GBM tumor, we consider the statistics of the transcript lengths of two mutually exclusive subsets of genes. Consider the 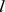
th subset of 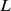 genes selected from a subset of 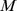 genes, and its complement, the subset of the 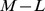 remaining genes. Let the difference between the average transcript length of the 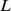 genes and that of the 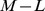 genes, i.e.,
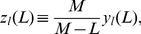
(13)be the statistic. From Equation (9), the average of the statistic is 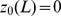. From Equation (10), the variance is
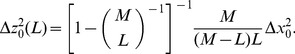
(14)


Therefore, from Cantelli's inequality, the *P*-value that a subset is randomly divided into two subsets, such that the difference between the average transcript lengths of these two mutually exclusive subsets is 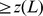 is
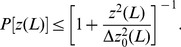
(15)


We find that the *P*-value of Equation (15) is <0.05 for the observed difference in the average transcript length of the normal only subset and that of the normal and tumor subset, at each of the cutoffs.

To examine the relation between a gene's ontology and its overexpression in either the normal brain only, the GBM tumor only or both, we assess the enrichment of these subsets in transcripts of genes that are associated with any one of the multiple GO annotations [32]. The *P*-value of a given enrichment is calculated assuming hypergeometric probability distribution of the 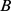 annotations among the 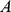 genes in the global set, and of the subset of 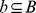 annotations among the subset of 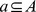 genes, 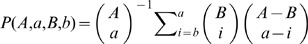 [33].

We find that the subset of transcripts that are overexpressed in both the normal and tumor, which is of the least average transcript length among the three mutually exclusive subsets, is significantly enriched in transcripts that are involved in protein synthesis and mitochondrial metabolism, at each of the cutoffs of 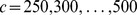 (Table 3 and Table S2 in Appendix S1). The subset that is overexpressed in the normal brain overall, i.e., the union of the mutually exclusive normal only subset and normal and tumor subset, is enriched in transcripts that are involved in glucose metabolism. The normal only subset, which is of the greatest average transcript length among the three mutually exclusive subsets, is enriched in brain activity transcripts, e.g., transcripts involved in neuron projection (GO:0043005) or synaptic transmission (GO:0007268). The SVD identifies the equilibria of the neuron projection and synaptic transmission subsets of human transcripts at the migration distances of 78 and 80 mm and the transcript lengths of 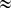
2,050 and 1,875±100 nt, respectively, i.e., >9/5 the length that corresponds to the equilibrium of the human respiratory ETC subset (Figure S3 in Appendix S1). This relation between a gene's involvement in brain activity and a transcript that is longer than typical for a gene that is involved in mitochondrial metabolism is statistically significant, with the *P*-value of Equation (13) <0.05 for the observed differences in the average transcript length of either the neuron projection or the synaptic transmission subset and that of the human respiratory ETC subset, at each of the cutoffs.

**Table 3:** Typical gene ontology (GO) annotations significantly enriching the human subsets of transcripts and genes overexpressed in both the GBM tumor and normal brain, the normal brain overall or the normal brain only.

Overexpression		Global Transcript Set				Global Gene Set			
Subset	Gene Ontology	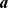	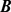	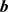	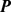 -value	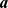	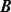	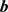	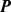 -value
Normal 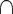 Tumor	Translation	200	178	36	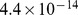	204	380	64	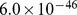
	Ribosome		78	28	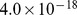		155	52	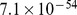
	Respiratory ETC		55	21	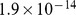		89	22	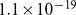
	MRCC I		25	9	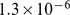		34	6	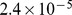
	COX Activity		14	9	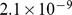		20	8	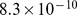
Normal	Glucose Metabolic Process	302	100	17	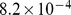	309	187	14	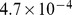
	Glycolysis		29	9	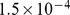		59	6	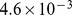
Normal\Tumor	Neuron Projection	102	259	22	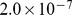	105	534	24	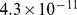
	Synaptic Transmission		238	19	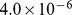		535	26	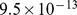

The *P*-value of a given enrichment is calculated assuming hypergeometric probability distribution of the 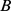 annotations among the 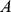 transcripts or genes in the global set, and of the subset of 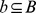 annotations among the subset of 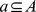 transcripts or genes, 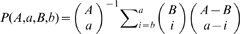. These enrichments of the subsets at the overexpression cutoff of 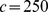 are consistent with the enrichments of the corresponding subsets at the overexpression cutoffs of 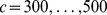 (Table S2 in Appendix S1). None of the multiple GO annotations consistently enrich the human subsets of transcripts and genes that are overexpressed in the GBM tumor only. None of the multiple GO annotations consistently enrich the human subsets of transcripts and genes that are overexpressed in the GBM tumor overall beyond those that enrich the subsets that are overexpressed in both the GBM tumor and normal brain.

Notably, we do not observe any significant enrichments in GO annotations among the subsets of transcripts that are overexpressed in the GBM tumor only that are consistent across the cutoffs. In addition, any significant enrichments in GO annotations among the subsets of transcripts that are overexpressed in the GBM tumor overall that are consistent across the cutoffs, are also observed for the subsets of transcripts that are overexpressed in both the normal brain and GBM tumor.

We similarly find that the subset of genes – among the 11,631 genes that are listed in the human gene length distribution data set – that are overexpressed in both the normal and tumor, is significantly enriched in genes that are involved in protein synthesis and mitochondrial metabolism, at each of the cutoffs of 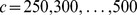 (Table 3 and Table S2 in Appendix S1). The subset of genes that are overexpressed in the normal brain overall is enriched in genes that are involved in glucose metabolism. The normal only subset is enriched in brain activity genes. Examining the relations between a gene's maximum and minimum lengths and its metabolic ontology, we also find that genes, in addition to transcripts, that are involved in protein synthesis and mitochondrial metabolism are significantly shorter than those involved in glucose metabolism and brain activity (Tables S3, S4 and S5 in Appendix S1).

## Discussion

### GBM tumor-exclusive suppression of longer-than-typical transcripts might be due to shorter-than-normal brain cell cycle periods

Our search for evolutionary forces that might act upon transcript length revealed previously unrecognized global relations among transcript length, cellular metabolism and tumor development.

First, we found that human genes that are overexpressed in the GBM tumor but not in the normal brain are of significantly lesser transcript length as well as gene length – as measured in the normal human brain and genome, respectively – than genes that are overexpressed in the normal brain only. No significant enrichments in GO annotations among those genes that are overexpressed in the GBM tumor only are observed. This suggests that genes are globally selected for GBM tumor-exclusive overexpression based upon their normal transcript and gene lengths, beyond the biological processes, molecular functions or cellular components that are associated with the genes. This global relation is complementary to, but different from the observation that overexpression of shorter-than-normal splice variants of several essential genes may play a role in the pathogenesis of cancers. For example, the ribosomal protein S6 kinase 1 is essential to the progression of the G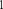 phase of the cell cycle. It was recently shown that overexpression of short mRNA isoforms of the gene that encodes this serine/threonine kinase induces transformation of human breast epithelial cells, whereas the full-length transcript, which is expressed in normal cells, has a tumor-suppressor activity [34]. Similarly, the global relation is complementary to, but different from the observation that mechanisms for alternative splicing in the absence of genomic mutations exist, which lead to cancer-specific overexpression of shorter-than-normal splice variants of several genes at a time. For example, alternative cleavage and polyadenylation can activate oncogenes in cancer cells by shortening the untranslated regions (UTRs) at the 3′ ends of their mRNA transcripts [35]. It was recently shown that the gene poly(A)-binding protein nuclear 1 is involved in suppressing such alternative cleavage and polyadenylation [36]. Such observations suggest that by taking a gene's transcript length to be its measured length in the normal brain, we may be overestimating the lengths of the transcripts that are overexpressed in the GBM tumor, and underestimating the significance of the global relation between a gene's GBM tumor-exclusive overexpression and a transcript that is shorter than typical in the normal brain.

That genes are globally selected for GBM tumor-exclusive overexpression based upon their shorter-than-typical normal brain transcript and gene lengths, might be explained by tumor-exclusive abortion of nascent transcripts of longer-than-typical genes. While a lack of energy might limit a cell's completion of long transcripts, it is not likely that the proliferating GBM tumor cells lack energy for transcription [18].

DNA damage, when it is accompanied by p53-dependent apoptosis, might also limit a cell's production of longer-than-typical genes. In cells exposed to DNA damaging agents, DNA lesions are more likely to affect longer rather than shorter genes [37]. Such lesions block transcription and, via persistent blockage of transcription, trigger p53-dependent apoptosis [38]. It was shown that in response to increasing levels of ultraviolet light, and therefore also increasing levels of DNA damage, human colon carcinoma cells express decreasing numbers of p53-induced genes of decreasing gene lengths [39]. However, when in response to DNA damage a cell cycle checkpoint is activated, arresting a cell's progression through the cell cycle to provide time for DNA repair, the transcripts that are overexpressed might be significantly longer than those that are underexpressed. For example, using the SVD to identify the length distribution functions of the two mutually exclusive subsets of yeast transcripts that were detected as either overexpressed or underexpressed in response to the DNA damaging agent methyl methanesulfonate (MMS) [40], we find the equilibria of these subsets at the migration distances of 74 and 80 mm and the transcript lengths of 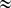
1,250 and 950±100 nt, i.e., longer and shorter, respectively, than the length of 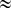
1,025±100 nt that corresponds to the equilibrium of the yeast global set (Figure S4 in Appendix S1). This relation between a gene's overexpression and a transcript that is longer than typical for a gene that is underexpressed in response to MMS is statistically significant, with the *P*-value of Equation (15) <0.05. Nonetheless, GBM tumor cells do not necessarily exhibit either apoptosis or cell cycle arrest [18]. Note that we did not observe any significant enrichments in GO annotations among the transcripts that are overexpressed in the GBM tumor only, and specifically we did not observe any enrichments in GO annotations that relate to either apoptosis or cell cycle arrest. This is not surprising, since the tumor's development and progression require the tumor cells to suppress programmed cell death and deregulate proliferation.

A proliferating GBM tumor's cell cycle period, however, is necessarily shorter than that of the mostly non-proliferating normal brain. The developing fruit fly is known to regulate the expression of several essential genes in a manner that depends, at least in part, upon the time that is required for the transcription of these genes and, therefore, also upon the transcript lengths of these genes. For example, the fruit fly was shown to abort nascent transcripts at each mitosis, and therefore suppress, during early embryonic development, the expression of transcripts that are too long to be completed in a single, rapid embryonic nuclear division cycle, including transcripts that are needed for later developmental stages [9]. In the postembryonic fly, the timing of a gene's activation in response to the steroid hormone ecdysone was shown to be largely determined by the lengths of the gene's mRNA isoforms, where the shorter isoforms are active before the longer ones [10].

We, therefore, propose that it is the GBM tumor's shorter-than-normal brain cell cycle period that limits the production of longer-than-typical transcripts in the GBM tumor cell but not the normal brain cell.

### A previously unrecognized mode for the GBM tumor and normal brain to differentially regulate metabolism in a transcript length-dependent manner

Second, we found that the GBM tumor maintains normal brain overexpression of transcripts that are significantly shorter than typical, enriched in transcripts that are involved in protein synthesis and mitochondrial metabolism, but suppresses normal overexpression of significantly longer transcripts, enriched in transcripts that are involved in glucose metabolism and brain activity. That both the GBM tumor and normal brain overexpress transcripts that are involved in protein synthesis is not surprising. Protein synthesis and, therefore, also ribosomal gene expression are required for the tumor's growth and proliferation [41]. For example, it was shown that among the National Cancer Institute's 60 (NCI60) cancer cell lines, levels of ribosomal gene expression correlate with a cell's doubling time, linking the rates of protein synthesis in the NCI60 cells with their rates of growth and proliferation [42]. Although mostly non-proliferating, the normal brain also requires protein synthesis for its functions, from a neuronal cell's signaling [43] to the amygdala's memory processing [44], and multiple brain disorders have been linked with ribosome dysfunction. It is also not surprising that the GBM tumor suppresses normal expression of genes involved in brain activities, such as neuron projection or synaptic transmission. This suggests that normal brain cells undergo dedifferentiation as they are transformed to GBM tumor cells. It was recently shown, for example that oncogene-induced dedifferentiation of mature brain cells can lead to the development of gliomas in mice [45].

That the most abundant mRNAs in a GBM tumor cell include the shorter, mitochondrial enzymes-encoding transcripts but not the longer, glycolytic enzymes-encoding transcripts, whereas both these subsets of transcripts are among the most abundant in a normal brain cell, suggests a previously unrecognized mode for the GBM tumor and normal brain to differentially regulate metabolism. While supported by several recent observations, these metabolic differences between normal and tumor cells are unexpected considering the traditional understanding of the Warburg effect. Warburg observed that while most normal cells produce energy primarily by mitochondrial metabolism fueled by low rates of glycolysis, many types of cancer cells rely instead on aerobic glycolysis, a form of glucose metabolism that involves higher rates of glucose consumption [46]. Positron emission tomography (PET) imaging of many organs, for example, can distinguish between a tumor and its surrounding tissue by mapping the glucose uptake levels across the organ [47]. The tumor cell's increased rates of glycolysis and production of glycolytic intermediates are not necessarily linked to a higher flux of the intermediates into the mitochondrial metabolic pathways, but rather into non-oxidative metabolic pathways, even when oxygen is abundant [48], [49].

Recent observations suggest, however, that the aerobic glycolytic and mitochondrial oxidative metabolic pathways are coupled, and that it is a change in this coupling that differentiates the GBM tumor's metabolism from that of the normal brain. It is known that both the glucose and mitochondrial metabolic pathways are required for normal brain function. The glucose uptake of a normal brain cell, for example, is higher than that of most normal cells. A brain tumor's PET image that is obtained with labeled glucose or glucose analog molecules, while useful in mapping the tumor's metabolism when correlated with a magnetic resonance image or an x-ray computer tomography scan, is limited in discriminating the tumor from its surroundings [50]. Advancing the understanding of the Warburg effect, it was recently shown that GBM cell lines that exhibit aerobic glycolysis *in vitro* use the glycolytic intermediates not just for energy production but also for biosynthesis that is coupled with an uptake of mitochondrial metabolic intermediates [51]. Moreover, it was shown *in vivo* that human GBM tumors in mice brains oxidize glucose in the mitochondria [52], and derive a substantial fraction of the energetic intermediates from substrates other than glucose [53]. A similar coupling was recently hinted at when it was shown that during its juvenile growth phase, the fruit fly uses mitochondrial oxidative metabolism [54] in addition to aerobic glycolysis [55].

Our observation that both the GBM tumor and normal brain overexpress transcripts that are associated with the MRCC I is also in agreement with the recent observation that depletion of the insulin-like growth factor 2 mRNA-binding protein 2, which is known to interact with the transcripts that encode the MRCC I, reduces oxygen consumption by and impairs proliferation of GBM cell lines [56]. That transcripts that are involved in COX activity are among the most abundant not only in the normal brain but also in the GBM tumor, is also in agreement with the recent observation that a GBM tumor's greater COX activity correlates with a patient's shorter survival time [57].

Our observation that transcripts associated with glucose metabolism are among the most abundant in the normal brain but not the GBM tumor does not imply a decrease in the tumor cell's rates of glycolysis and production of glycolytic intermediates relative to those of the normal cells. This is because key glycolytic enzymes, such as PFK, are regulated by posttranslational processes, such as phosphorylation and the binding of allosteric effectors. This observation, however, cannot be explained by DNA copy-number alterations (CNAs) in the tumor relative to the normal genome alone [58]. For example, loss of chromosome 10 is observed in about 40% of GBM tumors. We found the key glycolytic enzyme-encoding gene *PFKP* that is located at the short arm of chromosome 10, overexpressed in <20% of the GBM tumor tissue samples but >20% of the normal brain samples. Overexpression of this significantly longer-than-typical gene in the normal brain but not the GBM tumor, therefore, cannot be explained by the tumor's frequent loss of chromosome 10 alone.

Note that similarly, amplification of the *CDK4* locus on chromosome 12 is observed in about 15% of GBM tumors. We found this cyclin-dependent kinase-encoding gene overexpressed in >20% of the GBM tumor tissue samples but <20% of the normal brain samples. Overexpression of this gene in the GBM tumor but not the normal brain, therefore, cannot be explained by the tumor's frequent amplification of the *CDK4* locus alone.

Taken together, we propose a previously unrecognized mode for the GBM tumor and normal brain to differentially regulate metabolism in a transcript length-dependent manner: The physical balancing of the length of a transcript with the time period of the cell cycle contributes to, and possibly regulates the biological balancing of cellular metabolism with proliferation, differently so in the GBM tumor than in the normal brain.

### Hypothesis from mathematical modeling of evolutionary forces that act upon transcript length in the manner of the restoring force of the harmonic oscillator is supported

Third, we found that the SVD identifies the length distribution functions of the human and yeast global sets and metabolic ontology subsets of transcripts, as well as human subsets of transcripts of similar expression in response to a normal brain's transformation to a GBM tumor, as asymmetric generalized coherent states. Note that, in general, it is not necessarily possible to identify a distribution function from data that sample the function [19]. This is because identifying a distribution function is mathematically equivalent to estimating the *infinite* number of moments that are associated with the function. The SVD, however, identified the transcript length distribution functions from the DNA microarray data and with no *a-priori* assumptions. This is because the *finite* and few most significant eigenvectors and corresponding eigenvalues that were uncovered by the SVD of the length distribution data of each of the sets and subsets of transcripts fit a series of orthogonal asymmetric Hermite functions and a corresponding geometric series, which are known to be among the eigenfunctions and corresponding eigenvalues, respectively, of an asymmetric generalized coherent state [25].

By identifying the transcript length distribution functions, the SVD also identifies the underlying phenomenological forces that act upon the lengths (or gel migration distances) of the transcripts. From the fit of the distribution, or profile of a single transcript to the asymmetric Gaussian 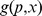 of Equation (4) it follows that the force acting upon the transcript's length (or gel migration distance) is linearly proportional to
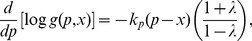
(16)i.e., the force is linearly proportional to and oppositely directed to the displacement 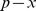 from the peak of the transcript's profile at 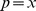, acting upon the transcript's length (or gel migration distance) in the manner of the restoring force of the harmonic oscillator [59]. From the asymmetry *s* of the generalized Hooke's constant 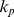 of Equation (5), the magnitude of the force when acting upon lengths that are lesser (or migration distances that are greater) than the peak, i.e., 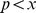, is *s* times its magnitude when acting upon lengths that are greater (or migration distances that are lesser) than the peak, i.e., 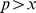.

In the limit where the multiple transcripts of a single gene are identical in length, the profile of a single transcript represents the distribution of the gel migration distances of the transcript, and the phenomenological force that underlies this distribution acts upon the transcript's gel migration distance alone. Previously, we suggested that the asymmetry of the profile of a single transcript might be due to an asymmetry in the gel electrophoresis thermal broadening of a moving, rather than a stationary, band of identical mRNA molecules [25]. In the absence of an electric field, the thermal broadening or Brownian motion of the band of identical mRNA molecules is such that the distribution of the molecules fits a Gaussian. In the presence of an electric field, the band's displacement along the axis of the electric field is linearly proportional to the time interval [60], whereas the width of the band's thermal broadening is linearly proportional to the square root of the time interval [20]. As a result, the peak of the band appears to be moving toward the front of the band and away from its back, and the distribution of the mRNA molecules fits an asymmetric Gaussian. Note that prior theory, simulation and measurement of DNA band broadening in gel electrophoresis have shown that the broadening of a moving band can be different from that of a stationary band, but have not suggested an asymmetry [61]-[63]. We concluded that mathematical modeling of DNA microarray data can be used to predict physical, not just biological modes of regulation that govern the activities of DNA and RNA [64].

In the limit where the distribution of the transcript length of a single gene spans the lengths of the UTR and the poly(A) tail of the transcript, however, the profile of a single transcript represents the distribution of the lengths and not just the gel migration distances of the transcript. Hurowitz *et al* estimated the precision of the gel electrophoresis measurement to be approximately 5% of a transcript's length, for both the human and yeast [12], [13]. The average UTR and poly(A) tail lengths were estimated to be approximately 1,250 and 200 nt for the human genes, and 250 and 60 nt for the yeast genes, respectively, and independent of the transcript's length. Therefore, a distribution of the length of a transcript that spans the average UTR and poly(A) tail lengths can be expected to affect the profiles of most human and yeast transcripts in the data sets we analyze. In this case, the phenomenological force that underlies the profile of a single transcript does act upon the single transcript's length (and not just its gel migration distance).

Similarly, from the fit of the distribution of the peaks of the transcript profiles to the asymmetric Gaussian 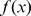 of Equation (4) it follows that the force acting upon the peak of a transcript's profile is linearly proportional to
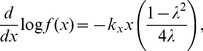
(17)i.e., the force is linearly proportional to and oppositely directed to the displacement of the peak from the equilibrium 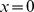, in the manner of the restoring force of the harmonic oscillator. From the asymmetry *s* of the generalized Hooke's constant 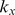 of Equation (3), the magnitude of the force when acting upon lengths that are lesser than the equilibrium, i.e., 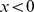, is *s* times its magnitude when acting upon lengths that are greater than the equilibrium, i.e., 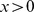.

For each set or subset of transcripts, the asymmetry 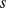 in the generalized Hooke's constant 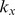 that acts upon the displacement of the peak of the transcript's profile from the equilibrium 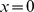 is the same as the asymmetry in the generalized Hooke's constant 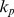 that acts upon the displacement of a transcript's length from the peak 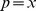. Note that the gel migration distance of a transcript is proportional to the logarithm of the inverse of the transcript's length in nucleotides [60]. Therefore, the asymmetry in 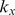 and 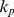 where the transcript lengths are measured in gel slices underestimates the asymmetry where the lengths are in nucleotides. Previously, we hypothesized that the asymmetry is the result of two competing evolutionary forces. One force acts to minimize the costs associated with transcriptional as well as posttranscriptional processes, such as translation, and therefore also the lengths of gene transcripts. The other force acts to maximize the information content of the genes and their functional specificity, and therefore also their mRNA lengths. For example, there is evidence that the eukaryotic PFK enzymes evolved from the prokaryotic enzymes via gene duplication, and that this doubling of the molecular weight of PFK is linked to the creation of sites, beyond the sites that are found in prokaryotic PFKs, for allosteric effectors to regulate the eukaryotic PFK activity [29]. Acting upon the displacement of a transcript's length from the peak of the transcript's profile at 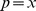, the two forces balance at the peak of the distribution 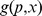, i.e., at 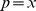. Acting upon the displacement of the peak of the transcript's profile from the equilibrium 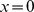, the forces balance at the equilibrium of the distribution 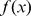, i.e., at 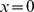. That the SVD identifies the length distribution functions of the human and yeast sets and subsets of transcripts as asymmetric generalized coherent states, therefore, supports our previous hypothesis from mathematical modeling of evolutionary forces that determine transcript lengths, which act in the manner of the restoring force of the harmonic oscillator [25].

Previously we used the SVD to uncover a global correlation, and predict causal coordination between eukaryotic DNA replication origin activity and mRNA expression [65], [66]. We experimentally showed that origin licensing, i.e., the assembly of pre-replicative complexes at DNA replication origins, decreases the expression of genes with origins near their 3′ ends, revealing that downstream origins can regulate the expression of upstream genes. This confirmed our prediction, and demonstrated that mathematical modeling of DNA microarray data can be used to correctly predict previously unknown biological modes of regulation [67].

Here we used the SVD to identify the length distribution functions of sets and subsets of eukaryotic mRNA transcripts from DNA microarray data and with no *a-priori* assumptions, and reveal global relations among transcript length, cellular metabolism and tumor development. The global relations suggest a previously unrecognized physical mode for tumor and normal cells to differentially regulate metabolism in a transcript length-dependent manner. The identified distribution functions support a previous hypothesis from mathematical modeling of evolutionary forces that act upon transcript length in the manner of the restoring force of the harmonic oscillator.

Additional possible applications of SVD analyses of mRNA transcript length distribution data, measured by using DNA sequencing or microarray hybridization technologies, include comparisons among (*i*) different types of normal cells, e.g., neurons; (*ii*) different types of tumor cells of different, e.g., tissues of origin, pathological diagnoses and prognoses, or responses to treatments; or (*iii*) normal or tumor cells at different stages of, e.g., development or response to chemical perturbations by, e.g., carcinogens or anti-cancer drugs. Identifying and comparing the length distribution functions of the sets and subsets of transcripts that these cells express may reveal previously unrecognized relations between, and possibly even modes of co-regulation of cellular diversity and transcript length.

## Supporting Information

Appendix S1 **Supporting Figures S1, S2, S3 and S4 and Tables S1, S2, S3, S4 and S5.** A PDF format file, readable by Adobe Acrobat Reader.
(PDF)

Notebook S1 **SVD Identification of Transcript Length Distribution Functions from DNA Microarray Data.** A PDF format file, readable by Adobe Acrobat Reader. The corresponding Mathematica 8.0.1 code file, executable by Mathematica and readable by Mathematica Player, is available at http://www.alterlab.org/GBM_metabolism/.
(PDF)

Dataset S1
**Human Transcript Lengths.** A tab-delimited text format file, readable by both Mathematica and Microsoft Excel, reproducing the profiles of mRNA abundance levels [12] as well as the GO annotations [14] of the 4,109 human genes with no missing data across 50 agarose gel slices, spanning an electrophoretic migration range of 26–124 mm and the corresponding transcript length range of 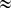
6,400–500 nt. A transcript is additionally annotated as overexpressed in either the normal brain or the GBM tumor if it is in the group of 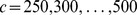 most expressed among the 4,109 transcripts in at least 20% of the normal brain or GBM tumor samples from TCGA [16], [17], respectively.(TXT)

Dataset S2
**Yeast Transcript Lengths.** A tab-delimited text format file, readable by both Mathematica and Microsoft Excel, reproducing the profiles of mRNA abundance levels [13], GO annotations [14] and DNA damage response annotations [40] of the 3,620 *Saccharomyces cerevisiae* ORFs with no missing data across 30 agarose gel slices, spanning electrophoretic migration of 42–100 mm and transcript lengths of 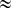
4,500–300 nt.(TXT)

Dataset S3
**Human Gene Lengths.** A tab-delimited text format file, readable by both Mathematica and Microsoft Excel, reproducing the UCSC human genome browser maximum and minimum gene lengths [30], [31] and GO annotations [14] of the 11,631 human genes. A gene is additionally annotated as overexpressed in either the normal brain or the GBM tumor if it is in the group of 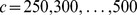 most expressed among the 11,631 genes in at least 20% of the normal brain or GBM tumor samples from TCGA [16], [17], respectively. The normal brain and the GBM tumor gene expression data sets, reproducing the abundance levels of mRNA transcripts of the 11,631 human genes from ten TCGA normal brain tissue samples and 529 TCGA GBM tumor samples, respectively, are available at http://www.alterlab.org/GBM_metabolism/.(TXT)
