# Supplementary material for: SVD Identifies Transcript Length Distribution Functions from DNA Microarray Data and Reveals Evolutionary Forces Globally Affecting GBM Metabolism
Source: PLoS One. 2013 Nov 25;8(11):e78913. doi: 10.1371/journal.pone.0078913 (PMC3839928; doi:10.1371/journal.pone.0078913)
Supplement: Appendix S1 — Supporting Figures S1, S2, S3 and S4 and Tables S1, S2, S3, S4 and S5. A PDF format file, readable by Adobe Acrobat Reader. (PDF) [file pone.0078913.s001.pdf]

Supporting Figures S1–S4 and Tables S1–S5

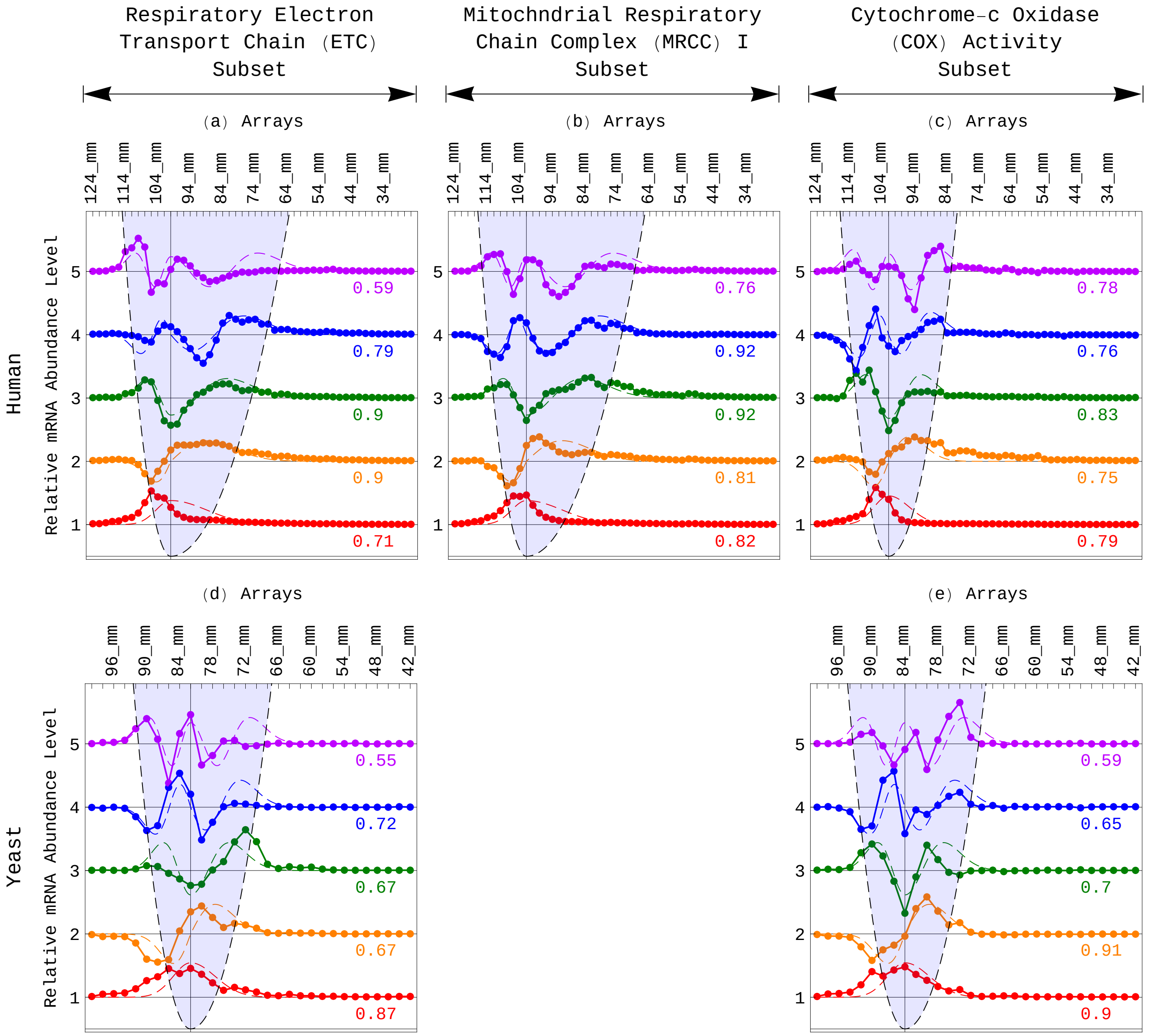

Figure S1. Eigenvectors of the transcript length distribution data of the human and yeast mitochondrial metabolism subsets. (a) The first (red) through fifth (violet) eigenvectors of the human respiratory electron transport chain (ETC) (GO:0022904) subset of transcripts. The equilibrium is shifted from that of the human global set to the greater migration distance of 100 mm. (b) Eigenvectors of the human mitochondrial respiratory chain complex (MRCC) I (GO:0004129) subset. (c) Eigenvectors of the human cytochrome-c oxidase (COX) activity (GO:0005747) subset. The equilibria of the human COX activity and MRCC I subsets are shifted from those of the human global set and respiratory ETC subset to the greater migration distance of 102 mm and lesser transcript length of ≈925±75 nt. (d) Eigenvectors of the yeast respiratory ETC subset. The equilibrium is shifted from that of the yeast global set to the greater migration distance of 82 mm. (e) Eigenvectors of the yeast COX activity subset. The equilibrium is shifted to the even greater migration distance of 84 mm and lesser transcript length of 775±75 nt.

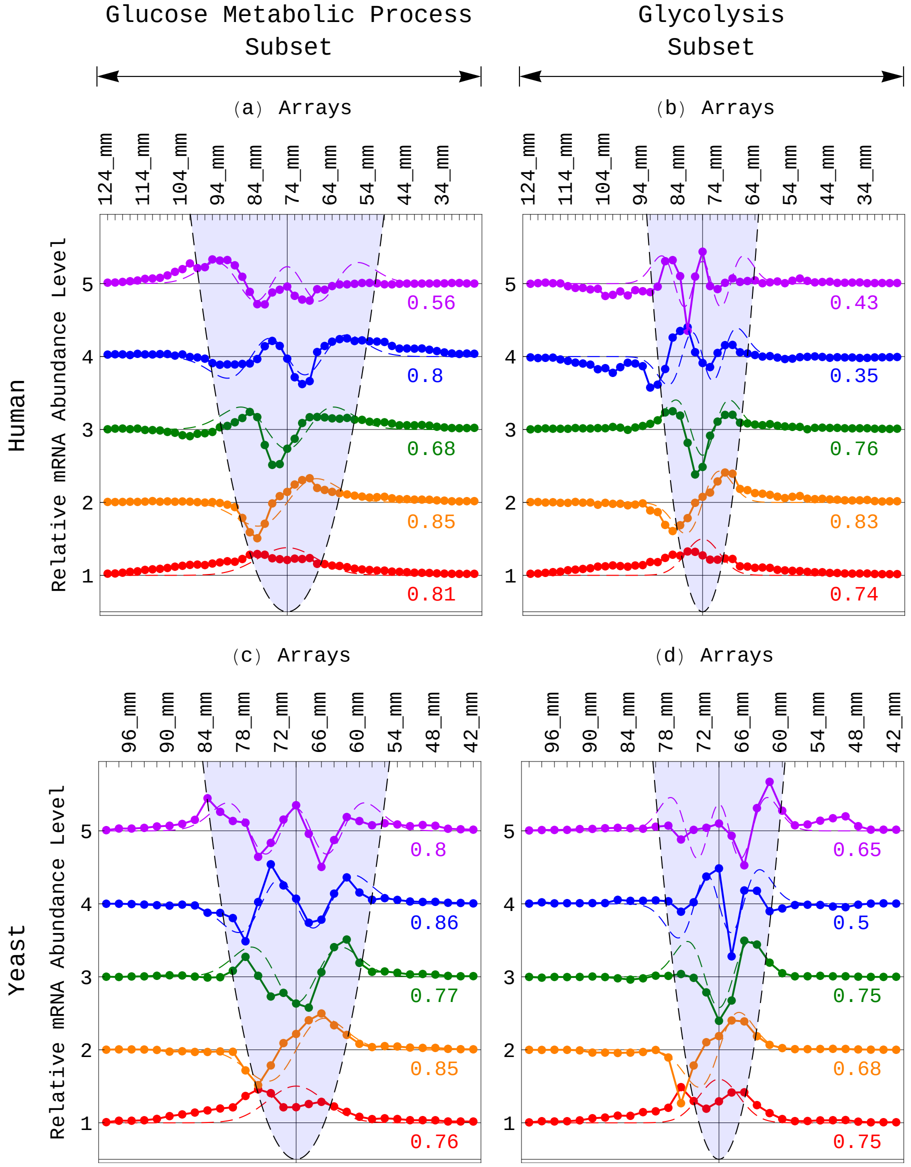

Figure S2. Eigenvectors of the transcript length distribution data of the human and yeast glucose metabolism subsets. (*a*) Eigenvectors of the human glucose metabolic process (GO:0006006) subset of transcripts. The equilibrium is shifted from that of the human global set to the lesser migration distance of 76 mm and greater transcript length of ≈2,175±125 nt. (*b*) Eigenvectors of the human glycolysis (GO:0006096) subset. The equilibrium is at the migration distance of 78 mm and the transcript length of 2,050±125 nt. (*c*) Eigenvectors of the yeast glucose metabolic process subset. The equilibrium is shifted from that of the yeast global set to the lesser migration distance of 70 mm and greater transcript length of 1,425±125 nt. (*d*) Eigenvectors of the yeast glycolysis subset. The equilibrium is at the same migration distance of 70 mm as that of the yeast glucose metabolic process subset.

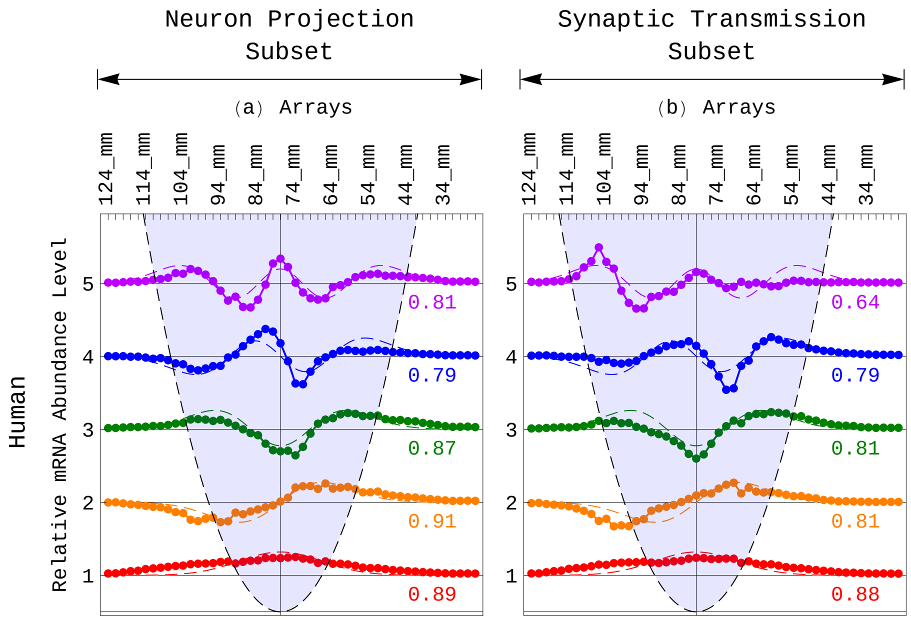

Figure S3. Eigenvectors of the transcript length distribution data of the human brain activity subsets. (*a*) Eigenvectors of the human neuron projection (GO:0043005) subset of transcripts. The equilibrium is shifted from that of the human global set to the lesser migration distance of 78 mm and greater transcript length of ≈2,050±100 nt. (*b*) Eigenvectors of the human synaptic transmission (GO:0007268) subset. The equilibrium is at the migration distance of 80 mm and the transcript length of 1,875±100 nt.

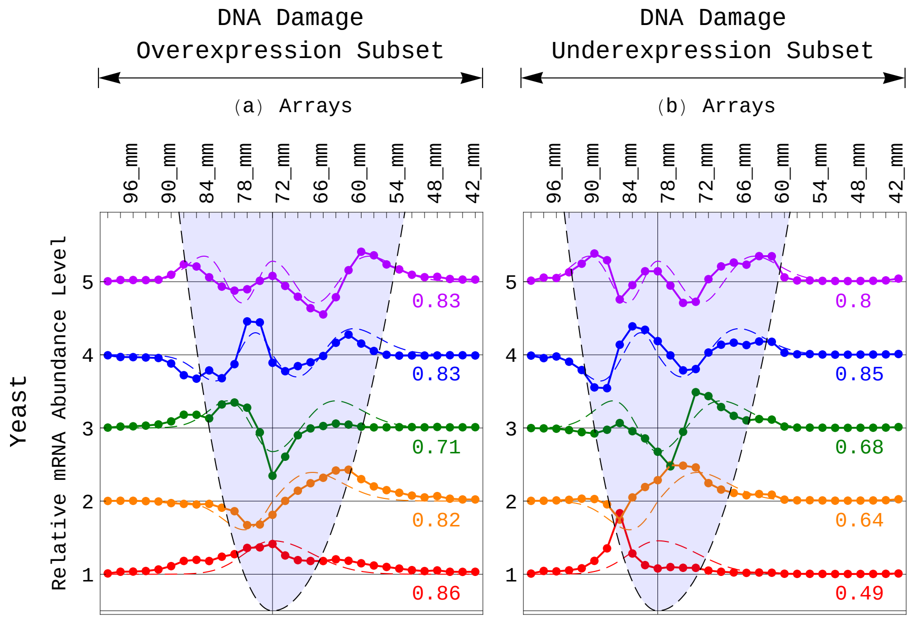

Figure S4. Eigenvectors of the transcript length distribution data of the yeast DNA damage response subsets. (*a*) Eigenvectors of the subset of yeast transcripts that are overexpressed in response to DNA damage. The equilibrium is shifted from that of the yeast global set to the lesser migration distance of 74 mm and greater transcript length of ≈1,250±100 nt. (*b*) Eigenvectors of the subset of transcripts that are underexpressed in response to DNA damage. The equilibrium is shifted to the greater migration distance of 80 mm and lesser transcript length of 950±100 nt.

Transcript Subset		Human			Yeast		
		$x = 0$	k	s	$x = 0$	k	s
Gene Ontology	Translation	96	2	2	84	2	2
	Ribosome	100	3	2	84	3	2
	Respiratory ETC	100	1	6	82	2	2
	MRCC I	102	1	6			
	COX Activity	102	3	2	84	2	2
	Glucose Metabolic Process	76	2	1	70	2	1
	Glycolysis	78	6	1	70	4	1
	Neuron Projection	78	1	1			
	Synaptic Transmission	80	1	1			
Overexpression	Normal ∩ Tumor	96	1	4			
	Tumor \ Normal	90	1	2			
	Normal \ Tumor	80	1	1			

Table S1. The generalized Hooke's constant of subsets of human and yeast transcripts. The generalized Hooke's constant k_x of Equation (3) is defined by its equilibrium $x = 0$ gel migration distance in mm, its magnitude k relative to that of the corresponding global set, and its asymmetry s. The subsets of human transcripts that are most abundant in both the GBM tumor and normal brain, the GBM tumor only or the normal brain only are considered at the overexpression cutoff of $c = 250$.

Table S2 (on p. A-5). Typical gene ontology (GO) annotations significantly enriching the human subsets of transcripts and genes overexpressed in both the GBM tumor and normal brain, the normal brain overall or the normal brain only. The P-value of a given enrichment is calculated assuming hypergeometric probability distribution of the B annotations among the A transcripts or genes in the global set, and of the subset of $b \subseteq B$ annotations among the subset of $a \subseteq A$ transcripts or genes, $P(A, a, B, b) = \binom{A}{a}^{-1} \sum_{i=b}^{a} \binom{B}{i}\binom{A-B}{a-i}$. These enrichments of the subsets at the overexpression cutoffs of $c = 300, \ldots, 500$ are consistent with the enrichments of the corresponding subsets at the overexpression cutoff of $c = 250$ (Table 3). None of the multiple GO annotations consistently enrich the human subsets of transcripts and genes that are overexpressed in the GBM tumor only. None of the multiple GO annotations consistently enrich the human subsets of transcripts and genes that are overexpressed in the GBM tumor overall beyond those that enrich the subsets that are overexpressed in both the GBM tumor and normal brain.

c	Overexpression Subset	Gene Ontology	Global Transcript Set				Global Gene Set			
			a	B	b	P-value	a	B	b	P-value
300	Normal ∩ Tumor	Translation	239	178	38	4.9×10^{-13}	250	380	72	7.3×10^{-49}
		Ribosome		78	28	5.1×10^{-16}		155	58	7.3×10^{-58}
		Respiratory ETC		55	25	2.5×10^{-17}		89	29	6.2×10^{-27}
		MRCC I		25	12	3.0×10^{-9}		34	10	1.5×10^{-9}
		COX Activity		14	9	1.0×10^{-8}		20	9	1.2×10^{-10}
	Normal	Glucose Metabolic Process	360	100	18	2.3×10^{-3}	377	187	14	3.1×10^{-3}
		Glycolysis		29	9	5.7×10^{-4}		59	6	1.2×10^{-2}
	Normal \ Tumor	Neuron Projection	121	259	23	1.2×10^{-6}	127	534	26	9.3×10^{-11}
		Synaptic Transmission		238	20	1.5×10^{-5}		535	29	4.3×10^{-13}
350	Normal ∩ Tumor	Translation	279	178	43	3.1×10^{-14}	284	380	77	3.6×10^{-50}
		Ribosome		78	28	3.0×10^{-14}		155	58	2.3×10^{-54}
		Respiratory ETC		55	27	5.9×10^{-18}		89	30	1.2×10^{-26}
		MRCC I		25	13	1.2×10^{-9}		34	10	5.1×10^{-9}
		COX Activity		14	10	1.4×10^{-9}		20	9	3.6×10^{-10}
	Normal	Glucose Metabolic Process	420	100	20	2.3×10^{-3}	428	187	17	5.4×10^{-4}
		Glycolysis		29	9	1.7×10^{-3}		59	8	1.4×10^{-3}
	Normal \ Tumor	Neuron Projection	141	259	24	5.3×10^{-6}	144	534	27	3.3×10^{-10}
		Synaptic Transmission		238	22	1.4×10^{-5}		535	32	5.7×10^{-14}
400	Normal ∩ Tumor	Translation	326	178	48	4.3×10^{-15}	317	380	80	1.5×10^{-49}
		Ribosome		78	30	2.8×10^{-14}		155	59	7.6×10^{-53}
		Respiratory ETC		55	32	5.0×10^{-22}		89	31	1.5×10^{-26}
		MRCC I		25	17	7.9×10^{-14}		34	10	1.5×10^{-8}
		COX Activity		14	11	2.0×10^{-10}		20	9	9.6×10^{-10}
	Normal	Glucose Metabolic Process	471	100	20	8.4×10^{-3}	489	187	19	3.4×10^{-4}
		Glycolysis		29	9	3.9×10^{-3}		59	10	1.6×10^{-4}
	Normal \ Tumor	Neuron Projection	145	259	29	1.3×10^{-8}	172	534	27	1.9×10^{-8}
		Synaptic Transmission		238	25	5.3×10^{-7}		535	36	1.1×10^{-14}
450	Normal ∩ Tumor	Translation	371	178	51	8.6×10^{-15}	360	380	83	4.0×10^{-48}
		Ribosome		78	31	1.3×10^{-13}		155	62	1.5×10^{-53}
		Respiratory ETC		55	33	1.8×10^{-21}		89	32	4.1×10^{-26}
		MRCC I		25	17	6.9×10^{-13}		34	11	3.3×10^{-9}
		COX Activity		14	11	8.0×10^{-10}		20	9	3.0×10^{-9}
	Normal	Glucose Metabolic Process	538	100	20	3.2×10^{-2}	550	187	23	2.5×10^{-5}
		Glycolysis		29	9	9.3×10^{-3}		59	12	1.6×10^{-5}
	Normal \ Tumor	Neuron Projection	167	259	31	2.5×10^{-8}	190	534	32	1.4×10^{-10}
		Synaptic Transmission		238	32	7.2×10^{-10}		535	34	6.7×10^{-12}
500	Normal ∩ Tumor	Translation	412	178	54	8.7×10^{-15}	401	380	85	3.4×10^{-46}
		Ribosome		78	33	5.1×10^{-14}		155	63	6.9×10^{-52}
		Respiratory ETC		55	35	2.2×10^{-22}		89	35	1.9×10^{-28}
		MRCC I		25	18	1.8×10^{-13}		34	12	6.7×10^{-10}
		COX Activity		14	11	2.5×10^{-9}		20	10	2.9×10^{-10}
	Normal	Glucose Metabolic Process	592	100	24	6.7×10^{-3}	607	187	23	1.1×10^{-4}
		Glycolysis		29	12	3.5×10^{-4}		59	12	4.3×10^{-5}
	Normal \ Tumor	Neuron Projection	180	259	33	1.2×10^{-8}	206	534	32	1.2×10^{-9}
		Synaptic Transmission		238	33	1.3×10^{-9}		535	33	2.9×10^{-10}

Table S2 (caption on p. A-4).

Gene Subset			M	Maximum Lengths $y(M)+x_0$	Maximum Lengths P-value	Minimum Lengths $y(M)+x_0$	Minimum Lengths P-value
Gene Ontology	Neuron Projection		534	147884	4.6×10^{-3}	70714	8.9×10^{-3}
	Synaptic Transmission		535	127673	8.2×10^{-3}	68368	1.0×10^{-2}
Normal ∩ Tumor Overexpression	c	250	204	22836	3.9×10^{-2}	12356	4.3×10^{-2}
		300	250	25155	3.5×10^{-2}	14531	4.2×10^{-2}
		350	284	27150	3.4×10^{-2}	16009	4.2×10^{-2}
		400	317	27735	3.2×10^{-2}	16782	4.1×10^{-2}
		450	360	27871	2.8×10^{-2}	17036	3.7×10^{-2}
		500	401	30961	2.9×10^{-2}	17800	3.6×10^{-2}

Table S3. Human subsets of average maximum and minimum gene lengths significantly lesser than those of the global set. The P-value of Equation (11) is calculated for the average maximum or minimum gene length $y(M)+x_0$ in nucleotides of each subset of M genes relative to the average maximum and minimum gene lengths of x_0=67,448 and 37,091 nt, respectively, of the global set. The subsets of transcripts that are most abundant in both the normal brain and GBM tumor are considered at each of the overexpression cutoffs of $c = 250, 300, \ldots, 500$.

Gene Subset			L	Maximum Lengths $y(L)+x_0$	Maximum Lengths P-value	Minimum Lengths $y(L)+x_0$	Minimum Lengths P-value
Gene Ontology	Translation		380	39461	9.2×10^{-3}	22790	1.3×10^{-2}
	Ribosome		155	20949	2.0×10^{-2}	13259	2.6×10^{-2}
	Respiratory ETC		89	24032	4.0×10^{-2}		
Normal ∩ Tumor Overexpression	c	250	204	22836	1.4×10^{-2}	12356	1.8×10^{-2}
		300	250	25155	1.2×10^{-2}	14531	1.5×10^{-2}
		350	284	27150	1.0×10^{-2}	16009	1.4×10^{-2}
		400	317	27735	9.1×10^{-3}	16782	1.2×10^{-2}
		450	360	27871	7.9×10^{-3}	17036	1.1×10^{-2}
		500	401	30961	7.4×10^{-3}	17800	9.8×10^{-3}
Tumor \ Normal Overexpression		250	126	37059	3.3×10^{-2}	21670	4.5×10^{-2}
		300	143	37385	2.8×10^{-2}	20104	3.7×10^{-2}
		350	177	35271	2.1×10^{-2}	18929	2.7×10^{-2}
		400	205	34814	1.8×10^{-2}	18482	2.2×10^{-2}
		450	228	41723	1.8×10^{-2}	21369	2.2×10^{-2}
		500	246	38425	1.5×10^{-2}	21348	2.0×10^{-2}

Table S4. Human subsets of average maximum and minimum gene lengths significantly lesser than those of the neuron projection subset. The P-value of Equation (12) is calculated for the average maximum or minimum gene length $y(L)+x_0$ in nucleotides of each subset of L genes relative to the average maximum and minimum gene lengths of $y(M)+x_0$=147,884 and 70,714 nt, respectively, of the neuron projection subset of M=534 genes. The subsets of transcripts that are most abundant in both the normal and tumor or the tumor only are considered at each of the overexpression cutoffs of $c = 250, 300, \ldots, 500$.

Gene Subset			L	Maximum Lengths $y(L)+x_0$	Maximum Lengths P-value	Minimum Lengths $y(L)+x_0$	Minimum Lengths P-value
Gene Ontology	Glucose Metabolic Process		187	52201	2.1×10^{-2}	28557	2.9×10^{-2}
	Glycolysis		59	49394	4.9×10^{-2}		
	Neuron Projection		534	147884	2.0×10^{-2}	70714	2.6×10^{-2}
	Synaptic Transmission		535	127673	2.1×10^{-2}	68368	2.1×10^{-2}
Normal \ Tumor Overexpression	c	250	105	74603	1.8×10^{-2}	46572	2.2×10^{-2}
		300	127	82434	1.5×10^{-2}	50520	1.7×10^{-2}
		350	144	83614	1.4×10^{-2}	48422	1.7×10^{-2}
		400	172	82013	1.4×10^{-2}	45783	1.8×10^{-2}
		450	190	94963	1.4×10^{-2}	51400	1.8×10^{-2}
		500	206	112105	1.6×10^{-2}	55631	1.6×10^{-2}

Table S5. Human subsets of average maximum and minimum gene lengths significantly greater than those of the ribosome subset. The P-value of Equation (12) is calculated for the average maximum or minimum gene length $y(L)+x_0$ in nucleotides of each subset of L genes relative to the average maximum and minimum gene lengths of $y(M)+x_0$= 20,949 and 13,259 nt, respectively, of the ribosome subset of M=155 genes. The subsets of transcripts that are most abundant in the normal brain only are considered at each of the overexpression cutoffs of $c = 250, 300, \ldots, 500$.
