# Supplementary material for: SVD Identifies Transcript Length Distribution Functions from DNA Microarray Data and Reveals Evolutionary Forces Globally Affecting GBM Metabolism
Source: PLoS One. 2013 Nov 25;8(11):e78913. doi: 10.1371/journal.pone.0078913 (PMC3839928; doi:10.1371/journal.pone.0078913)
Supplement: Notebook S1 — SVD Identification of Transcript Length Distribution Functions from DNA Microarray Data. A PDF format file, readable by Adobe Acrobat Reader. The corresponding Mathematica 8.0.1 code file, executable by Mathematica and readable by Mathematica Player, is available at http://www.alterlab.org/GBM_metabolism/. (PDF) [file pone.0078913.s002.pdf]

(* © Nicolas M. Bertagnolli, Justin A. Drake and Orly Alter 2013 *)

(* All Rights Reserved *)

(* Initialize *)

```
Clear["Global`*"]
Off[SetOptions::optnf];
SetOptions[Plot, BaseStyle → {FontFamily → "Courier", FontSize → 10}];
SetOptions[Graphics, BaseStyle → {FontFamily → "Courier", FontSize → 10}];
SetOptions[Show, BaseStyle → {FontFamily → "Courier", FontSize → 10}];
SetOptions[BarChart, BaseStyle → {FontFamily → "Courier", FontSize → 10}];

resolution = 600;
```

(* Define Path to Datasets *)

```
path = "Desktop/Oscillator/";
```

(* Identifying Transcript Length Distribution Functions from DNA Microarray Data *)

(* Read the Transcript Length Data of the Human Global Set *)

```
stream = path <> "Data/Human_Transcript_Lengths.txt";
matrix = Import[stream, "Table"];
annotations = Position[matrix[[1]], "124_mm"][[1, 1]] - 1;
{genes, arrays} = Dimensions[matrix] - {1, annotations}
Clear[stream]

{4109, 50}
```

(* Sort Transcripts by Length *)

```
genenames = Take[matrix, {2, genes + 1}, {1, annotations}];
arraynames = Take[matrix, {1, 1}, {annotations + 1, arrays + annotations}];
annotationnames = Take[matrix, {1, 1}, {1, annotations}];
matrix = Take[matrix, {2, genes + 1}, {annotations + 1, arrays + annotations}];
matrix = ToExpression[matrix];
list = Take[genenames, All, {Position[annotationnames, "Hurowitz_et_al_Length"][[1, 2]]}];
matrix = Take[Sort[Join[list, matrix, 2], OrderedQ[{#1, #2}] &], {1, genes}, {2, arrays + 1}];
Dimensions[matrix]

{4109, 50}
```

(* Create Data Raster Display *)

```
contrast = 0.5;
displaying = Table[
   If[contrast * matrix[[i, j]] > 0,
    If[contrast * matrix[[i, j]] < 1, {contrast * matrix[[i, j]], 0}, {1, 0}],
    If[contrast * matrix[[i, j]] > -1, {0, -contrast * matrix[[i, j]]}, {0, 1}]],
   {i, 1, genes}, {j, 1, arrays}];
framex = Table[{a - 0.5, Rotate[arraynames[[1, a]], Pi / 2]}, {a, 1, arrays}];
Do[If[Mod[a - 1, 5] ≠ 0, framex[[a, 2]] = Rotate["      ", Pi / 2]], {a, 1, Dimensions[framex][[1]]}]
labelx = "Arrays";
labely = "                  Human Transcripts";

g1 = Graphics[
   Raster[Reverse[Table[Insert[displaying[[i, j]], 0, 3], {i, 1, genes}, {j, 1, arrays}]]],
   Frame → True,
   FrameTicks → {None, None, framex, None},
   FrameLabel → {None, labely, labelx, ColumnForm[{"", ""}, Center]},
   PlotRange -> All, AspectRatio → GoldenRatio];
```

```
(* Calculate SVD *)

{eigenarrays, eigenabundances, eigengenes} =
  SingularValueDecomposition[matrix, Min[Dimensions[matrix]]];
eigengenes = Transpose[eigengenes];
eigenarrays = Transpose[eigenarrays];
eigenabundances = Diagonal[eigenabundances];
list =
  {1, 3, 4, 7, 9, 11, 12, 13, 14, 15, 17, 18, 21, 25, 26, 27, 28, 29, 30, 31, 33, 35, 39, 41, 45, 49, 50};
Do[{eigengenes[[list[[a]]]] = -eigengenes[[list[[a]]]]}, {a, 1, Dimensions[list][[1]]}]
Do[{eigenarrays[[list[[a]]]] = -eigenarrays[[list[[a]]]]}, {a, 1, Dimensions[list][[1]]}]
eigenarrays = Transpose[eigenarrays];
fractions = eigenabundances^2 / Sum[eigenabundances[[a]]^2, {a, 1, arrays}];
entropy = -N[Sum[fractions[[a]] * Log[fractions[[a]]], {a, 1, arrays}] / Log[arrays]];
entropy = N[Round[100 * entropy] / 100]

0.49

(* Create Left Singular Vectors Raster Display *)

contrast = 250;
displaying = Table[
    If[contrast * eigenarrays[[i, j]] > 0,
     If[contrast * eigenarrays[[i, j]] < 1, {contrast * eigenarrays[[i, j]], 0}, {1, 0}],
     If[contrast * eigenarrays[[i, j]] > -1, {0, -contrast * eigenarrays[[i, j]]}, {0, 1}]],
    {i, 1, genes}, {j, 1, arrays}];
framex = Table[{a - 0.5, Rotate[a, Pi / 2]}, {a, 1, arrays}];
Do[If[Mod[a, 5] ≠ 0, framex[[a, 2]] = Rotate["       ", Pi / 2]], {a, 1, Dimensions[framex][[1]]}]
labelx = "Left Singular Vectors";
labely = ColumnForm[{"", "", "                   Human Transcripts"}, Center];

g2 = Graphics[
    Raster[Reverse[Table[Insert[displaying[[i, j]], 0, 3], {i, 1, genes}, {j, 1, arrays}]]],
    Frame → True,
    FrameTicks → {None, None, framex, None},
    FrameLabel → {None, labely, labelx, None},
    PlotRange -> All, AspectRatio → GoldenRatio];

(* Create Singular Values Raster Display *)

contrast = 0.025;
eigenabundances = DiagonalMatrix[eigenabundances];
displaying = Table[
    If[contrast * eigenabundances[[i, j]] > 0,
     If[contrast * eigenabundances[[i, j]] < 1, {contrast * eigenabundances[[i, j]], 0}, {1, 0}],
     If[contrast * eigenabundances[[i, j]] > -1, {0, -contrast * eigenabundances[[i, j]]}, {0, 1}]],
    {i, 1, arrays}, {j, 1, arrays}];
eigenabundances = Diagonal[eigenabundances];
framex = Table[{a - 0.5, Rotate[a, Pi / 2]}, {a, 1, arrays}];
Do[If[Mod[a, 5] ≠ 0, framex[[a, 2]] = Rotate["       ", Pi / 2]], {a, 1, Dimensions[framex][[1]]}]
framey = Table[{a + 1 - 0.5, arrays - a}, {a, 0, arrays - 1}];
Do[If[Mod[a - 1, 5] ≠ 0, framey[[a, 2]] = "    "], {a, 1, Dimensions[framey][[1]]}]
labelx1 = "Right Singular Vectors";
labelx2 = ColumnForm[{"", "", "", "", "", "", "", ""}, Center];
labely = ColumnForm[{"", "", "", "", "", "Left Singular Vectors"}];

g3 = Graphics[
    Raster[Reverse[Table[Insert[displaying[[i, j]], 0, 3], {i, 1, arrays}, {j, 1, arrays}]]],
    Frame → True,
    FrameTicks → {None, framey, framex, None},
    FrameLabel → {labelx2, labely, labelx1, None},
    PlotRange -> All, AspectRatio → 1.05];
```

```
(* Create Right Singular Vectors Raster Display *)

contrast = 5;
displaying = Table[
   If[contrast * eigengenes[[i, j]] > 0,
    If[contrast * eigengenes[[i, j]] < 1, {contrast * eigengenes[[i, j]], 0}, {1, 0}],
    If[contrast * eigengenes[[i, j]] > -1, {0, -contrast * eigengenes[[i, j]]}, {0, 1}]],
   {i, 1, arrays}, {j, 1, arrays}];
framex = Table[{a - 0.5, Rotate[arraynames[[1, a]], Pi / 2]}, {a, 1, arrays}];
Do[If[Mod[a - 1, 5] ≠ 0, framex[[a, 2]] = Rotate["       ", Pi / 2]], {a, 1, Dimensions[framex][[1]]}]
framey = Table[{a + 1 - 0.5, arrays - a}, {a, 0, arrays - 1}];
Do[If[Mod[a - 1, 5] ≠ 0, framey[[a, 2]] = "    "], {a, 1, Dimensions[framey][[1]]}]
labelx1 = "Arrays";
labelx2 = ColumnForm[{"", "", "", "", "", "", "", ""}, Center];
labely = ColumnForm[{"", "", "", "", "", "", "", "", "", "", "Right Singular Vectors"}];
labely = ColumnForm[{"", "", "", "", "", "", "", "", "", "Right Singular Vectors"}];

g = Graphics[
   Raster[Reverse[Table[Insert[displaying[[i, j]], 0, 3], {i, 1, arrays}, {j, 1, arrays}]]],
   Frame → True,
   FrameTicks → {None, framey, framex, None},
   FrameLabel → {labelx2, labely, labelx1, None},
   PlotRange -> All, AspectRatio → 1.05];

(* Fit Right Singular Vectors with an Aysmmetric Parabola *)

k1 = 0.065;
k2 = 0.0325;
equi = 20;

Clear[f];
f[x_] := If[(x - equi - 1) < 0, 0.5 * k1 * (x - equi - 1) ^2, 0.5 * k2 * (x - equi - 1) ^2];
inflection = Plot[arrays - f[x + 0.5], {x, 0.5, arrays - 0.5},
   PlotStyle -> {RGBColor[0, 0, 1], Thickness[0.013]}];
g4 = Show[{g, inflection},
   PlotRange -> All, AspectRatio → 1.05];

(* Fit Eigenvectors with a Series of Asymmetric Hermite Functions *)

k1 = 0.065;
k2 = 0.0325;
equi = 20;

Clear[g, h];
h[x_, n_, gamma_] := Exp[-gamma * x^2 / 2] * HermiteH[n, Sqrt[gamma] * x] *
   Sqrt[Sqrt[gamma / Pi] / Factorial[n] / (2^n)];
g[x_, n_] := If[x - equi < 0, h[x - equi, n - 1, k1] * (1 / k1) ^0.25,
   h[x - equi, n - 1, k2] * (1 / k2) ^0.25];
normalization = Table[
   Sqrt[Sum[g[x, n]^2., {x, 0, arrays - 1}]],
   {n, 1, 5}];
correlation = Table[
   Sum[g[x, n] * eigengenes[[n, x + 1]] / normalization[[n]], {x, 0, arrays - 1}],
   {n, 1, 5}];
meancorrelation = Round[100. * Sqrt[Sum[0.2 * correlation[[n]]^2, {n, 1, 5}]]] / 100.
correlation = Round[100. * correlation] / 100.;

0.78

(* Fit Differential Equation with an Asymmetric Parabola *)

Clear[f];
f[x_] := If[(x - equi) < 0, 0.5 * k1 * (x - equi) ^2, 0.5 * k2 * (x - equi) ^2];
```

```
(* Create Selected Eigenvectors Graph Display with Fitting Graphs *)

labelx1 = "Arrays";
labelx2 = ColumnForm[{"", "", "", "", "", "", "", ""}, Center];
labely = ColumnForm[{"", "", "", "", "", "", "", "", "", "", "Relative mRNA Abundance Level"}];
framex = Table[{a - 1, Rotate[arraynames[[1, a]], Pi / 2]}, {a, 1, arrays}];
Do[If[Mod[a - 1, 5] ≠ 0, framex[[a, 2]] = Rotate["        ", Pi / 2]], {a, 1, Dimensions[framex][[1]]}]
framey = Table[{n - 0.5, "   " <> ToString[n]}, {n, 1, 5}];
framey = Table[{n - 0.5, " " <> ToString[n]}, {n, 1, 5}];
color = {
   RGBColor[0.75, 0, 1],
   RGBColor[1, 0, 0],
   RGBColor[1, 0.5, 0],
   RGBColor[0, 0.5, 0],
   RGBColor[0, 0, 1]};
points = Table[0, {n, 1, 5}];
lines = Table[0, {n, 1, 5}];
Do[{coordinates = Table[{a - 1, eigengenes[[n, a]] + n - 0.5}, {a, 1, arrays}],
  points[[n]] = Table[Point[coordinates[[a]]], {a, 1, arrays}],
  lines[[n]] = Line[coordinates]}, {n, 1, 5}]
points = Table[Graphics[{color[[Mod[n, 5] + 1]], PointSize[0.022], points[[n]]}], {n, 1, 5}];
lines = Table[Graphics[{Thickness[.005], color[[Mod[n, 5] + 1]], lines[[n]]}], {n, 1, 5}];
graphs = Table[Plot[g[x, n] / normalization[[n]] + n - 0.5, {x, 0, arrays - 1},
    PlotStyle -> {color[[Mod[n, 5] + 1]], Dashing[{0.03, 0.02}]}, PlotRange → All], {n, 1, 5}];
texts = Table[Graphics[{color[[Mod[n, 5] + 1]], Text[correlation[[n]], {40, n - 0.75}, {-1, 0}]}],
   {n, 1, 5}];
inflection = Plot[f[x], {x, equi - Sqrt[5.45 * 2 / k1], equi + Sqrt[5.45 * 2 / k2]},
   PlotStyle -> {RGBColor[0, 0, 0], Dashing[{0.03, 0.02}]},
   Filling → Top, FillingStyle → Opacity[0.1, RGBColor[0, 0, 1]]];

g5 = Show[{points, lines, graphs, texts, inflection},
   Frame -> True,
   FrameLabel -> {labelx2, labely, labelx1, None},
   FrameTicks -> {None, framey, framex, None},
   GridLines -> {{{equi, RGBColor[0, 0, 0]}},
     Join[{{0, RGBColor[0, 0, 0]}}, Table[{a - 0.5, RGBColor[0, 0, 0]}, {a, 1, 5}]]},
   PlotRange -> {-0.05, 5.45}, AspectRatio → 1.05];

(* Fit Eigenvalues with a Geometric Series *)

Clear[constant, λ];
f = FindFit[Table[fractions[[n]], {n, 2, 15}], constant * λ^x, {constant, λ}, x]

{constant → 0.12368, λ → 0.756294}

λ = 0.76;
f = FindFit[Table[fractions[[n]], {n, 2, 15}], constant * λ^x, constant, x]
constant = constant /. f;
correlation = Dot[Table[fractions[[n]], {n, 2, 15}], Table[constant * λ^n, {n, 1, 14}]] /
   Sqrt[Dot[Table[fractions[[n]], {n, 2, 15}], Table[fractions[[n]], {n, 2, 15}]]] /
  Sqrt[Dot[Table[constant * λ^n, {n, 1, 14}], Table[constant * λ^n, {n, 1, 14}]]]

{constant → 0.122266}

0.994999

(* Create Eigenvalues Bar Chart Displays with Fitting Graph *)

fractions[[2]]

0.100791

limit = 0.125;
```

```
Clear[gridx, framex, framey];
gridx = Table[a, {a, 0, limit, N[limit / 5]}];
framex = gridx;
framex = Table[{gridx[[a]], Style[Rotate[framex[[a]], Pi / 2]]}, {a, 1, 6}];
framex[[1]] = {0, Style[Rotate["0     ", Pi / 2]]};
gridx = Table[{gridx[[a]], RGBColor[0, 0, 0]}, {a, 1, 6}];
framey = Table[{a + 1, arrays - a - 35}, {a, 0, 15 - 2}];
Do[If[Mod[i, 2] == 0, framey[[i, 2]] = ""],
 {i, 1, Dimensions[framey][[1]]}]
table = Table[fractions[[arrays - a]], {a, 35, arrays - 2}];
g = BarChart[table,
   BarOrigin → Left,
   PlotRange -> {{0, fractions[[2]]}, {0.5, 15 - 1 + 0.5}},
   AspectRatio -> 1,
   Axes -> False,
   Frame -> True,
   FrameTicks -> {None, framey, framex, None},
   GridLines -> {gridx, None},
   ChartStyle → Red];

f1 = NSolve[Log[x / constant] / Log[1 / λ] + 15 == 1, x][[1]]
x1 = x /. f1;
f2 = NSolve[Log[x / constant] / Log[1 / λ] + 15 == 14, x][[1]]
x2 = x /. f2;
```

```
{x → 0.00262238}

{x → 0.0929221}
```

```
graph = Plot[Log[x / constant] / Log[1 / λ] + 15, {x, x1, x2},
   PlotRange -> {1, 14},
   PlotStyle -> {RGBColor[0, 0, 1], Thickness[0.026]}];
inset = Graphics[Show[{g, graph}, AspectRatio → 1.05]];

fractions[[1]]
```

```
0.551875
```

```
limit = 0.6;

Clear[gridx, framex, framey];
gridx = Table[a, {a, 0, limit, N[limit / 6]}];
framex = gridx;
framex = Table[{gridx[[a]], Rotate[framex[[a]], Pi / 2]}, {a, 1, 7}];
framex[[1]] = {0, Style[Rotate["0    ", Pi / 2]]};
gridx = Table[{gridx[[a]], RGBColor[0, 0, 0]}, {a, 1, 7}];
framey = Table[{a + 1, arrays - a}, {a, 0, arrays - 1}];
Do[If[Mod[a - 1, 5] ≠ 0, framey[[a, 2]] = "    "],
 {a, 1, Dimensions[framey][[1]]}]
labelx1 = ColumnForm[{
    "Eigenvalue Fraction",
    StringJoin["d = ", ToString[entropy]]}, Center];
labelx2 = ColumnForm[{"", "", "", "", "", "", "", ""}, Center];
labely = ColumnForm[{"", "", "", "", "", "Eigenvectors"}];
g = BarChart[
   Table[fractions[[arrays - a]], {a, 0, arrays - 1}],
   BarOrigin → Left,
   PlotRange -> {{0, fractions[[1]]}, {0.5, arrays + 0.25}}, AspectRatio -> 1,
   Axes -> False,
   Frame -> True,
   FrameTicks -> {None, framey, framex, None},
   FrameLabel → {labelx2, labely, labelx1, None},
   GridLines -> {gridx, None},
   ChartStyle → Red,
   BarSpacing → Small];
g6 = Graphics[Show[{g,
     Graphics[{RGBColor[1, 1, 0.8], Rectangle[{0.03, 0.5}, {0.53, 44}]}],
     Graphics[{Rectangle[{0.045, 1}, {0.52, 44.2}, inset]}]},
    AspectRatio → 1.2]];
```

```
(* Fit the Transcript Length Distribution Function of the Human Global Set *)

k1 = 0.065;
k2 = 0.0325;
equi = 20;

Clear[a, b, α, β, λ]
λ = 0.76;
f1 = NSolve[Sqrt[(α - k2 / 2) / (α + k2 / 2)] == λ, α];
f2 = FindInstance[{2 * Sqrt[(α /. f1) ^2 - β^2] == k2, β > 0}, β];
a = ((α /. f1) + (β /. f2))[[1]]
b = ((α /. f1) - (β /. f2))[[1]] * a / (a - ((α /. f1) - (β /. f2))[[1]])

0.119167

0.00225789

Clear[f, x, p];
f[x_, p_, a_, b_] :=
  If[x < p,
    N[Exp[- (k1 / k2) * a * (x - p) ^2]],
    N[Exp[-a * (x - p) ^2]]] *
   If[p < 0,
    N[Exp[- (k1 / k2) * b * p^2]],
    N[Exp[-b * p^2]]];
Clear[α, β, λ]

distribution = Sum[matrix[[n]], {n, 1, genes}];
distribution = distribution / Sort[distribution, OrderedQ[{#2, #1}] &][[1]];
correlation = Dot[distribution, Table[f[p, p, a, b], {p, -equi, arrays - equi - 1}]] /
    Sqrt[Dot[distribution, distribution]] /
   Sqrt[Dot[Table[f[p, p, a, b], {p, -equi, arrays - equi - 1}],
     Table[f[p, p, a, b], {p, -equi, arrays - equi - 1}]]];
correlation = Round[100 * correlation] / 100.

0.99

(* Create Graph Display of the Asymmetric Generalized Coherent State Fit *)

graphs = Table[
   Plot[f[x, p, a, b] / f[0, 0, a, b] * distribution[[equi + 1]],
    {x, -equi, arrays - equi - 1},
    PlotStyle → color[[Mod[p / 5, 5] + 1]],
    Axes → False,
    Frame → True,
    PlotRange → All],
   {p, -equi, arrays - equi - 1, 5}];
graph = Plot[f[p, p, a, b] / f[0, 0, a, b] * distribution[[equi + 1]],
   {p, -equi, arrays - equi - 1},
   PlotStyle → {RGBColor[0, 0, 0], Dashing[{0.03, 0.02}]},
   Filling → Bottom, FillingStyle → Opacity[0.1, RGBColor[0, 0, 1]],
   Axes → False,
   Frame → True,
   PlotRange → {-0.02, 1.02}];

coordinates = Table[{n - equi - 1, distribution[[n]]}, {n, 1, arrays}];
points = Table[Point[coordinates[[n]]], {n, 1, arrays}];
points = Graphics[{RGBColor[0, 0, 0], PointSize[0.022], points}];
lines = Graphics[{RGBColor[0, 0, 0], Line[coordinates]}];
```

```
distributionT = Graphics[{Text[ColumnForm[{"Human Global",
        StringJoin[ReplaceAll[Characters[arraynames[[1, equi + 1]]], "_" → " "]],
        ToString[correlation]}, Center], {19, 0.86}]}];
labelx1 = "Arrays";
labelx2 = ColumnForm[{"", "", "", "", "", "", "", ""}, Center];
labely = ColumnForm[{"", "", "", "", "", "", "", "", "", "", "Relative mRNA Abundance Level"},
    Center];
framex = Table[{n - equi - 1, Rotate[arraynames[[1, n]], Pi / 2]}, {n, 1, arrays}];
Do[If[Mod[n - 1, 5] ≠ 0, framex[[n, 2]] = Rotate["       ", Pi / 2],
    framex[[n, 2]] = StyleForm[framex[[n, 2]], FontColor → color[[Mod[(n + 4) / 5, 5] + 1]]]],
   {n, 1, Dimensions[framex][[1]]}];
framey = {0.2, 0.4, 0.6, 0.8, 1};
g = Show[{graphs, graph, points, lines, distributionT},
    Frame → True,
    FrameLabel → {labelx2, labely, labelx1, None},
    FrameTicks → {None, framey, framex, None},
    GridLines → {{{0, RGBColor[0, 0, 0]}}, None}];
g7 = Show[g,
    PlotRange → {-0.02, 1.02}, AspectRatio → 1.05];
Clear[a, b];
```

```
(* Display SVD Identification of Length Distribution Functions from Microarray Data *)
```

```
g = GraphicsGrid[{{g1, g2, g3, g4}, {g7, , g6, g5}}, Spacings → {-150, 75}, ImageSize → 1000];

fig1 = Show[{
    Graphics[{Rectangle[{0, 0}, {1250, 1004}, g]}, ImageSize → 1000],
    Graphics[Text[StyleForm["=", FontSize → 40], {358, 812}]],
    Graphics[Text[StyleForm[
        "(a) Singular Value Decomposition Uncovers Left Singular Vectors, Singular
          Values and Right Singular Vectors", FontSize → 12], {672, 1024}]],
    Graphics[Text[StyleForm["↓", FontSize → 100], {1125, 585}]],
    Graphics[Text[StyleForm[ColumnForm[{
          "(b) Eigenvectors Fit a", "Series of Asymmetric", "Hermite Functions"
         }, Center], FontSize → 12], {1125, 490}]],
    Graphics[Text[StyleForm["↓", FontSize → 100], {822, 585}]],
    Graphics[Text[StyleForm[ColumnForm[{
          "(c) Eigenvalues Fit a", "Geometric Series", ""}, Center], FontSize → 12], {822, 490}]],
    Graphics[Text[StyleForm["←", FontSize → 100], {595, 258}]],
    Graphics[Text[StyleForm[ColumnForm[{
          "(d) Distribution Fits an", "Asymmetric Generalized", "Coherent State"
         }, Center], FontSize → 12], {334, 490}]],
    Graphics[{Black, Line[{{461, 125}, {461, 530}, {163, 530}, {163, 125}, {461, 125}}]}],
    Graphics[{Black, Line[{{665, 125}, {665, 530}, {936, 530}, {936, 125}, {665, 125}}]}],
    Graphics[{Black, Line[{{963, 125}, {963, 530}, {1253, 530}, {1253, 125}, {963, 125}}]}]
   }]
```

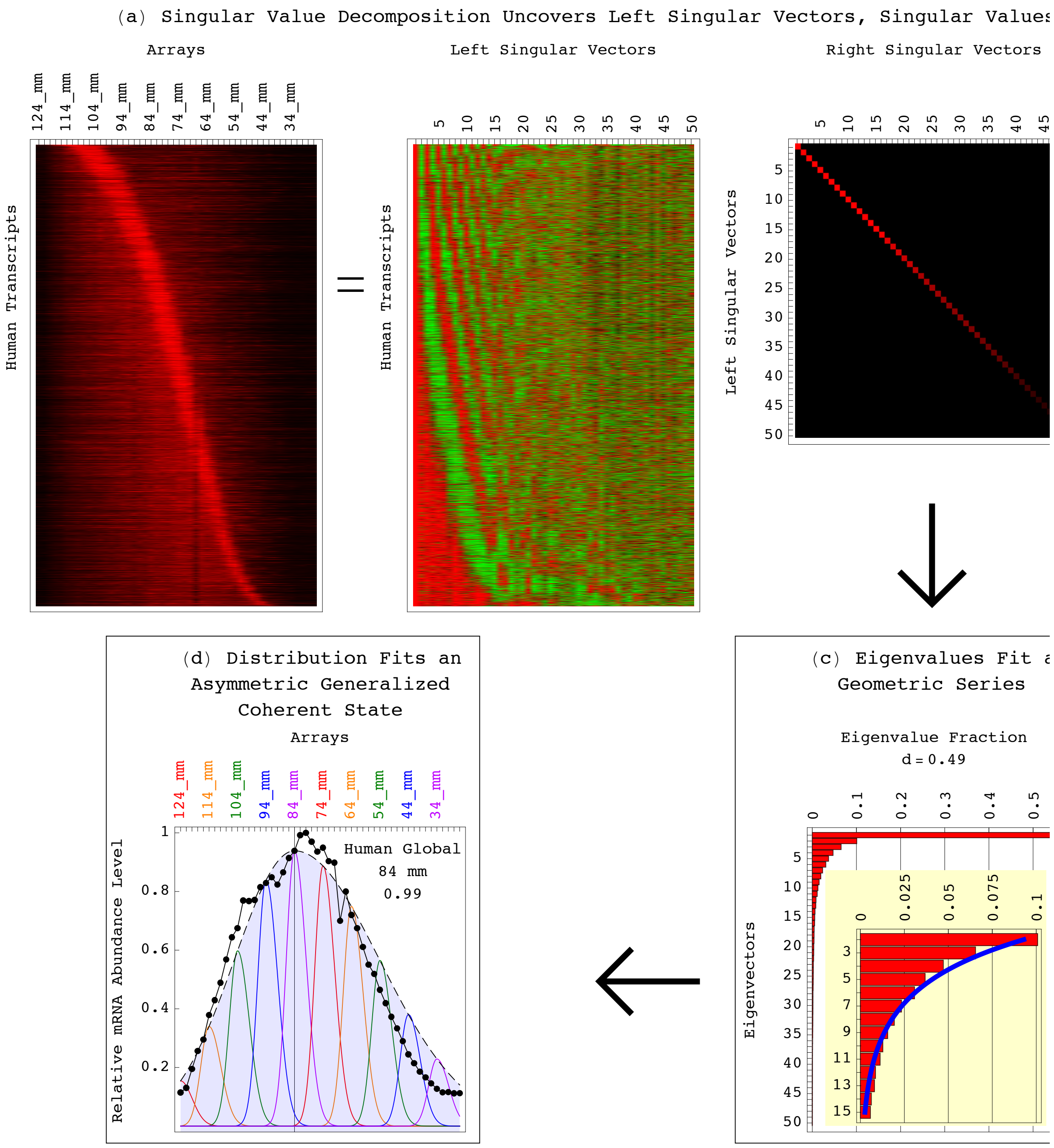


```
Export[path <> "Figures/Figure_1.pdf", fig1, "PDF", ImageSize → 1004, ImageResolution → resolution];
```

(* SVD of the Transcript Length Data of the Human Global Set *)

(* Read the Transcript Length Data of the Human Global Set *)

```
stream = path <> "Data/Human_Transcript_Lengths.txt";
matrix = Import[stream, "Table"];
annotations = Position[matrix[[1]], "124_mm"][[1, 1]] - 1;
{genes, arrays} = Dimensions[matrix] - {1, annotations}
Clear[stream]
```

```
{4109, 50}
```

```
genenames = Take[matrix, {2, genes + 1}, {1}];
arraynames = Take[matrix, {1, 1}, {annotations + 1, arrays + annotations}];
matrix = Take[matrix, {2, genes + 1}, {annotations + 1, arrays + annotations}];
matrix = ToExpression[matrix];
```

(* Calculate SVD *)

```
{eigenarrays, eigenabundances, eigengenes} =
  SingularValueDecomposition[matrix, Min[Dimensions[matrix]]];
eigengenes = Transpose[eigengenes];
eigenabundances = Diagonal[eigenabundances];
list =
  {1, 3, 4, 7, 9, 11, 12, 13, 14, 15, 17, 18, 21, 25, 26, 27, 28, 29, 30, 31, 33, 35, 39, 41, 45, 49, 50};
Do[{eigengenes[[list[[a]]]] = -eigengenes[[list[[a]]]]}, {a, 1, Dimensions[list][[1]]}]
fractions = eigenabundances^2 / Sum[eigenabundances[[a]]^2, {a, 1, arrays}];
entropy = -N[Sum[fractions[[a]] * Log[fractions[[a]]], {a, 1, arrays}] / Log[arrays]];
entropy = N[Round[100 * entropy] / 100]
```

```
0.49
```

(* Create Eigenvectors Raster Display *)

```
contrast = 5;
displaying = Table[
    If[contrast * eigengenes[[i, j]] > 0,
     If[contrast * eigengenes[[i, j]] < 1, {contrast * eigengenes[[i, j]], 0}, {1, 0}],
     If[contrast * eigengenes[[i, j]] > -1, {0, -contrast * eigengenes[[i, j]]}, {0, 1}]],
    {i, 1, arrays}, {j, 1, arrays}];
framex = Table[{a - 0.5, Rotate[arraynames[[1, a]], Pi / 2]}, {a, 1, arrays}];
Do[If[Mod[a - 1, 5] ≠ 0, framex[[a, 2]] = Rotate["       ", Pi / 2]],
  {a, 1, Dimensions[framex][[1]]}]
framey = Table[{a + 1 - 0.5, arrays - a}, {a, 0, arrays - 1}];
Do[If[Mod[a - 1, 5] ≠ 0, framey[[a, 2]] = "    "],
  {a, 1, Dimensions[framey][[1]]}]
labelx = "(a) Arrays";
labely = "Eigenvectors";

g  = Graphics[
    Raster[Reverse[Table[Insert[displaying[[i, j]], 0, 3], {i, 1, arrays}, {j, 1, arrays}]]],
    Frame → True,
    FrameTicks → {None, framey, framex, None},
    FrameLabel → {None, labely, labelx, None},
    PlotRange → All, AspectRatio → Full];
```

(* Fit Eigenvectors with an Aysmmetric Parabola *)

```
k1 = 0.065;
k2 = 0.0325;
equi = 20;

Clear[f];
f[x_] := If[(x - equi - 1) < 0, 0.5 * k1 * (x - equi - 1)^2, 0.5 * k2 * (x - equi - 1)^2];
inflection = Plot[arrays - f[x + 0.5], {x, 0.5, arrays - 0.5},
    PlotStyle → {RGBColor[0, 0, 1], Thickness[0.013]}];
g8 = Show[{g, inflection},
    PlotRange → All, AspectRatio → 1.05];
```

```
(* Fit Eigenvalues with a Geometric Series *)

Clear[constant, λ];
f = FindFit[Table[fractions[[n]], {n, 2, 15}], constant * λ^x, {constant, λ}, x]

{constant → 0.12368, λ → 0.756294}

λ = 0.76;
f = FindFit[Table[fractions[[n]], {n, 2, 15}], constant * λ^x, constant, x]
constant = constant /. f;
correlation = Dot[Table[fractions[[n]], {n, 2, 15}], Table[constant * λ^n, {n, 1, 14}]] /
    Sqrt[Dot[Table[fractions[[n]], {n, 2, 15}], Table[fractions[[n]], {n, 2, 15}]]] /
  Sqrt[Dot[Table[constant * λ^n, {n, 1, 14}], Table[constant * λ^n, {n, 1, 14}]]]

{constant → 0.122266}

0.994999

(* Create Eigenvalues Bar Chart Displays with Fitting Graph *)

fractions[[2]]

0.100791

limit = 0.125;

Clear[gridx, framex, framey];
gridx = Table[a, {a, 0, limit, N[limit / 5]}];
framex = gridx;
framex = Table[{gridx[[a]], Style[Rotate[framex[[a]], Pi / 2]]}, {a, 1, 6}];
framex[[1]] = {0, Style[Rotate["0     ", Pi / 2]]};
gridx = Table[{gridx[[a]], RGBColor[0, 0, 0]}, {a, 1, 6}];
framey = Table[{a + 1, arrays - a - 35}, {a, 0, 15 - 2}];
Do[If[Mod[i, 2] == 0, framey[[i, 2]] = ""],
 {i, 1, Dimensions[framey][[1]]}]
table = Table[fractions[[arrays - a]], {a, 35, arrays - 2}];
g = BarChart[table,
    BarOrigin → Left,
    PlotRange → {{0, fractions[[2]]}, {0.5, 15 - 1 + 0.5}}, AspectRatio → 1,
    Axes → False,
    Frame → True,
    FrameTicks → {None, framey, framex, None},
    GridLines → {gridx, None},
    ChartStyle → Red];

f1 = NSolve[Log[x / constant] / Log[1 / λ] + 15 == 1, x][[1]]
x1 = x /. f1;
f2 = NSolve[Log[x / constant] / Log[1 / λ] + 15 == 14, x][[1]]
x2 = x /. f2;

{x → 0.00262238}

{x → 0.0929221}

graph = Plot[Log[x / constant] / Log[1 / λ] + 15, {x, x1, x2},
    PlotRange → {1, 14},
    PlotStyle → {RGBColor[0, 0, 1], Thickness[0.026]}];
inset = Graphics[Show[{g, graph}, AspectRatio → 1.05]];

fractions[[1]]

0.551875

limit = 0.6;
```

```
Clear[gridx, framex, framey];
gridx = Table[a, {a, 0, limit, N[limit / 6]}];
framex = gridx;
framex = Table[{gridx[[a]], Rotate[framex[[a]], Pi / 2]}, {a, 1, 7}];
framex[[1]] = {0, Style[Rotate["0   ", Pi / 2]]};
gridx = Table[{gridx[[a]], RGBColor[0, 0, 0]}, {a, 1, 7}];
framey = Table[{a + 1, arrays - a}, {a, 0, arrays - 1}];
Do[If[Mod[a - 1, 5] ≠ 0, framey[[a, 2]] = ""],
 {a, 1, Dimensions[framey][[1]]}]
labelx = ColumnForm[{
     "(b) Eigenvalue Fraction",
     StringJoin["d = ", ToString[entropy]]}, Center];
g = BarChart[
   Table[fractions[[arrays - a]], {a, 0, arrays - 1}],
   BarOrigin → Left,
   PlotRange → {{0, fractions[[1]]}, {0.5, arrays + 0.25}}, AspectRatio → 1,
   Axes → False,
   Frame → True,
   FrameTicks → {None, framey, framex, None},
   FrameLabel → {None, None, labelx, None},
   GridLines → {gridx, None},
   ChartStyle → Red,
   BarSpacing → Small];

g9 = Graphics[Show[{g,
      Graphics[{RGBColor[1, 1, 0.8], Rectangle[{0.03, 0.5}, {0.53, 44}]}],
      Graphics[{Rectangle[{0.045, 1}, {0.52, 44.2}, inset]}]},
     AspectRatio → 1.2]];

(* Fit Eigenvectors with a Series of Asymmetric Hermite Functions *)

k1 = 0.065;
k2 = 0.0325;
equi = 20;

Clear[g, h];
h[x_, n_, gamma_] := Exp[-gamma * x ^ 2 / 2] * HermiteH[n, Sqrt[gamma] * x] *
   Sqrt[Sqrt[gamma / Pi] / Factorial[n] / (2 ^ n)];
g[x_, n_] := If[x - equi < 0, h[x - equi, n - 1, k1] * (1 / k1) ^ 0.25,
   h[x - equi, n - 1, k2] * (1 / k2) ^ 0.25];
normalization = Table[
   Sqrt[Sum[g[x, n] ^ 2., {x, 0, arrays - 1}]],
   {n, 1, 5}];
correlation = Table[
   Sum[g[x, n] * eigengenes[[n, x + 1]] / normalization[[n]], {x, 0, arrays - 1}],
   {n, 1, 5}];
meancorrelation = Round[100. * Sqrt[Sum[0.2 * correlation[[n]] ^ 2, {n, 1, 5}]]] / 100.
correlation = Round[100. * correlation] / 100.;

0.78

(* Fit Differential Equation with an Asymmetric Parabola *)

Clear[f];
f[x_] := If[(x - equi) < 0, 0.5 * k1 * (x - equi) ^ 2, 0.5 * k2 * (x - equi) ^ 2];
```

```
(* Create Selected Eigenvectors Graph Display with Fitting Graphs *)

labelx = "(c) Arrays";
labely = "Relative mRNA Abundance Level";
framex = Table[{a - 1, Rotate[arraynames[[1, a]], Pi / 2]}, {a, 1, arrays}];
Do[If[Mod[a - 1, 5] ≠ 0, framex[[a, 2]] = Rotate["       ", Pi / 2]], {a, 1, Dimensions[framex][[1]]}]
framey = Table[{n - 0.5, n}, {n, 1, 5}];
points = Table[0, {n, 1, 5}];
lines = Table[0, {n, 1, 5}];
Do[{coordinates = Table[{a - 1, eigengenes[[n, a]] + n - 0.5}, {a, 1, arrays}],
  points[[n]] = Table[Point[coordinates[[a]]], {a, 1, arrays}],
  lines[[n]] = Line[coordinates]}, {n, 1, 5}]
points = Table[Graphics[{color[[Mod[n, 5] + 1]], PointSize[0.022], points[[n]]}], {n, 1, 5}];
lines = Table[Graphics[{Thickness[.005], color[[Mod[n, 5] + 1]], lines[[n]]}], {n, 1, 5}];
graphs = Table[Plot[g[x, n] / normalization[[n]] + n - 0.5, {x, 0, arrays - 1},
    PlotStyle → {color[[Mod[n, 5] + 1]], Dashing[{0.03, 0.02}]}, PlotRange → All], {n, 1, 5}];
texts = Table[Graphics[{color[[Mod[n, 5] + 1]], Text[correlation[[n]], {40, n - 0.75}, {-1, 0}]}],
   {n, 1, 5}];
inflection = Plot[f[x], {x, equi - Sqrt[5.45 * 2 / k1], equi + Sqrt[5.45 * 2 / k2]},
   PlotStyle → {RGBColor[0, 0, 0], Dashing[{0.03, 0.02}]},
   Filling → Top, FillingStyle → Opacity[0.1, RGBColor[0, 0, 1]]];

g10 = Show[{points, lines, graphs, texts, inflection},
   Frame → True,
   FrameLabel → {None, labely, labelx, None},
   FrameTicks → {None, framey, framex, None},
   GridLines → {{{equi, RGBColor[0, 0, 0]}},
     Join[{{0, RGBColor[0, 0, 0]}}, Table[{a - 0.5, RGBColor[0, 0, 0]}, {a, 1, 5}]]},
   PlotRange → {-0.05, 5.45}, AspectRatio → 1.05];
```

```
(* SVD of the Transcript Length Data of the Human Protein Synthesis Subsets *)
```

```
(* Read the Transcript Length Data of the Human Translation Subset *)

stream = path <> "Data/Human_Transcript_Lengths.txt";
matrix = Import[stream, "Table"];
annotations = Position[matrix[[1]], "124_mm"][[1, 1]] - 1;
{genes, arrays} = Dimensions[matrix] - {1, annotations}
Clear[stream]

{4109, 50}

genenames = Take[matrix, {2, genes + 1}, {1, annotations}];
arraynames = Take[matrix, {1, 1}, {annotations + 1, arrays + annotations}];
annotationnames = Take[matrix, {1, 1}, {1, annotations}];
matrix = Take[matrix, {2, genes + 1}, {annotations + 1, arrays + annotations}];
matrix = ToExpression[matrix];
list = Take[genenames, All, {Position[annotationnames, "Translation_GO:0006412"][[1, 2]]}];
matrix = Take[Sort[Join[list, matrix, 2], OrderedQ[{#2, #1}] &], {1, Count[list, {"Y"}]},
   {2, arrays + 1}];
Dimensions[matrix]

{178, 50}
```

```
(* Calculate SVD *)

{eigenarrays, eigenabundances, eigengenes} =
  SingularValueDecomposition[matrix, Min[Dimensions[matrix]]];
eigengenes = Transpose[eigengenes];
eigenabundances = Diagonal[eigenabundances];
list = {1, 2, 3, 6};
Do[{eigengenes[[list[[a]]]] = -eigengenes[[list[[a]]]]}, {a, 1, Dimensions[list][[1]]}]
eigengenes[[5]] = eigengenes[[6]];
fractions = eigenabundances ^ 2 / Sum[eigenabundances[[a]] ^ 2, {a, 1, arrays}];
entropy = -N[Sum[fractions[[a]] * Log[fractions[[a]]], {a, 1, arrays}] / Log[arrays]];
entropy = N[Round[100 * entropy] / 100]

0.51
```

```
(* Fit Eigenvectors with a Series of Asymmetric Hermite Functions *)

k1 = 0.13;
k2 = 0.065;
equi = 14;

Clear[g, h];
h[x_, n_, gamma_] := Exp[-gamma * x^2 / 2] * HermiteH[n, Sqrt[gamma] * x] *
   Sqrt[Sqrt[gamma / Pi] / Factorial[n] / (2^n)];
g[x_, n_] := If[x - equi < 0, h[x - equi, n - 1, k1] * (1 / k1)^0.25,
   h[x - equi, n - 1, k2] * (1 / k2)^0.25];
normalization = Table[
   Sqrt[Sum[g[x, n]^2., {x, 0, arrays - 1}]],
   {n, 1, 5}];
correlation = Table[
   Sum[g[x, n] * eigengenes[[n, x + 1]] / normalization[[n]], {x, 0, arrays - 1}],
   {n, 1, 5}];
meancorrelation = Round[100. * Sqrt[Sum[0.2 * correlation[[n]]^2, {n, 1, 5}]]] / 100.
correlation = Round[100. * correlation] / 100.;

0.76

(* Fit Differential Equation with an Asymmetric Parabola *)

Clear[f];
f[x_] := If[(x - equi) < 0, 0.5 * k1 * (x - equi)^2, 0.5 * k2 * (x - equi)^2];

(* Create Selected Eigenvectors Graph Display with Fitting Graphs *)

labelx = "(d) Arrays";
framex = Table[{a - 1, Rotate[arraynames[[1, a]], Pi / 2]}, {a, 1, arrays}];
Do[If[Mod[a - 1, 5] ≠ 0, framex[[a, 2]] = Rotate["      ", Pi / 2]], {a, 1, Dimensions[framex][[1]]}]
framey = Table[{n - 0.5, n}, {n, 1, 5}];
points = Table[0, {n, 1, 5}];
lines = Table[0, {n, 1, 5}];
Do[{coordinates = Table[{a - 1, eigengenes[[n, a]] + n - 0.5}, {a, 1, arrays}],
  points[[n]] = Table[Point[coordinates[[a]]], {a, 1, arrays}],
  lines[[n]] = Line[coordinates]}, {n, 1, 5}]
points = Table[Graphics[{color[[Mod[n, 5] + 1]], PointSize[0.022], points[[n]]}], {n, 1, 5}];
lines = Table[Graphics[{Thickness[.005], color[[Mod[n, 5] + 1]], lines[[n]]}], {n, 1, 5}];
graphs = Table[Plot[g[x, n] / normalization[[n]] + n - 0.5, {x, 0, arrays - 1},
    PlotStyle → {color[[Mod[n, 5] + 1]], Dashing[{0.03, 0.02}]}, PlotRange → All], {n, 1, 5}];
texts = Table[Graphics[{color[[Mod[n, 5] + 1]], Text[correlation[[n]], {40, n - 0.75}, {-1, 0}]}],
   {n, 1, 5}];
inflection = Plot[f[x], {x, equi - Sqrt[5.45 * 2 / k1], equi + Sqrt[5.45 * 2 / k2]},
   PlotStyle → {RGBColor[0, 0, 0], Dashing[{0.03, 0.02}]},
   Filling → Top, FillingStyle → Opacity[0.1, RGBColor[0, 0, 1]]];

g11 = Show[{points, lines, graphs, texts, inflection},
   Frame → True,
   FrameLabel → {None, None, labelx, None},
   FrameTicks → {None, framey, framex, None},
   GridLines → {{{equi, RGBColor[0, 0, 0]}},
     Join[{{0, RGBColor[0, 0, 0]}}, Table[{a - 0.5, RGBColor[0, 0, 0]}, {a, 1, 5}]]},
   PlotRange → {-0.05, 5.45}, AspectRatio → 1.05];
```

```
(* Read the Transcript Length Data of the Human Ribosome Subset *)

stream = path <> "Data/Human_Transcript_Lengths.txt";
matrix = Import[stream, "Table"];
annotations = Position[matrix[[1]], "124_mm"][[1, 1]] - 1;
{genes, arrays} = Dimensions[matrix] - {1, annotations}
Clear[stream]

{4109, 50}

genenames = Take[matrix, {2, genes + 1}, {1, annotations}];
arraynames = Take[matrix, {1, 1}, {annotations + 1, arrays + annotations}];
annotationnames = Take[matrix, {1, 1}, {1, annotations}];
matrix = Take[matrix, {2, genes + 1}, {annotations + 1, arrays + annotations}];
matrix = ToExpression[matrix];
list = Take[genenames, All, {Position[annotationnames, "Ribosome_GO:0005840"][[1, 2]]}];
matrix = Take[Sort[Join[list, matrix, 2], OrderedQ[{#2, #1}] &], {1, Count[list, {"Y"}]},
    {2, arrays + 1}];
Dimensions[matrix]

{78, 50}

(* Calculate SVD *)

{eigenarrays, eigenabundances, eigengenes} =
  SingularValueDecomposition[matrix, Min[Dimensions[matrix]]];
eigengenes = Transpose[eigengenes];
eigenabundances = Diagonal[eigenabundances];
list = {1, 2, 3, 5};
Do[{eigengenes[[list[[a]]]] = -eigengenes[[list[[a]]]]}, {a, 1, Dimensions[list][[1]]}]
fractions = eigenabundances ^ 2 / Sum[eigenabundances[[a]] ^ 2, {a, 1, arrays}];
entropy = -N[Sum[fractions[[a]] * Log[fractions[[a]]], {a, 1, arrays}] / Log[arrays]];
entropy = N[Round[100 * entropy] / 100]

0.44

(* Fit Eigenvectors with a Series of Asymmetric Hermite Functions *)

k1 = 0.195;
k2 = 0.0975;
equi = 12;

Clear[g, h];
h[x_, n_, gamma_] := Exp[-gamma * x ^ 2 / 2] * HermiteH[n, Sqrt[gamma] * x] *
    Sqrt[Sqrt[gamma / Pi] / Factorial[n] / (2 ^ n)];
g[x_, n_] := If[x - equi < 0, h[x - equi, n - 1, k1] * (1 / k1) ^ 0.25,
    h[x - equi, n - 1, k2] * (1 / k2) ^ 0.25];
normalization = Table[
    Sqrt[Sum[g[x, n] ^ 2., {x, 0, arrays - 1}]],
    {n, 1, 5}];
correlation = Table[
    Sum[g[x, n] * eigengenes[[n, x + 1]] / normalization[[n]], {x, 0, arrays - 1}],
    {n, 1, 5}];
meancorrelation = Round[100. * Sqrt[Sum[0.2 * correlation[[n]] ^ 2, {n, 1, 5}]]] / 100.
correlation = Round[100. * correlation] / 100.;

0.81

(* Fit Differential Equation with an Asymmetric Parabola *)

Clear[f];
f[x_] := If[(x - equi) < 0, 0.5 * k1 * (x - equi) ^ 2, 0.5 * k2 * (x - equi) ^ 2];
```

(* Create Selected Eigenvectors Graph Display with Fitting Graphs *)

```
labelx = "(e) Arrays";
framex = Table[{a - 1, Rotate[arraynames[[1, a]], Pi / 2]}, {a, 1, arrays}];
Do[If[Mod[a - 1, 5] ≠ 0, framex[[a, 2]] = Rotate["       ", Pi / 2]], {a, 1, Dimensions[framex][[1]]}]
framey = Table[{n - 0.5, n}, {n, 1, 5}];
points = Table[0, {n, 1, 5}];
lines = Table[0, {n, 1, 5}];
Do[{coordinates = Table[{a - 1, eigengenes[[n, a]] + n - 0.5}, {a, 1, arrays}],
  points[[n]] = Table[Point[coordinates[[a]]], {a, 1, arrays}],
  lines[[n]] = Line[coordinates]}, {n, 1, 5}]
points = Table[Graphics[{color[[Mod[n, 5] + 1]], PointSize[0.022], points[[n]]}], {n, 1, 5}];
lines = Table[Graphics[{Thickness[.005], color[[Mod[n, 5] + 1]], lines[[n]]}], {n, 1, 5}];
graphs = Table[Plot[g[x, n] / normalization[[n]] + n - 0.5, {x, 0, arrays - 1},
     PlotStyle → {color[[Mod[n, 5] + 1]], Dashing[{0.03, 0.02}]}, PlotRange → All], {n, 1, 5}];
texts = Table[Graphics[{color[[Mod[n, 5] + 1]], Text[correlation[[n]], {40, n - 0.75}, {-1, 0}]}],
    {n, 1, 5}];
inflection = Plot[f[x], {x, equi - Sqrt[5.45 * 2 / k1], equi + Sqrt[5.45 * 2 / k2]},
    PlotStyle → {RGBColor[0, 0, 0], Dashing[{0.03, 0.02}]},
    Filling → Top, FillingStyle → Opacity[0.1, RGBColor[0, 0, 1]]];

g12 = Show[{points, lines, graphs, texts, inflection},
    Frame → True,
    FrameLabel → {None, None, labelx, ""},
    FrameTicks → {None, framey, framex, None},
    GridLines → {{{equi, RGBColor[0, 0, 0]}},
      Join[{{0, RGBColor[0, 0, 0]}}, Table[{a - 0.5, RGBColor[0, 0, 0]}, {a, 1, 5}]]},
    PlotRange → {-0.05, 5.45}, AspectRatio → 1.05];
```

(* SVD of the Transcript Length Data of the Yeast Global Set *)

(* Read the Transcript Length Data of the Yeast Global Set *)

```
stream = path <> "Data/Yeast_Transcript_Lengths.txt";
matrix = Import[stream, "Table"];
annotations = Position[matrix[[1]], "100_mm"][[1, 1]] - 1;
{genes, arrays} = Dimensions[matrix] - {1, annotations}
Clear[stream]
```

```
{3620, 30}
```

```
genenames = Take[matrix, {2, genes + 1}, {1}];
arraynames = Take[matrix, {1, 1}, {annotations + 1, arrays + annotations}];
matrix = Take[matrix, {2, genes + 1}, {annotations + 1, arrays + annotations}];
matrix = ToExpression[matrix];
```

(* Calculate SVD *)

```
{eigenarrays, eigenabundances, eigengenes} =
  SingularValueDecomposition[matrix, Min[Dimensions[matrix]]];
eigengenes = Transpose[eigengenes];
eigenabundances = Diagonal[eigenabundances];
list = {1, 12, 13, 15, 16, 18, 20, 21, 22, 23, 26, 27, 28};
Do[{eigengenes[[list[[a]]]] = -eigengenes[[list[[a]]]]}, {a, 1, Dimensions[list][[1]]}]
fractions = eigenabundances^2 / Sum[eigenabundances[[a]]^2, {a, 1, arrays}];
entropy = -N[Sum[fractions[[a]] * Log[fractions[[a]]], {a, 1, arrays}] / Log[arrays]];
entropy = N[Round[100 * entropy] / 100]
```

```
0.69
```

```
(* Create Eigenvectors Raster Display *)

contrast = 3.5;
displaying = Table[
   If[contrast * eigengenes[[i, j]] > 0,
    If[contrast * eigengenes[[i, j]] < 1, {contrast * eigengenes[[i, j]], 0}, {1, 0}],
    If[contrast * eigengenes[[i, j]] > -1, {0, -contrast * eigengenes[[i, j]]}, {0, 1}]],
   {i, 1, arrays}, {j, 1, arrays}];
framex = Table[{a - 0.5, Rotate[arraynames[[1, a]], Pi / 2]}, {a, 1, arrays}];
Do[If[Mod[a + 3, 3] ≠ 0, framex[[a, 2]] = Rotate["       ", Pi / 2]],
 {a, 1, Dimensions[framex][[1]]}]
framey = Table[{a + 1 - 0.5, arrays - a}, {a, 0, arrays - 1}];
Do[If[Mod[a + 3, 3] ≠ 0, framey[[a, 2]] = "    "],
 {a, 1, Dimensions[framey][[1]]}]
labelx = "(f) Arrays";
labely = "Eigenvectors";

g = Graphics[
   Raster[Reverse[Table[Insert[displaying[[i, j]], 0, 3], {i, 1, arrays}, {j, 1, arrays}]]],
   Frame → True,
   FrameTicks → {None, framey, framex, None},
   FrameLabel → {None, labely, labelx, None},
   PlotRange → All, AspectRatio → Full];

(* Fit Eigenvectors with an Asymmetric Parabola *)

k1 = 0.2;
k2 = 0.1;
equi = 11;

Clear[f];
f[x_] := If[(x - equi - 1) < 0, 0.5 * k1 * (x - equi - 1) ^2, 0.5 * k2 * (x - equi - 1) ^2];
inflection = Plot[arrays - f[x + 0.5], {x, 0.5, arrays - 0.5},
   PlotStyle → {RGBColor[0, 0, 1], Thickness[0.013]}];
g13 = Show[{g, inflection},
   PlotRange → All, AspectRatio → 1.05];

(* Fit Eigenvalues with a Geometric Series *)

Clear[constant, λ];
f = FindFit[Table[fractions[[n]], {n, 2, 15}], constant * λ^x, {constant, λ}, x]

{constant → 0.143528, λ → 0.81256}

λ = 0.76;
f = FindFit[Table[fractions[[n]], {n, 2, 15}], constant * λ^x, constant, x]
constant = constant /. f;
correlation = Dot[Table[fractions[[n]], {n, 2, 15}], Table[constant * λ^n, {n, 1, 14}]] /
   Sqrt[Dot[Table[fractions[[n]], {n, 2, 15}], Table[fractions[[n]], {n, 2, 15}]]] /
  Sqrt[Dot[Table[constant * λ^n, {n, 1, 14}], Table[constant * λ^n, {n, 1, 14}]]]

{constant → 0.16946}

0.990239

(* Create Eigenvalues Bar Chart Displays with Fitting Graph *)

fractions[[2]]

0.121753

limit = 0.125;
```

```
Clear[gridx, framex, framey];
gridx = Table[a, {a, 0, limit, N[limit / 5]}];
framex = gridx;
framex = Table[{gridx[[a]], Style[Rotate[framex[[a]], Pi / 2]]}, {a, 1, 6}];
framex[[1]] = {0, Style[Rotate["0     ", Pi / 2]]};
gridx = Table[{gridx[[a]], RGBColor[0, 0, 0]}, {a, 1, 6}];
framey = Table[{a + 1, arrays - a - 15}, {a, 0, 15 - 2}];
Do[If[Mod[a, 2] == 0, framey[[a, 2]] = ""],
 {a, 1, Dimensions[framey][[1]]}]
table = Table[fractions[[arrays - a]], {a, 15, arrays - 2}];
g = BarChart[table,
   BarOrigin → Left,
   PlotRange → {{0, limit}, {0.5, 15 - 1 + 0.5}}, AspectRatio → 1,
   Axes → False,
   Frame → True,
   FrameTicks → {None, framey, framex, None},
   GridLines → {gridx, None},
   ChartStyle → Red];

f1 = NSolve[Log[x / constant] / Log[1 / λ] + 15 == 1, x][[1]]
x1 = x /. f1;
f2 = NSolve[Log[x / constant] / Log[1 / λ] + 15 == 14, x][[1]]
x2 = x /. f2;

{x → 0.00363461}

{x → 0.12879}

graph = Plot[Log[x / constant] / Log[1 / λ] + 15, {x, x1, x2},
   PlotRange → {1, 14},
   PlotStyle → {RGBColor[0, 0, 1], Thickness[0.026]}];
inset = Graphics[Show[{g, graph}, AspectRatio → 1.05]];

fractions[[1]]

0.356542

limit = 0.4;

Clear[gridx, framex, framey];
gridx = Table[a, {a, 0, limit, N[limit / 4]}];
framex = gridx;
framex = Table[{gridx[[a]], Rotate[framex[[a]], Pi / 2]}, {a, 1, 4}];
framex[[1]] = {0, Style[Rotate["0   ", Pi / 2]]};
gridx = Table[{gridx[[a]], RGBColor[0, 0, 0]}, {a, 1, 4}];
framey = Table[{a + 1, arrays - a}, {a, 0, arrays - 1}];
Do[If[Mod[a + 3, 3] ≠ 0, framey[[a, 2]] = ""],
 {a, 1, Dimensions[framey][[1]]}]
labelx = ColumnForm[{
     "(g) Eigenvalue Fraction",
     StringJoin["d = ", ToString[entropy]]}, Center];
g = BarChart[
   Table[fractions[[arrays - a]], {a, 0, arrays - 1}],
   BarOrigin → Left,
   PlotRange → {{0, fractions[[1]]}, {0.5, arrays + 0.25}}, AspectRatio → 1,
   Axes → False,
   Frame → True,
   FrameTicks → {None, framey, framex, None},
   FrameLabel → {None, None, labelx, None},
   GridLines → {gridx, None},
   ChartStyle → Red,
   BarSpacing → Small];

g14 = Graphics[Show[{g,
      Graphics[{RGBColor[1, 1, 0.8], Rectangle[{0.0525, 0.5}, {0.3525, 25}]}],
      Graphics[{Rectangle[{0.0575, 0.75}, {0.3425, 24.75}, inset]}]},
     AspectRatio → 1.2]];
```

```
(* Fit Eigenvectors with a Series of Asymmetric Hermite Functions *)

k1 = 0.2;
k2 = 0.1;
equi = 11;

Clear[g, h];
h[x_, n_, gamma_] := Exp[-gamma * x^2 / 2] * HermiteH[n, Sqrt[gamma] * x] *
   Sqrt[Sqrt[gamma / Pi] / Factorial[n] / (2^n)];
g[x_, n_] := If[x - equi < 0, h[x - equi, n - 1, k1] * (1 / k1)^0.25,
   h[x - equi, n - 1, k2] * (1 / k2)^0.25];
normalization = Table[
   Sqrt[Sum[g[x, n]^2., {x, 0, arrays - 1}]],
   {n, 1, 5}];
correlation = Table[
   Sum[g[x, n] * eigengenes[[n, x + 1]] / normalization[[n]], {x, 0, arrays - 1}],
   {n, 1, 5}];
meancorrelation = Round[100. * Sqrt[Sum[0.2 * correlation[[n]]^2, {n, 1, 5}]]] / 100.
correlation = Round[100. * correlation] / 100.;

0.89

(* Fit Differential Equation with an Asymmetric Parabola *)

Clear[f];
f[x_] := If[(x - equi) < 0, 0.5 * k1 * (x - equi)^2, 0.5 * k2 * (x - equi)^2];

(* Create Selected Eigenvectors Graph Display with Fitting Graphs *)

labelx = "(h) Arrays";
labely = "Relative mRNA Abundance Level";
framex = Table[{a - 1, Rotate[arraynames[[1, a]], Pi / 2]}, {a, 1, arrays}];
Do[If[Mod[a + 3, 3] ≠ 0, framex[[a, 2]] = Rotate["      ", Pi / 2]], {a, 1, Dimensions[framex][[1]]}]
framey = Table[{n - 0.5, n}, {n, 1, 5}];
points = Table[0, {n, 1, 5}];
lines = Table[0, {n, 1, 5}];
Do[{coordinates = Table[{a - 1, eigengenes[[n, a]] + n - 0.5}, {a, 1, arrays}],
  points[[n]] = Table[Point[coordinates[[a]]], {a, 1, arrays}],
  lines[[n]] = Line[coordinates]}, {n, 1, 5}]
points = Table[Graphics[{color[[Mod[n, 5] + 1]], PointSize[0.022], points[[n]]}], {n, 1, 5}];
lines = Table[Graphics[{Thickness[.005], color[[Mod[n, 5] + 1]], lines[[n]]}], {n, 1, 5}];
graphs = Table[Plot[g[x, n] / normalization[[n]] + n - 0.5, {x, 0, arrays - 1},
    PlotStyle → {color[[Mod[n, 5] + 1]], Dashing[{0.03, 0.02}]}, PlotRange → All], {n, 1, 5}];
texts = Table[Graphics[{color[[Mod[n, 5] + 1]], Text[correlation[[n]], {24, n - 0.75}, {-1, 0}]}],
   {n, 1, 5}];
inflection = Plot[f[x], {x, equi - Sqrt[5.45 * 2 / k1], equi + Sqrt[5.45 * 2 / k2]},
   PlotStyle → {RGBColor[0, 0, 0], Dashing[{0.03, 0.02}]},
   Filling → Top, FillingStyle → Opacity[0.1, RGBColor[0, 0, 1]]];

g15 = Show[{points, lines, graphs, texts, inflection},
   Frame → True,
   FrameLabel → {None, labely, labelx, None},
   FrameTicks → {None, framey, framex, None},
   GridLines → {{{equi, RGBColor[0, 0, 0]}},
     Join[{{0, RGBColor[0, 0, 0]}}, Table[{a - 0.5, RGBColor[0, 0, 0]}, {a, 1, 5}]]},
   PlotRange → {-0.05, 5.45}, AspectRatio → 1.05];
```

```
(* SVD of the Transcript Length Data of the Yeast Protein Synthesis Subsets *)

(* Read the Transcript Length Data of the Yeast Translation Subset *)

stream = path <> "Data/Yeast_Transcript_Lengths.txt";
matrix = Import[stream, "Table"];
annotations = Position[matrix[[1]], "100_mm"][[1, 1]] - 1;
{genes, arrays} = Dimensions[matrix] - {1, annotations}
Clear[stream]

{3620, 30}

genenames = Take[matrix, {2, genes + 1}, {1, annotations}];
arraynames = Take[matrix, {1, 1}, {annotations + 1, arrays + annotations}];
annotationnames = Take[matrix, {1, 1}, {1, annotations}];
matrix = Take[matrix, {2, genes + 1}, {annotations + 1, arrays + annotations}];
matrix = ToExpression[matrix];
list = Take[genenames, All, {Position[annotationnames, "Translation_GO:0006412"][[1, 2]]}];
matrix = Take[Sort[Join[list, matrix, 2], OrderedQ[{#2, #1}] &], {1, Count[list, {"Y"}]},
    {2, arrays + 1}];
Dimensions[matrix]

{319, 30}

(* Calculate SVD *)

{eigenarrays, eigenabundances, eigengenes} =
  SingularValueDecomposition[matrix, Min[Dimensions[matrix]]];
eigengenes = Transpose[eigengenes];
eigenabundances = Diagonal[eigenabundances];
list = {1};
Do[{eigengenes[[list[[a]]]] = -eigengenes[[list[[a]]]]}, {a, 1, Dimensions[list][[1]]}]
fractions = eigenabundances^2 / Sum[eigenabundances[[a]]^2, {a, 1, arrays}];
entropy = -N[Sum[fractions[[a]] * Log[fractions[[a]]], {a, 1, arrays}] / Log[arrays]];
entropy = N[Round[100 * entropy] / 100]

0.65

(* Fit Eigenvectors with a Series of Asymmetric Hermite Functions *)

k1 = 0.4;
k2 = 0.2;
equi = 8;

Clear[g, h];
h[x_, n_, gamma_] := Exp[-gamma * x^2 / 2] * HermiteH[n, Sqrt[gamma] * x] *
    Sqrt[Sqrt[gamma / Pi] / Factorial[n] / (2^n)];
g[x_, n_] := If[x - equi < 0, h[x - equi, n - 1, k1] * (1 / k1)^0.25,
    h[x - equi, n - 1, k2] * (1 / k2)^0.25];
normalization = Table[
    Sqrt[Sum[g[x, n]^2., {x, 0, arrays - 1}]],
    {n, 1, 5}];
correlation = Table[
    Sum[g[x, n] * eigengenes[[n, x + 1]] / normalization[[n]], {x, 0, arrays - 1}],
    {n, 1, 5}];
meancorrelation = Round[100. * Sqrt[Sum[0.2 * correlation[[n]]^2, {n, 1, 5}]]] / 100.
correlation = Round[100. * correlation] / 100.;

0.86

(* Fit Differential Equation with an Asymmetric Parabola *)

Clear[f];
f[x_] := If[(x - equi) < 0, 0.5 * k1 * (x - equi)^2, 0.5 * k2 * (x - equi)^2];
```

```
(* Create Selected Eigenvectors Graph Display with Fitting Graphs *)

labelx = "(i) Arrays";
framex = Table[{a - 1, Rotate[arraynames[[1, a]], Pi / 2]}, {a, 1, arrays}];
Do[If[Mod[a + 3, 3] ≠ 0, framex[[a, 2]] = Rotate["       ", Pi / 2]], {a, 1, Dimensions[framex][[1]]}]
framey = Table[{n - 0.5, n}, {n, 1, 5}];
points = Table[0, {n, 1, 5}];
lines = Table[0, {n, 1, 5}];
Do[{coordinates = Table[{a - 1, eigengenes[[n, a]] + n - 0.5}, {a, 1, arrays}],
  points[[n]] = Table[Point[coordinates[[a]]], {a, 1, arrays}],
  lines[[n]] = Line[coordinates]}, {n, 1, 5}]
points = Table[Graphics[{color[[Mod[n, 5] + 1]], PointSize[0.022], points[[n]]}], {n, 1, 5}];
lines = Table[Graphics[{Thickness[.005], color[[Mod[n, 5] + 1]], lines[[n]]}], {n, 1, 5}];
graphs = Table[Plot[g[x, n] / normalization[[n]] + n - 0.5, {x, 0, arrays - 1},
    PlotStyle → {color[[Mod[n, 5] + 1]], Dashing[{0.03, 0.02}]}, PlotRange → All], {n, 1, 5}];
texts = Table[Graphics[{color[[Mod[n, 5] + 1]], Text[correlation[[n]], {24, n - 0.75}, {-1, 0}]}],
   {n, 1, 5}];
inflection = Plot[f[x], {x, equi - Sqrt[5.45 * 2 / k1], equi + Sqrt[5.45 * 2 / k2]},
   PlotStyle → {RGBColor[0, 0, 0], Dashing[{0.03, 0.02}]},
   Filling → Top, FillingStyle → Opacity[0.1, RGBColor[0, 0, 1]]];

g16 = Show[{points, lines, graphs, texts, inflection},
   Frame → True,
   FrameLabel → {None, None, labelx, None},
   FrameTicks → {None, framey, framex, None},
   GridLines → {{{equi, RGBColor[0, 0, 0]}},
     Join[{{0, RGBColor[0, 0, 0]}}, Table[{a - 0.5, RGBColor[0, 0, 0]}, {a, 1, 5}]]},
   PlotRange → {-0.05, 5.45}, AspectRatio → 1.05];

(* Read the Transcript Length Data of the Yeast Ribosome Subset *)

stream = path <> "Data/Yeast_Transcript_Lengths.txt";
matrix = Import[stream, "Table"];
annotations = Position[matrix[[1]], "100_mm"][[1, 1]] - 1;
{genes, arrays} = Dimensions[matrix] - {1, annotations}
Clear[stream]

{3620, 30}

genenames = Take[matrix, {2, genes + 1}, {1, annotations}];
arraynames = Take[matrix, {1, 1}, {annotations + 1, arrays + annotations}];
annotationnames = Take[matrix, {1, 1}, {1, annotations}];
matrix = Take[matrix, {2, genes + 1}, {annotations + 1, arrays + annotations}];
matrix = ToExpression[matrix];
list = Take[genenames, All, {Position[annotationnames, "Ribosome_GO:0005840"][[1, 2]]}];
matrix = Take[Sort[Join[list, matrix, 2], OrderedQ[{#2, #1}] &], {1, Count[list, {"Y"}]},
   {2, arrays + 1}];
Dimensions[matrix]

{274, 30}

(* Calculate SVD *)

{eigenarrays, eigenabundances, eigengenes} =
  SingularValueDecomposition[matrix, Min[Dimensions[matrix]]];
eigengenes = Transpose[eigengenes];
eigenabundances = Diagonal[eigenabundances];
list = {1, 2, 3, 4, 5};
Do[{eigengenes[[list[[a]]]] = -eigengenes[[list[[a]]]]}, {a, 1, Dimensions[list][[1]]}]
fractions = eigenabundances ^ 2 / Sum[eigenabundances[[a]] ^ 2, {a, 1, arrays}];
entropy = -N[Sum[fractions[[a]] * Log[fractions[[a]]], {a, 1, arrays}] / Log[arrays]];
entropy = N[Round[100 * entropy] / 100]

0.6
```

```
(* Fit Eigenvectors with a Series of Asymmetric Hermite Functions *)

k1 = 0.6;
k2 = 0.3;
equi = 8;

Clear[g, h];
h[x_, n_, gamma_] := Exp[-gamma * x^2 / 2] * HermiteH[n, Sqrt[gamma] * x] *
   Sqrt[Sqrt[gamma / Pi] / Factorial[n] / (2^n)];
g[x_, n_] := If[x - equi < 0, h[x - equi, n - 1, k1] * (1 / k1) ^0.25,
   h[x - equi, n - 1, k2] * (1 / k2) ^0.25];
normalization = Table[
   Sqrt[Sum[g[x, n] ^2., {x, 0, arrays - 1}]],
   {n, 1, 5}];
correlation = Table[
   Sum[g[x, n] * eigengenes[[n, x + 1]] / normalization[[n]], {x, 0, arrays - 1}],
   {n, 1, 5}];
meancorrelation = Round[100. * Sqrt[Sum[0.2 * correlation[[n]] ^2, {n, 1, 5}]]] / 100.
correlation = Round[100. * correlation] / 100.;

0.89

(* Fit Differential Equation with an Asymmetric Parabola *)

Clear[f];
f[x_] := If[(x - equi) < 0, 0.5 * k1 * (x - equi) ^2, 0.5 * k2 * (x - equi) ^2];

(* Create Selected Eigenvectors Graph Display with Fitting Graphs *)

labelx = "(j) Arrays";
framex = Table[{a - 1, Rotate[arraynames[[1, a]], Pi / 2]}, {a, 1, arrays}];
Do[If[Mod[a + 3, 3] ≠ 0, framex[[a, 2]] = Rotate["       ", Pi / 2]], {a, 1, Dimensions[framex][[1]]}]
framey = Table[{n - 0.5, n}, {n, 1, 5}];
points = Table[0, {n, 1, 5}];
lines = Table[0, {n, 1, 5}];
Do[{coordinates = Table[{a - 1, eigengenes[[n, a]] + n - 0.5}, {a, 1, arrays}],
  points[[n]] = Table[Point[coordinates[[a]]], {a, 1, arrays}],
  lines[[n]] = Line[coordinates]}, {n, 1, 5}]
points = Table[Graphics[{color[[Mod[n, 5] + 1]], PointSize[0.022], points[[n]]}], {n, 1, 5}];
lines = Table[Graphics[{Thickness[.005], color[[Mod[n, 5] + 1]], lines[[n]]}], {n, 1, 5}];
graphs = Table[Plot[g[x, n] / normalization[[n]] + n - 0.5, {x, 0, arrays - 1},
    PlotStyle → {color[[Mod[n, 5] + 1]], Dashing[{0.03, 0.02}]}, PlotRange → All], {n, 1, 5}];
texts = Table[Graphics[{color[[Mod[n, 5] + 1]], Text[correlation[[n]], {24, n - 0.75}, {-1, 0}]}],
   {n, 1, 5}];
inflection = Plot[f[x], {x, equi - Sqrt[5.45 * 2 / k1], equi + Sqrt[5.45 * 2 / k2]},
   PlotStyle → {RGBColor[0, 0, 0], Dashing[{0.03, 0.02}]},
   Filling → Top, FillingStyle → Opacity[0.1, RGBColor[0, 0, 1]]];

g17 = Show[{points, lines, graphs, texts, inflection},
   Frame → True,
   FrameLabel → {None, None, labelx, ""},
   FrameTicks → {None, framey, framex, None},
   GridLines → {{{equi, RGBColor[0, 0, 0]}},
     Join[{{0, RGBColor[0, 0, 0]}}, Table[{a - 0.5, RGBColor[0, 0, 0]}, {a, 1, 5}]]},
   PlotRange → {-0.05, 5.45}, AspectRatio → 1.05];
```

```
(* Display the SVD of the Human and Yeast Global Sets and Protein Synthesis Subsets *)
```

```
g = GraphicsGrid[{{g8, g9, g10, g11, g12}, {g13, g14, g15, g16, g17}},
   Spacings → {-45, 30}, ImageSize → 1000];

fig2 = Show[{Graphics[{Rectangle[{0, 0}, {1250, 624}, g]}, ImageSize → 1000],
   Graphics[{RGBColor[0, 0, 0], Text[Style["Global Set", FontSize → 12], {390, 650}]}],
   Graphics[{RGBColor[0, 0, 0], Arrowheads[{-0.012, 0.012}], Arrow[{{53, 636}, {747, 636}}]}],
   Graphics[{RGBColor[0, 0, 0], Line[{{53, 630}, {53, 642}}]}],
   Graphics[{RGBColor[0, 0, 0], Line[{{747, 630}, {747, 642}}]}],
   Graphics[{RGBColor[0, 0, 0], Text[Style["Translation Subset", FontSize → 12], {872, 650}]}],
   Graphics[{RGBColor[0, 0, 0], Arrowheads[{-0.012, 0.012}], Arrow[{{765, 636}, {981, 636}}]}],
   Graphics[{RGBColor[0, 0, 0], Line[{{765, 630}, {765, 642}}]}],
   Graphics[{RGBColor[0, 0, 0], Line[{{981, 630}, {981, 642}}]}],
   Graphics[{RGBColor[0, 0, 0], Text[Style["Ribosome Subset", FontSize → 12], {1105, 650}]}],
   Graphics[{RGBColor[0, 0, 0], Arrowheads[{-0.012, 0.012}], Arrow[{{998, 636}, {1213, 636}}]}],
   Graphics[{RGBColor[0, 0, 0], Line[{{998, 630}, {998, 642}}]}],
   Graphics[{RGBColor[0, 0, 0], Line[{{1213, 630}, {1213, 642}}]}],
   Graphics[{RGBColor[0, 0, 0], Text[Style[Rotate["Human", Pi / 2], FontSize → 12], {-12, 436}]}],
   Graphics[{RGBColor[0, 0, 0], Text[Style[Rotate["Yeast", Pi / 2], FontSize → 12], {-12, 116}]}]
  }]
```

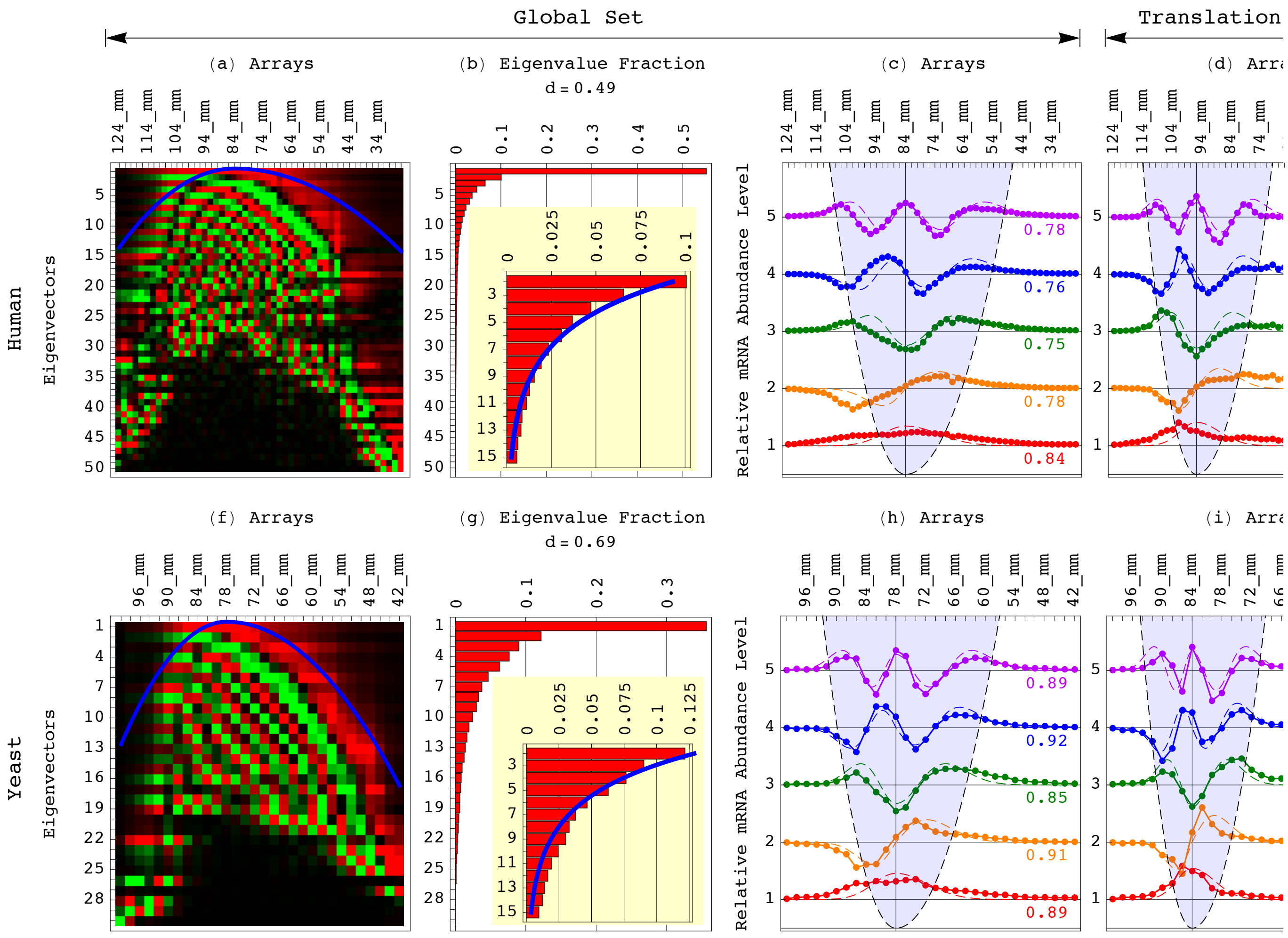


```
Export[path <> "Figures/Figure_2.pdf", fig2, "PDF", ImageSize → 1000, ImageResolution → resolution];
```

(* Fit the Transcript Length Distribution Function of the Human Global Set *)

(* Read the Transcript Length Data of the Human Global Set *)

```
stream = path <> "Data/Human_Transcript_Lengths.txt";
matrix = Import[stream, "Table"];
annotations = Position[matrix[[1]], "124_mm"][[1, 1]] - 1;
{genes, arrays} = Dimensions[matrix] - {1, annotations}
Clear[stream]
```

```
{4109, 50}
```

```
genenames = Take[matrix, {2, genes + 1}, {1, annotations}];
arraynames = Take[matrix, {1, 1}, {annotations + 1, arrays + annotations}];
matrix = Take[matrix, {2, genes + 1}, {annotations + 1, arrays + annotations}];
matrix = ToExpression[matrix];
```

```
{eigenarrays, eigenabundances, eigengenes} =
  SingularValueDecomposition[matrix, Min[Dimensions[matrix]]];
eigenabundances = Diagonal[eigenabundances];
fractions = eigenabundances ^ 2 / Sum[eigenabundances[[a]] ^ 2, {a, 1, arrays}];
Clear[constant, λ];
f = FindFit[Table[fractions[[n]], {n, 2, 15}], constant * λ ^ x, {constant, λ}, x]
```

```
{constant → 0.12368, λ → 0.756294}
```

(* Fit Asymmetric Generalized Coherent State to the Transcript Length Distribution *)

```
k1 = 0.065;
k2 = 0.0325;
equi = 20;
```

```
Clear[a, b, α, β, λ]
λ = 0.76;
f1 = NSolve[Sqrt[(α - k2 / 2) / (α + k2 / 2)] == λ, α];
f2 = FindInstance[{2 * Sqrt[(α /. f1) ^ 2 - β ^ 2] == k2, β > 0}, β];
a = ((α /. f1) + (β /. f2))[[1]]
b = ((α /. f1) - (β /. f2))[[1]] * a / (a - ((α /. f1) - (β /. f2))[[1]])
```

```
0.119167
```

```
0.00225789
```

```
Clear[f, x, p];
f[x_, p_, a_, b_] :=
  If[x < p,
    N[Exp[- (k1 / k2) * a * (x - p) ^ 2]],
    N[Exp[-a * (x - p) ^ 2]]] *
   If[p < 0,
    N[Exp[- (k1 / k2) * b * p ^ 2]],
    N[Exp[-b * p ^ 2]]];
Clear[α, β, λ]
```

(* Fit Peaks of Transcripts' Profiles' with an Asymmetric Gaussian *)

```
distribution = Sum[matrix[[n]], {n, 1, genes}];
distribution = distribution / Sort[distribution, OrderedQ[{#2, #1}] &][[1]];
correlation = Dot[distribution, Table[f[p, p, a, b], {p, -equi, arrays - equi - 1}]] /
    Sqrt[Dot[distribution, distribution]] /
   Sqrt[Dot[Table[f[p, p, a, b], {p, -equi, arrays - equi - 1}],
     Table[f[p, p, a, b], {p, -equi, arrays - equi - 1}]]];
correlation = Round[100 * correlation] / 100.
```

```
0.99
```

```
(* Display the Profiles of Selected Transcripts with Asymmetric Gaussians Fit *)

pfkp = matrix[[Position[genenames, "PFKP"][[1, 1]]]];
cdk4 = matrix[[Position[genenames, "CDK4"][[1, 1]]]];
cox7a2 = matrix[[Position[genenames, "COX7A2"][[1, 1]]]];
c = Sort[Flatten[{pfkp, cdk4, cox7a2}], OrderedQ[{#2, #1}] &][[1]];
{pfkp, cdk4, cox7a2} = {pfkp, cdk4, cox7a2} / c;

pfkpC = Dot[pfkp, Table[f[x, 27, a, b], {x, 1, 50}]] /
   Sqrt[Dot[pfkp, pfkp]] /
  Sqrt[Dot[Table[f[x, 27, a, b], {x, 1, 50}], Table[f[x, 27, a, b], {x, 1, 50}]]];
cdk4C = Dot[cdk4, Table[f[x, 20, a, b], {x, 1, 50}]] /
   Sqrt[Dot[cdk4, cdk4]] /
  Sqrt[Dot[Table[f[x, 20, a, b], {x, 1, 50}], Table[f[x, 20, a, b], {x, 1, 50}]]];
cox7a2C = Dot[cox7a2, Table[f[x, 10, a, b], {x, 1, 50}]] /
    Sqrt[Dot[cox7a2, cox7a2]] /
   Sqrt[Dot[Table[f[x, 10, a, b], {x, 1, 50}], Table[f[x, 10, a, b], {x, 1, 50}]]];
meancorrelation = Round[100 * (pfkpC + cdk4C + cox7a2C) / 3] / 100.
{pfkpC, cdk4C, cox7a2C} = Round[100 * {pfkpC, cdk4C, cox7a2C}] / 100.;

0.87

coordinates = Table[{n, pfkp[[n]]}, {n, 1, arrays}];
points = Table[Point[coordinates[[n]]], {n, 1, arrays}];
pfkpP = Graphics[{RGBColor[1, 0, 0], PointSize[0.022], points}];
pfkpL = Graphics[{RGBColor[1, 0, 0], Line[coordinates]}];
pfkpG = Plot[f[x, 27, a, b] / f[27, 27, a, b] * pfkp[[27]], {x, 1, 50},
   PlotStyle → {RGBColor[1, 0, 0], Dashing[{0.03, 0.02}]},
   Filling → Bottom, FillingStyle → Opacity[0.1, RGBColor[0, 0, 1]],
   Axes → False,
   Frame → True,
   PlotRange → All];

coordinates = Table[{n, cdk4[[n]]}, {n, 1, arrays}];
points = Table[Point[coordinates[[n]]], {n, 1, arrays}];
cdk4P = Graphics[{RGBColor[0, 0, 1], PointSize[0.022], points}];
cdk4L = Graphics[{RGBColor[0, 0, 1], Line[coordinates]}];
cdk4G = Plot[f[x, 20, a, b] / f[20, 20, a, b] * cdk4[[20]], {x, 1, 50},
   PlotStyle → {RGBColor[0, 0, 1], Dashing[{0.03, 0.02}]},
   Filling → Bottom, FillingStyle → Opacity[0.1, RGBColor[0, 0, 1]],
   Axes → False,
   Frame → True,
   PlotRange → All];

coordinates = Table[{n, cox7a2[[n]]}, {n, 1, arrays}];
points = Table[Point[coordinates[[n]]], {n, 1, arrays}];
cox7a2P = Graphics[{RGBColor[0, 0.5, 0], PointSize[0.022], points}];
cox7a2L = Graphics[{RGBColor[0, 0.5, 0], Line[coordinates]}];
cox7a2G = Plot[f[x, 10, a, b] / f[10, 10, a, b] * cox7a2[[10]],
   {x, 1, 50}, PlotStyle → {RGBColor[0, 0.5, 0], Dashing[{0.03, 0.02}]},
   Filling → Bottom, FillingStyle → Opacity[0.1, RGBColor[0, 0, 1]],
   Axes → False,
   Frame → True,
   PlotRange → All];

pfkpT = Graphics[{RGBColor[1, 0, 0], Text[ColumnForm[{"PFKP",
       StringJoin[ReplaceAll[Characters[arraynames[[1, 27]]], "_" → " "]],
       ToString[pfkpC]}, Center], {40, 0.36}]}];
cdk4T = Graphics[{RGBColor[0, 0, 1], Text[ColumnForm[{"CDK4",
       StringJoin[ReplaceAll[Characters[arraynames[[1, 20]]], "_" → " "]],
       ToString[cdk4C]}, Center], {40, 0.61}]}];
cox7a2T = Graphics[{RGBColor[0, 0.5, 0], Text[ColumnForm[{"COX7A2",
       StringJoin[ReplaceAll[Characters[arraynames[[1, 10]]], "_" → " "]],
       ToString[cox7a2C]}, Center], {40, 0.86}]}];
labelx = "(b) Arrays";
labely = "";
framex = Table[{n, Rotate[arraynames[[1, n]], Pi / 2]}, {n, 1, arrays}];
Do[If[Mod[n - 1, 5] ≠ 0, framex[[n, 2]] = Rotate["       ", Pi / 2]],
 {n, 1, Dimensions[framex][[1]]}]
framey = {0.2, 0.4, 0.6, 0.8, 1};
```

```
g = Show[{
    pfkpP, pfkpL, pfkpG, pfkpT,
    cdk4P, cdk4L, cdk4G, cdk4T,
    cox7a2P, cox7a2L, cox7a2G, cox7a2T},
   Frame → True,
   FrameLabel → {None, labely, labelx, None},
   FrameTicks → {None, framey, framex, None},
   GridLines → {{
      {10, {RGBColor[0, 0, 0], Dashing[{0.03, 0.02}]}},
      {20, {RGBColor[0, 0, 0], Dashing[{0.03, 0.02}]}},
      {21, RGBColor[0, 0, 0]},
      {27, {RGBColor[0, 0, 0], Dashing[{0.03, 0.02}]}}}, None}];
g18 = Show[g,
   PlotRange → All, AspectRatio → 1.05];

(* Create Graph Display of the Asymmetric Generalized Coherent State Fit *)

graphs = Table[
   Plot[f[x, p, a, b] / f[0, 0, a, b] * distribution[[equi + 1]],
    {x, -equi, arrays - equi - 1},
    PlotStyle → color[[Mod[p / 5, 5] + 1]],
    Axes → False,
    Frame → True,
    PlotRange → All],
   {p, -equi, arrays - equi - 1, 5}];
graph = Plot[f[p, p, a, b] / f[0, 0, a, b] * distribution[[equi + 1]],
   {p, -equi, arrays - equi - 1},
   PlotStyle → {RGBColor[0, 0, 0], Dashing[{0.03, 0.02}]},
   Filling → Bottom, FillingStyle → Opacity[0.1, RGBColor[0, 0, 1]],
   Axes → False,
   Frame → True,
   PlotRange → {-0.02, 1.02}];

coordinates = Table[{n - equi - 1, distribution[[n]]}, {n, 1, arrays}];
points = Table[Point[coordinates[[n]]], {n, 1, arrays}];
points = Graphics[{RGBColor[0, 0, 0], PointSize[0.022], points}];
lines = Graphics[{RGBColor[0, 0, 0], Line[coordinates]}];

distributionT = Graphics[{Text[ColumnForm[{"Human Global",
       StringJoin[ReplaceAll[Characters[arraynames[[1, equi + 1]]], "_" → " "]],
       ToString[correlation]}, Center], {19, 0.86}]}];
labelx = "(a) Arrays";
labely = "Relative mRNA Abundance Level";
framex = Table[{n - equi - 1, Rotate[arraynames[[1, n]], Pi / 2]}, {n, 1, arrays}];
Do[If[Mod[n - 1, 5] ≠ 0, framex[[n, 2]] = Rotate["       ", Pi / 2],
   framex[[n, 2]] = StyleForm[framex[[n, 2]], FontColor → color[[Mod[(n + 4) / 5, 5] + 1]]]],
  {n, 1, Dimensions[framex][[1]]}];
framey = {0.2, 0.4, 0.6, 0.8, 1};
g = Show[{graphs, graph, points, lines, distributionT},
   Frame → True,
   FrameLabel → {None, labely, labelx, None},
   FrameTicks → {None, framey, framex, None},
   GridLines → {{{0, RGBColor[0, 0, 0]}}, None}];
g19 = Show[g,
   PlotRange → {-0.02, 1.02}, AspectRatio → 1.05];
Clear[a, b];
```

```
(* Fit the Transcript Length Distribution Function of the Yeast Global Set *)

(* Read the Transcript Length Data of the Yeast Global Set *)

stream = path <> "Data/Yeast_Transcript_Lengths.txt";
matrix = Import[stream, "Table"];
annotations = Position[matrix[[1]], "100_mm"][[1, 1]] - 1;
{genes, arrays} = Dimensions[matrix] - {1, annotations}
Clear[stream]

{3620, 30}

genenames = Take[matrix, {2, genes + 1}, {1, annotations}];
arraynames = Take[matrix, {1, 1}, {annotations + 1, arrays + annotations}];
matrix = Take[matrix, {2, genes + 1}, {annotations + 1, arrays + annotations}];
matrix = ToExpression[matrix];

{eigenarrays, eigenabundances, eigengenes} =
  SingularValueDecomposition[matrix, Min[Dimensions[matrix]]];
eigenabundances = Diagonal[eigenabundances];
fractions = eigenabundances^2 / Sum[eigenabundances[[a]]^2, {a, 1, arrays}];
Clear[constant, λ];
f = FindFit[Table[fractions[[n]], {n, 2, 15}], constant * λ^x, {constant, λ}, x]

{constant → 0.143528, λ → 0.81256}

(* Fit Asymmetric Generalized Coherent State to the Transcript Length Distribution *)

k1 = 0.2;
k2 = 0.1;
equi = 11;

Clear[a, b, α, β, λ]
λ = 0.76;
f1 = NSolve[Sqrt[(α - k2 / 2) / (α + k2 / 2)] == λ, α];
f2 = FindInstance[{2 * Sqrt[(α /. f1)^2 - β^2] == k2, β > 0}, β];
a = ((α /. f1) + (β /. f2))[[1]]
b = ((α /. f1) - (β /. f2))[[1]] * a / (a - ((α /. f1) - (β /. f2))[[1]])

0.366667

0.00694737

Clear[f, x, p];
f[x_, p_, a_, b_] :=
  If[x < p,
    N[Exp[-(k1 / k2) * a * (x - p)^2]],
    N[Exp[-a * (x - p)^2]]] *
   If[p < 0,
    N[Exp[-(k1 / k2) * b * p^2]],
    N[Exp[-b * p^2]]];
Clear[α, β, λ]

(* Fit Peaks of Transcripts' Profiles' with an Asymmetric Gaussian *)

distribution = Sum[matrix[[n]], {n, 1, genes}];
distribution = distribution / Sort[distribution, OrderedQ[{#2, #1}] &][[1]];
correlation = Dot[distribution, Table[f[p, p, a, b], {p, -equi, arrays - equi - 1}]] /
    Sqrt[Dot[distribution, distribution]] /
   Sqrt[Dot[Table[f[p, p, a, b], {p, -equi, arrays - equi - 1}],
     Table[f[p, p, a, b], {p, -equi, arrays - equi - 1}]]];
correlation = Round[100 * correlation] / 100.

0.99
```

```
(* Display the Profiles of Selected Transcripts with Asymmetric Gaussians Fit *)

ymr205c = matrix[[Position[genenames, "YMR205C"][[1, 1]]]];
ybr160w = matrix[[Position[genenames, "YBR160W"][[1, 1]]]];
ydl067c = matrix[[Position[genenames, "YDL067C"][[1, 1]]]];
c = Sort[Flatten[{ymr205c, ybr160w, ydl067c}], OrderedQ[{#2, #1}] &][[1]];
{ymr205c, ybr160w, ydl067c} = {ymr205c, ybr160w, ydl067c} / c;

ymr205cC = Dot[ymr205c, Table[f[x, 25, a, b], {x, 1, 30}]] /
     Sqrt[Dot[ymr205c, ymr205c]] /
   Sqrt[Dot[Table[f[x, 25, a, b], {x, 1, 30}], Table[f[x, 25, a, b], {x, 1, 30}]]];
ybr160wC = Dot[ybr160w, Table[f[x, 14, a, b], {x, 1, 30}]] /
     Sqrt[Dot[ybr160w, ybr160w]] /
   Sqrt[Dot[Table[f[x, 14, a, b], {x, 1, 30}], Table[f[x, 14, a, b], {x, 1, 30}]]];
ydl067cC = Dot[ydl067c, Table[f[x, 6, a, b], {x, 1, 30}]] /
     Sqrt[Dot[ydl067c, ydl067c]] /
   Sqrt[Dot[Table[f[x, 6, a, b], {x, 1, 30}], Table[f[x, 6, a, b], {x, 1, 30}]]];
meancorrelation = Round[100 * (ymr205cC + ybr160wC + ydl067cC) / 3] / 100.
{ymr205cC, ybr160wC, ydl067cC} = Round[100 * {ymr205cC, ybr160wC, ydl067cC}] / 100.;

0.89

coordinates = Table[{n, ymr205c[[n]]}, {n, 1, arrays}];
points = Table[Point[coordinates[[n]]], {n, 1, arrays}];
ymr205cP = Graphics[{RGBColor[1, 0, 0], PointSize[0.022], points}];
ymr205cL = Graphics[{RGBColor[1, 0, 0], Line[coordinates]}];
ymr205cG = Plot[f[x, 25, a, b] / f[25, 25, a, b] * ymr205c[[25]], {x, 1, 30},
   PlotStyle → {RGBColor[1, 0, 0], Dashing[{0.03, 0.02}]},
   Filling → Bottom, FillingStyle → Opacity[0.1, RGBColor[0, 0, 1]],
   Axes → False,
   Frame → True,
   PlotRange → All];

coordinates = Table[{n, ybr160w[[n]]}, {n, 1, arrays}];
points = Table[Point[coordinates[[n]]], {n, 1, arrays}];
ybr160wP = Graphics[{RGBColor[0, 0, 1], PointSize[0.022], points}];
ybr160wL = Graphics[{RGBColor[0, 0, 1], Line[coordinates]}];
ybr160wG = Plot[f[x, 14, a, b] / f[14, 14, a, b] * ybr160w[[14]], {x, 1, 30},
   PlotStyle → {RGBColor[0, 0, 1], Dashing[{0.03, 0.02}]},
   Filling → Bottom, FillingStyle → Opacity[0.1, RGBColor[0, 0, 1]],
   Axes → False,
   Frame → True,
   PlotRange → All];

coordinates = Table[{n, ydl067c[[n]]}, {n, 1, arrays}];
points = Table[Point[coordinates[[n]]], {n, 1, arrays}];
ydl067cP = Graphics[{RGBColor[0, 0.5, 0], PointSize[0.022], points}];
ydl067cL = Graphics[{RGBColor[0, 0.5, 0], Line[coordinates]}];
ydl067cG = Plot[f[x, 6, a, b] / f[6, 6, a, b] * ydl067c[[6]], {x, 1, 30},
   PlotStyle → {RGBColor[0, 0.5, 0], Dashing[{0.03, 0.02}]},
   Filling → Bottom, FillingStyle → Opacity[0.1, RGBColor[0, 0, 1]],
   Axes → False,
   Frame → True,
   PlotRange → All];

ymr205cT = Graphics[{RGBColor[1, 0, 0], Text[ColumnForm[{"PFK2",
       StringJoin[ReplaceAll[Characters[arraynames[[1, 25]]], "_" → " "]],
       ToString[ymr205cC]}, Center], {20, 0.36}]}];
ybr160wT = Graphics[{RGBColor[0, 0, 1], Text[ColumnForm[{"CDC28",
       StringJoin[ReplaceAll[Characters[arraynames[[1, 14]]], "_" → " "]],
       ToString[ybr160wC]}, Center], {20, 0.61}]}];
ydl067cT = Graphics[{RGBColor[0, 0.5, 0], Text[ColumnForm[{"COX9",
       StringJoin[ReplaceAll[Characters[arraynames[[1, 6]]], "_" → " "]],
       ToString[ydl067cC]}, Center], {20, 0.86}]}];
labelx = "(d) Arrays";
labely = "";
framex = Table[{n, Rotate[arraynames[[1, n]], Pi / 2]}, {n, 1, arrays}];
Do[If[Mod[n, 3] ≠ 0, framex[[n, 2]] = Rotate["       ", Pi / 2]],
 {n, 1, Dimensions[framex][[1]]}]
framey = {0.2, 0.4, 0.6, 0.8, 1};
```

```
g = Show[{
    ymr205cP, ymr205cL, ymr205cG, ymr205cT,
    ybr160wP, ybr160wL, ybr160wG, ybr160wT,
    ydl067cP, ydl067cL, ydl067cG, ydl067cT},
   Frame → True,
   FrameLabel → {None, labely, labelx, None},
   FrameTicks → {None, framey, framex, None},
   GridLines → {{
      {6, {RGBColor[0, 0, 0], Dashing[{0.03, 0.02}]}},
      {12, RGBColor[0, 0, 0]},
      {14, {RGBColor[0, 0, 0], Dashing[{0.03, 0.02}]}},
      {25, {RGBColor[0, 0, 0], Dashing[{0.03, 0.02}]}}}, None}];
g20 = Show[g,
   PlotRange → All, AspectRatio → 1.05];

(* Create Graph Display of the Asymmetric Generalized Coherent State Fit *)

graphs = Table[
   Plot[f[x, p, a, b] / f[0, 0, a, b] * distribution[[equi + 1]],
    {x, - equi, arrays - equi - 1},
    PlotStyle → color[[Mod[(p - 3) / 3, 5] + 1]],
    Axes → False,
    Frame → True,
    PlotRange → All],
   {p, - equi + 2, arrays - equi - 1, 3}];
graph = Plot[f[p, p, a, b] / f[0, 0, a, b] * distribution[[equi + 1]],
   {p, - equi, arrays - equi - 1},
   PlotStyle → {RGBColor[0, 0, 0], Dashing[{0.03, 0.02}]},
   Filling → Bottom, FillingStyle → Opacity[0.1, RGBColor[0, 0, 1]],
   Axes → False,
   Frame → True,
   PlotRange → {-0.02, 1.02}];

coordinates = Table[{n - equi - 1, distribution[[n]]}, {n, 1, arrays}];
points = Table[Point[coordinates[[n]]], {n, 1, arrays}];
points = Graphics[{RGBColor[0, 0, 0], PointSize[0.022], points}];
lines = Graphics[{RGBColor[0, 0, 0], Line[coordinates]}];

distributionT = Graphics[{Text[ColumnForm[{"Yeast Global",
       StringJoin[ReplaceAll[Characters[arraynames[[1, equi + 1]]], "_" → " "]],
       ToString[correlation]}, Center], {12, 0.86}]}];
labelx = "(c) Arrays";
labely = "Relative mRNA Abundance Level";
framex = Table[{n - equi - 1, Rotate[arraynames[[1, n]], Pi / 2]}, {n, 1, arrays}];
Do[If[Mod[n + 3, 3] ≠ 0, framex[[n, 2]] = Rotate["       ", Pi / 2],
   framex[[n, 2]] = StyleForm[framex[[n, 2]], FontColor → color[[Mod[n / 3, 5] + 1]]]],
  {n, 1, Dimensions[framex][[1]]}];
framey = {0.2, 0.4, 0.6, 0.8, 1};
g = Show[{graphs, graph, points, lines, distributionT},
   Frame → True,
   FrameLabel → {None, labely, labelx, None},
   FrameTicks → {None, framey, framex, None},
   GridLines → {{{0, RGBColor[0, 0, 0]}}, None}];
g21 = Show[g,
   PlotRange → {-0.02, 1.02}, AspectRatio → 1.05];
Clear[a, b];
```

```
(* Display the Transcript Length Distribution Functions of the Human and Yeast Sets *)
```

```
g = GraphicsGrid[{{g19, g18}, {g21, g20}}, Spacings → {-36, 16}, ImageSize → 500];

fig3 = Show[{Graphics[{Rectangle[{0, 0}, {625, 705}, g]}, ImageSize → 500],
   Graphics[{Text[Style["Global Set", FontSize → 12], {188, 720}]}],
   Graphics[{Text[Style["Selected Transcripts", FontSize → 12], {484, 720}]}],
   Graphics[{Text[Style[Rotate["Human", Pi / 2], FontSize → 12], {-6, 496}]}],
   Graphics[{Text[Style[Rotate["Yeast", Pi / 2], FontSize → 12], {-6, 137}]}]
  }]
```

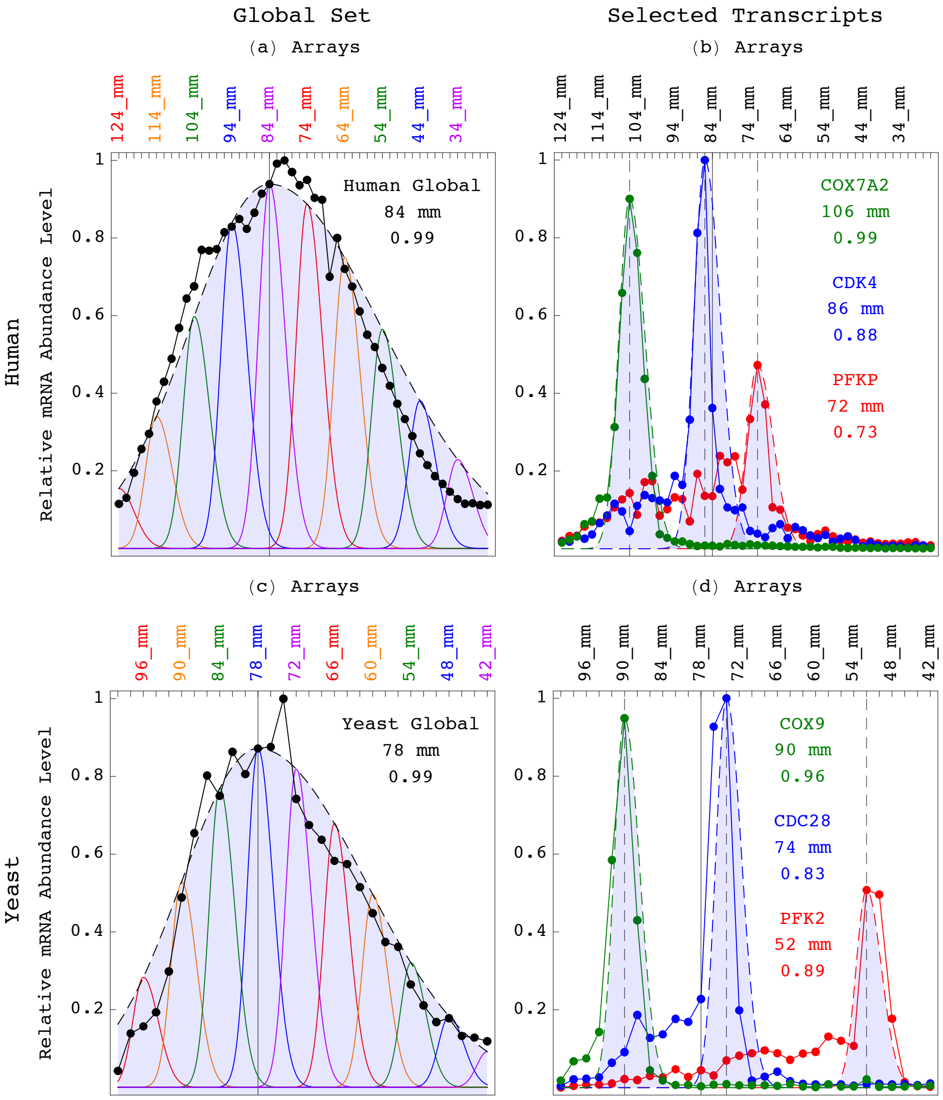


```
Export[path <> "Figures/Figure_3.pdf", fig3, "PDF", ImageSize → 500, ImageResolution → resolution];
```

```
(* SVD of the Human Transcripts Overexpressed in the Normal Brain or GBM Tumor *)
```

```
(* Read the Length Data of the Human Transcripts Overexpressed in the Normal Brain *)

stream = path <> "Data/Human_Transcript_Lengths.txt";
matrix = Import[stream, "Table"];
annotations = Position[matrix[[1]], "124_mm"][[1, 1]] - 1;
{genes, arrays} = Dimensions[matrix] - {1, annotations}
Clear[stream]

{4109, 50}

genenames = Take[matrix, {2, genes + 1}, {1, annotations}];
arraynames = Take[matrix, {1, 1}, {annotations + 1, arrays + annotations}];
annotationnames = Take[matrix, {1, 1}, {1, annotations}];
matrix = Take[matrix, {2, genes + 1}, {annotations + 1, arrays + annotations}];
matrix = ToExpression[matrix];
list = Take[genenames, All, {Position[annotationnames,
       "250_Normal-Tumor"][[1, 2]]}];
matrix = Take[Sort[Join[list, matrix, 2], OrderedQ[{#2, #1}] &], {1, Count[list, {"Y"}]},
    {2, arrays + 1}];
Dimensions[matrix]

{102, 50}
```

```
(* Calculate SVD *)

{eigenarrays, eigenabundances, eigengenes} =
  SingularValueDecomposition[matrix, Min[Dimensions[matrix]]];
eigengenes = Transpose[eigengenes];
eigenabundances = Diagonal[eigenabundances];
list = {1, 3, 5};
Do[{eigengenes[[list[[a]]]] = -eigengenes[[list[[a]]]]}, {a, 1, Dimensions[list][[1]]}]
```

```
(* Fit Eigenvectors with a Series of Asymmetric Hermite Functions *)

k1 = 0.0325;
k2 = 0.0325;
equi = 22;

Clear[g, h];
h[x_, n_, gamma_] := Exp[-gamma * x ^ 2 / 2] * HermiteH[n, Sqrt[gamma] * x] *
    Sqrt[Sqrt[gamma / Pi] / Factorial[n] / (2 ^ n)];
g[x_, n_] := If[x - equi < 0, h[x - equi, n - 1, k1] * (1 / k1) ^ 0.25,
    h[x - equi, n - 1, k2] * (1 / k2) ^ 0.25];
normalization = Table[
    Sqrt[Sum[g[x, n] ^ 2., {x, 0, arrays - 1}]],
    {n, 1, 5}];
correlation = Table[
    Sum[g[x, n] * eigengenes[[n, x + 1]] / normalization[[n]], {x, 0, arrays - 1}],
    {n, 1, 5}];
meancorrelation = Round[100. * Sqrt[Sum[0.2 * correlation[[n]] ^ 2, {n, 1, 5}]]] / 100.
correlation = Round[100. * correlation] / 100.;

0.86
```

```
(* Fit Differential Equation with an Asymmetric Parabola *)

Clear[f];
f[x_] := If[(x - equi) < 0, 0.5 * k1 * (x - equi) ^ 2, 0.5 * k2 * (x - equi) ^ 2];
```

```
(* Create Selected Eigenvectors Graph Display with Fitting Graphs *)

labelx = "(a) Arrays";
labely = "Relative mRNA Abundance Level";
framex = Table[{a - 1, Rotate[arraynames[[1, a]], Pi / 2]}, {a, 1, arrays}];
Do[If[Mod[a - 1, 5] ≠ 0, framex[[a, 2]] = Rotate["       ", Pi / 2]], {a, 1, Dimensions[framex][[1]]}]
framey = Table[{n - 0.5, "   " <> ToString[n]}, {n, 1, 5}];
points = Table[0, {n, 1, 5}];
lines = Table[0, {n, 1, 5}];
Do[{coordinates = Table[{a - 1, eigengenes[[n, a]] + n - 0.5}, {a, 1, arrays}],
  points[[n]] = Table[Point[coordinates[[a]]], {a, 1, arrays}],
  lines[[n]] = Line[coordinates]}, {n, 1, 5}]
points = Table[Graphics[{color[[Mod[n, 5] + 1]], PointSize[0.022], points[[n]]}], {n, 1, 5}];
lines = Table[Graphics[{Thickness[.005], color[[Mod[n, 5] + 1]], lines[[n]]}], {n, 1, 5}];
graphs = Table[Plot[g[x, n] / normalization[[n]] + n - 0.5, {x, 0, arrays - 1},
     PlotStyle → {color[[Mod[n, 5] + 1]], Dashing[{0.03, 0.02}]}, PlotRange → All], {n, 1, 5}];
texts = Table[Graphics[{color[[Mod[n, 5] + 1]], Text[correlation[[n]], {40, n - 0.75}, {-1, 0}]}],
    {n, 1, 5}];
inflection = Plot[f[x], {x, equi - Sqrt[5.45 * 2 / k1], equi + Sqrt[5.45 * 2 / k2]},
    PlotStyle → {RGBColor[0, 0, 0], Dashing[{0.03, 0.02}]},
    Filling → Top, FillingStyle → Opacity[0.1, RGBColor[0, 0, 1]]];

inflectionR = Plot[f[x], {x, equi - Sqrt[5.45 * 2 / k1], equi + Sqrt[5.45 * 2 / k2]},
    PlotStyle → {RGBColor[1, 0, 0], Thickness[0.006]},
    PlotRange → {-0.05, 5.45},
    Filling → Top, FillingStyle → Opacity[0.1, RGBColor[0, 0, 1]]];

g22 = Show[{points, lines, graphs, texts, inflection},
    Frame → True,
    FrameLabel → {None, labely, labelx, None},
    FrameTicks → {None, framey, framex, None},
    GridLines → {{{equi, RGBColor[0, 0, 0]}},
      Join[{{0, RGBColor[0, 0, 0]}}, Table[{a - 0.5, RGBColor[0, 0, 0]}, {a, 1, 5}]]},
    PlotRange → {-0.05, 5.45}, AspectRatio → 1.05];

(* Read the Length Data of the Human Transcripts Overexpressed in the GBM Tumor *)

stream = path <> "Data/Human_Transcript_Lengths.txt";
matrix = Import[stream, "Table"];
annotations = Position[matrix[[1]], "124_mm"][[1, 1]] - 1;
{genes, arrays} = Dimensions[matrix] - {1, annotations}
Clear[stream]

{4109, 50}

genenames = Take[matrix, {2, genes + 1}, {1, annotations}];
arraynames = Take[matrix, {1, 1}, {annotations + 1, arrays + annotations}];
annotationnames = Take[matrix, {1, 1}, {1, annotations}];
matrix = Take[matrix, {2, genes + 1}, {annotations + 1, arrays + annotations}];
matrix = ToExpression[matrix];
list = Take[genenames, All, {Position[annotationnames,
       "250_Tumor-Normal"][[1, 2]]}];
matrix = Take[Sort[Join[list, matrix, 2], OrderedQ[{#2, #1}] &], {1, Count[list, {"Y"}]},
    {2, arrays + 1}];
Dimensions[matrix]

{135, 50}

(* Calculate SVD *)

{eigenarrays, eigenabundances, eigengenes} =
  SingularValueDecomposition[matrix, Min[Dimensions[matrix]]];
eigengenes = Transpose[eigengenes];
eigenabundances = Diagonal[eigenabundances];
list = {1, 3};
Do[{eigengenes[[list[[a]]]] = -eigengenes[[list[[a]]]]}, {a, 1, Dimensions[list][[1]]}]
```

```
(* Fit Eigenvectors with a Series of Asymmetric Hermite Functions *)

k1 = 0.065;
k2 = 0.0325;
equi = 17;

Clear[g, h];
h[x_, n_, gamma_] := Exp[-gamma * x^2 / 2] * HermiteH[n, Sqrt[gamma] * x] *
   Sqrt[Sqrt[gamma / Pi] / Factorial[n] / (2^n)];
g[x_, n_] := If[x - equi < 0, h[x - equi, n - 1, k1] * (1 / k1)^0.25,
   h[x - equi, n - 1, k2] * (1 / k2)^0.25];
normalization = Table[
   Sqrt[Sum[g[x, n]^2., {x, 0, arrays - 1}]],
   {n, 1, 5}];
correlation = Table[
   Sum[g[x, n] * eigengenes[[n, x + 1]] / normalization[[n]], {x, 0, arrays - 1}],
   {n, 1, 5}];
meancorrelation = Round[100. * Sqrt[Sum[0.2 * correlation[[n]]^2, {n, 1, 5}]]] / 100.
correlation = Round[100. * correlation] / 100.;

0.82

(* Fit Differential Equation with an Asymmetric Parabola *)

Clear[f];
f[x_] := If[(x - equi) < 0, 0.5 * k1 * (x - equi)^2, 0.5 * k2 * (x - equi)^2];

(* Create Selected Eigenvectors Graph Display with Fitting Graphs *)

labelx = "(b) Arrays";
framex = Table[{a - 1, Rotate[arraynames[[1, a]], Pi / 2]}, {a, 1, arrays}];
Do[If[Mod[a - 1, 5] ≠ 0, framex[[a, 2]] = Rotate["       ", Pi / 2]], {a, 1, Dimensions[framex][[1]]}]
framey = Table[{n - 0.5, "   " <> ToString[n]}, {n, 1, 5}];
points = Table[0, {n, 1, 5}];
lines = Table[0, {n, 1, 5}];

Do[{coordinates = Table[{a - 1, eigengenes[[n, a]] + n - 0.5}, {a, 1, arrays}],
  points[[n]] = Table[Point[coordinates[[a]]], {a, 1, arrays}],
  lines[[n]] = Line[coordinates]}, {n, 1, 5}]
points = Table[Graphics[{color[[Mod[n, 5] + 1]], PointSize[0.022], points[[n]]}], {n, 1, 5}];
lines = Table[Graphics[{Thickness[.005], color[[Mod[n, 5] + 1]], lines[[n]]}], {n, 1, 5}];
graphs = Table[Plot[g[x, n] / normalization[[n]] + n - 0.5, {x, 0, arrays - 1},
    PlotStyle → {color[[Mod[n, 5] + 1]], Dashing[{0.03, 0.02}]}, PlotRange → All], {n, 1, 5}];
texts = Table[Graphics[{color[[Mod[n, 5] + 1]], Text[correlation[[n]], {40, n - 0.75}, {-1, 0}]}],
   {n, 1, 5}];
inflection = Plot[f[x], {x, equi - Sqrt[5.45 * 2 / k1], equi + Sqrt[5.45 * 2 / k2]},
   PlotStyle → {RGBColor[0, 0, 0], Dashing[{0.03, 0.02}]},
   Filling → Top, FillingStyle → Opacity[0.1, RGBColor[0, 0, 1]]];

inflectionB = Plot[f[x], {x, equi - Sqrt[5.45 * 2 / k1], equi + Sqrt[5.45 * 2 / k2]},
   PlotStyle → {RGBColor[0, 0, 1], Thickness[0.006]},
   PlotRange → {-0.05, 5.45},
   Filling → Top, FillingStyle → Opacity[0.1, RGBColor[0, 0, 1]]];

g23 = Show[{points, lines, graphs, texts, inflection},
   Frame → True,
   FrameLabel → {None, None, labelx, None},
   FrameTicks → {None, framey, framex, None},
   GridLines → {{{equi, RGBColor[0, 0, 0]}},
     Join[{{0, RGBColor[0, 0, 0]}}, Table[{a - 0.5, RGBColor[0, 0, 0]}, {a, 1, 5}]]},
   PlotRange → {-0.05, 5.45}, AspectRatio → 1.05];
```

```
(* Read the Length Data of the Transcripts Overexpressed in Both Normal and Tumor *)

stream = path <> "Data/Human_Transcript_Lengths.txt";
matrix = Import[stream, "Table"];
annotations = Position[matrix[[1]], "124_mm"][[1, 1]] - 1;
{genes, arrays} = Dimensions[matrix] - {1, annotations}
Clear[stream]

{4109, 50}

genenames = Take[matrix, {2, genes + 1}, {1, annotations}];
arraynames = Take[matrix, {1, 1}, {annotations + 1, arrays + annotations}];
annotationnames = Take[matrix, {1, 1}, {1, annotations}];
matrix = Take[matrix, {2, genes + 1}, {annotations + 1, arrays + annotations}];
matrix = ToExpression[matrix];
list = Take[genenames, All, {Position[annotationnames,
       "250_Normal+Tumor"][[1, 2]]}];
matrix = Take[Sort[Join[list, matrix, 2], OrderedQ[{#2, #1}] &], {1, Count[list, {"Y"}]},
    {2, arrays + 1}];
Dimensions[matrix]

{200, 50}

(* Calculate SVD *)

{eigenarrays, eigenabundances, eigengenes} =
  SingularValueDecomposition[matrix, Min[Dimensions[matrix]]];
eigengenes = Transpose[eigengenes];
eigenabundances = Diagonal[eigenabundances];
list = {1, 2, 3, 5};
Do[{eigengenes[[list[[a]]]] = -eigengenes[[list[[a]]]]}, {a, 1, Dimensions[list][[1]]}]

(* Fit Eigenvectors with a Series of Asymmetric Hermite Functions *)

k1 = 0.13;
k2 = 0.0325;
equi = 14;

Clear[g, h];
h[x_, n_, gamma_] := Exp[-gamma * x^2 / 2] * HermiteH[n, Sqrt[gamma] * x] *
    Sqrt[Sqrt[gamma / Pi] / Factorial[n] / (2^n)];
g[x_, n_] := If[x - equi < 0, h[x - equi, n - 1, k1] * (1 / k1)^0.25,
    h[x - equi, n - 1, k2] * (1 / k2)^0.25];
normalization = Table[
    Sqrt[Sum[g[x, n]^2., {x, 0, arrays - 1}]],
    {n, 1, 5}];
correlation = Table[
    Sum[g[x, n] * eigengenes[[n, x + 1]] / normalization[[n]], {x, 0, arrays - 1}],
    {n, 1, 5}];
meancorrelation = Round[100. * Sqrt[Sum[0.2 * correlation[[n]]^2, {n, 1, 5}]]] / 100.
correlation = Round[100. * correlation] / 100.;

0.88

(* Fit Differential Equation with an Asymmetric Parabola *)

Clear[f];
f[x_] := If[(x - equi) < 0, 0.5 * k1 * (x - equi)^2, 0.5 * k2 * (x - equi)^2];
```

```
(* Create Selected Eigenvectors Graph Display with Fitting Graphs *)

labelx = "(c) Arrays";
framex = Table[{a - 1, Rotate[arraynames[[1, a]], Pi / 2]}, {a, 1, arrays}];
Do[If[Mod[a - 1, 5] ≠ 0, framex[[a, 2]] = Rotate["       ", Pi / 2]], {a, 1, Dimensions[framex][[1]]}]
framey = Table[{n - 0.5, "   " <> ToString[n]}, {n, 1, 5}];
points = Table[0, {n, 1, 5}];
lines = Table[0, {n, 1, 5}];
Do[{coordinates = Table[{a - 1, eigengenes[[n, a]] + n - 0.5}, {a, 1, arrays}],
  points[[n]] = Table[Point[coordinates[[a]]], {a, 1, arrays}],
  lines[[n]] = Line[coordinates]}, {n, 1, 5}]
points = Table[Graphics[{color[[Mod[n, 5] + 1]], PointSize[0.022], points[[n]]}], {n, 1, 5}];
lines = Table[Graphics[{Thickness[.005], color[[Mod[n, 5] + 1]], lines[[n]]}], {n, 1, 5}];
graphs = Table[Plot[g[x, n] / normalization[[n]] + n - 0.5, {x, 0, arrays - 1},
    PlotStyle → {color[[Mod[n, 5] + 1]], Dashing[{0.03, 0.02}]}, PlotRange → All], {n, 1, 5}];
texts = Table[Graphics[{color[[Mod[n, 5] + 1]], Text[correlation[[n]], {40, n - 0.75}, {-1, 0}]}],
   {n, 1, 5}];
inflection = Plot[f[x], {x, equi - Sqrt[5.45 * 2 / k1], equi + Sqrt[5.45 * 2 / k2]},
   PlotStyle → {RGBColor[0, 0, 0], Dashing[{0.03, 0.02}]},
   Filling → Top, FillingStyle → Opacity[0.1, RGBColor[0, 0, 1]]];

inflectionG = Plot[f[x], {x, equi - Sqrt[5.45 * 2 / k1], equi + Sqrt[5.45 * 2 / k2]},
   PlotStyle → {RGBColor[0, 0.5, 0], Thickness[0.006]},
   PlotRange → {-0.05, 5.45},
   Filling → Top, FillingStyle → Opacity[0.1, RGBColor[0, 0, 1]]];

g24 = Show[{points, lines, graphs, texts, inflection},
   Frame → True,
   FrameLabel → {None, None, labelx, ""},
   FrameTicks → {None, framey, framex, None},
   GridLines → {{{equi, RGBColor[0, 0, 0]}},
     Join[{{0, RGBColor[0, 0, 0]}}, Table[{a - 0.5, RGBColor[0, 0, 0]}, {a, 1, 5}]]},
   PlotRange → {-0.05, 5.45}, AspectRatio → 1.05];

(* Create Display of the Asymmetric Parabolas that Fit the Subsets' Distributions *)

labelx = "(d) Arrays";
framex = Table[{a - 1, Rotate[arraynames[[1, a]], Pi / 2]}, {a, 1, arrays}];
Do[If[Mod[a - 1, 5] ≠ 0, framex[[a, 2]] = Rotate["       ", Pi / 2]], {a, 1, Dimensions[framex][[1]]}];
framey = Table[{n - 0.5, "   " <> ToString[n]}, {n, 1, 5}];
g25 = Show[{inflectionG, inflectionB, inflectionR},
   Axes → False,
   Frame → True,
   FrameLabel → {None, None, labelx, ColumnForm[{"", ""}, Center]},
   FrameTicks → {None, framey, framex, None},
   GridLines → {{
       {14, {RGBColor[0, 0.5, 0], Thickness[0.004]}},
       {17, {RGBColor[0, 0, 1], Thickness[0.004]}},
       {22, {RGBColor[1, 0, 0], Thickness[0.004]}}},
     {{0, RGBColor[0, 0, 0]}}},
   PlotRange → {{0, 49}, {-0.05, 5.45}}, AspectRatio → 1.05];
```

```
(* Fit the Length Distribution Functions of the Subsets of Human Transcripts *)

(* Read the Length Data of the Subsets of Overexpressed Human Transcripts *)

stream = path <> "Data/Human_Transcript_Lengths.txt";
matrix = Import[stream, "Table"];
annotations = Position[matrix[[1]], "124_mm"][[1, 1]] - 1;
{genes, arrays} = Dimensions[matrix] - {1, annotations}
Clear[stream]

{4109, 50}

genenames = Take[matrix, {2, genes + 1}, {1, annotations}];
arraynames = Take[matrix, {1, 1}, {annotations + 1, arrays + annotations}];
annotationnames = Take[matrix, {1, 1}, {1, annotations}];
matrix = Take[matrix, {2, genes + 1}, {annotations + 1, arrays + annotations}];
matrix = ToExpression[matrix];
list = Take[genenames, All, {Position[annotationnames,
       "250_Normal+Tumor"][[1, 2]]}];
matrix = Take[Sort[Join[list, matrix, 2], OrderedQ[{#2, #1}] &], {1, Count[list, {"Y"}]},
    {2, arrays + 1}];
genes = Dimensions[matrix][[1]]

200

(* Fit Asymmetric Generalized Coherent State to the Transcript Length Distribution *)

k1 = 0.26;
k2 = 0.065;
equi = 14;

Clear[a, b, α, β, λ]
λ = 0.76;
f1 = NSolve[Sqrt[(α - k2 / 2) / (α + k2 / 2)] == λ, α];
f2 = FindInstance[{2 * Sqrt[(α /. f1) ^ 2 - β ^ 2] == k2, β > 0}, β];
a = ((α /. f1) + (β /. f2))[[1]]
b = ((α /. f1) - (β /. f2))[[1]] * a / (a - ((α /. f1) - (β /. f2))[[1]])

0.238333

0.00451579

Clear[f, x, p];
f[x_, p_, a_, b_] :=
  If[x < p,
    N[Exp[- (k1 / k2) * a * (x - p) ^ 2]],
    N[Exp[- a * (x - p) ^ 2]]] *
   If[p < 0,
    N[Exp[- (k1 / k2) * b * p ^ 2]],
    N[Exp[- b * p ^ 2]]];
Clear[α, β, λ]

(* Fit Peaks of Transcripts' Profiles' with an Asymmetric Gaussian *)

distribution = Sum[matrix[[n]], {n, 1, genes}];
c = Sort[distribution, OrderedQ[{#2, #1}] &][[1]];
distribution = distribution / c;
correlation = Dot[distribution, Table[f[p, p, a, b], {p, - equi, arrays - equi - 1}]] /
    Sqrt[Dot[distribution, distribution]] /
   Sqrt[Dot[Table[f[p, p, a, b], {p, - equi, arrays - equi - 1}],
     Table[f[p, p, a, b], {p, - equi, arrays - equi - 1}]]];
correlation = Round[100 * correlation] / 100.

0.98
```

```
(* Create Graph Display of the Asymmetric Gaussian Fit *)

graph = Plot[f[p, p, a, b] / f[0, 0, a, b] * distribution[[equi + 1]],
    {p, -equi, arrays - equi - 1},
    PlotStyle → {RGBColor[0, 0, 0], Dashing[{0.03, 0.02}]},
    Axes → False,
    Frame → True,
    PlotRange → {-0.02, 1.02},
    Filling → Bottom, FillingStyle → Opacity[0.1, RGBColor[0, 0, 1]]];

graphG = Plot[f[p - equi - 1, p - equi - 1, a, b] / f[0, 0, a, b] * distribution[[equi + 1]],
    {p, 1, arrays},
    PlotStyle → {RGBColor[0, 0.5, 0], Thickness[0.006]},
    Axes → False,
    Frame → True,
    PlotRange → {-0.02, 1.02},
    Filling → Bottom, FillingStyle → Opacity[0.1, RGBColor[0, 0, 1]]];
distributionG = distribution;

coordinates = Table[{n - equi - 1, distribution[[n]]}, {n, 1, arrays}];
points = Table[Point[coordinates[[n]]], {n, 1, arrays}];
points = Graphics[{RGBColor[0, 0, 0], PointSize[0.022], points}];
lines = Graphics[{RGBColor[0, 0, 0], Line[coordinates]}];

distributionT = Graphics[{Text[ColumnForm[{"Normal ⋂ Tumor",
        StringJoin[ReplaceAll[Characters[arraynames[[1, equi + 1]]], "_" → " "]],
        ToString[correlation]}, Center], {37 - equi - 1, 0.86}]}];
labelx = "(g) Arrays";
framex = Table[{n - equi - 1, Rotate[arraynames[[1, n]], Pi / 2]}, {n, 1, arrays}];
Do[If[Mod[n - 1, 5] ≠ 0, framex[[n, 2]] = Rotate["       ", Pi / 2]],
   {n, 1, Dimensions[framex][[1]]}];
framey = {0.2, 0.4, 0.6, 0.8, 1};
g = Show[{graph, points, lines, distributionT},
    Frame → True,
    FrameLabel → {None, None, labelx, ""},
    FrameTicks → {None, framey, framex, None},
    GridLines → {{{0, RGBColor[0, 0, 0]}}, {{0, RGBColor[0, 0, 0]}}}];
g28 = Show[g,
    PlotRange → {-0.02, 1.02}, AspectRatio → 1.05];
Clear[a, b];

(* Read the Length Data of the Human Transcripts Overexpressed in the GBM Tumor *)

stream = path <> "Data/Human_Transcript_Lengths.txt";
matrix = Import[stream, "Table"];
annotations = Position[matrix[[1]], "124_mm"][[1, 1]] - 1;
{genes, arrays} = Dimensions[matrix] - {1, annotations}
Clear[stream]

{4109, 50}

genenames = Take[matrix, {2, genes + 1}, {1, annotations}];
arraynames = Take[matrix, {1, 1}, {annotations + 1, arrays + annotations}];
annotationnames = Take[matrix, {1, 1}, {1, annotations}];
matrix = Take[matrix, {2, genes + 1}, {annotations + 1, arrays + annotations}];
matrix = ToExpression[matrix];
list = Take[genenames, All, {Position[annotationnames,
       "250_Tumor-Normal"][[1, 2]]}];
matrix = Take[Sort[Join[list, matrix, 2], OrderedQ[{#2, #1}] &], {1, Count[list, {"Y"}]},
    {2, arrays + 1}];
genes = Dimensions[matrix][[1]]

135
```

```
(* Fit Asymmetric Generalized Coherent State to the Transcript Length Distribution *)

k1 = 0.13;
k2 = 0.065;
equi = 17;

Clear[a, b, α, β, λ]
λ = 0.76;
f1 = NSolve[Sqrt[(α - k2 / 2) / (α + k2 / 2)] == λ, α];
f2 = FindInstance[{2 * Sqrt[(α /. f1) ^ 2 - β ^ 2] == k2, β > 0}, β];
a = ((α /. f1) + (β /. f2))[[1]]
b = ((α /. f1) - (β /. f2))[[1]] * a / (a - ((α /. f1) - (β /. f2))[[1]])

0.238333

0.00451579

Clear[f, x, p];
f[x_, p_, a_, b_] :=
  If[x < p,
    N[Exp[- (k1 / k2) * a * (x - p) ^ 2]],
    N[Exp[- a * (x - p) ^ 2]]] *
   If[p < 0,
    N[Exp[- (k1 / k2) * b * p ^ 2]],
    N[Exp[- b * p ^ 2]]];
Clear[α, β, λ]

(* Fit Peaks of Transcripts' Profiles' with an Asymmetric Gaussian *)

distribution = Sum[matrix[[n]], {n, 1, genes}];
distribution = distribution / c;
correlation = Dot[distribution, Table[f[p, p, a, b], {p, - equi, arrays - equi - 1}]] /
    Sqrt[Dot[distribution, distribution]] /
   Sqrt[Dot[Table[f[p, p, a, b], {p, - equi, arrays - equi - 1}],
     Table[f[p, p, a, b], {p, - equi, arrays - equi - 1}]]];
correlation = Round[100 * correlation] / 100.

0.99

(* Create Graph Display of the Asymmetric Gaussian Fit *)

graph = Plot[f[p, p, a, b] / f[0, 0, a, b] * distribution[[equi + 4]],
   {p, - equi, arrays - equi - 1},
   PlotStyle → {RGBColor[0, 0, 0], Dashing[{0.03, 0.02}]},
   Axes → False,
   Frame → True,
   PlotRange → {- 0.02, 1.02},
   Filling → Bottom, FillingStyle → Opacity[0.1, RGBColor[0, 0, 1]]];

graphB = Plot[f[p - equi - 1, p - equi - 1, a, b] / f[0, 0, a, b] * distribution[[equi + 4]],
   {p, 1, arrays},
   PlotStyle → {RGBColor[0, 0, 1], Thickness[0.006]},
   Axes → False,
   Frame → True,
   PlotRange → {- 0.02, 1.02},
   Filling → Bottom, FillingStyle → Opacity[0.1, RGBColor[0, 0, 1]]];
distributionB = distribution;

coordinates = Table[{n - equi - 1, distribution[[n]]}, {n, 1, arrays}];
points = Table[Point[coordinates[[n]]], {n, 1, arrays}];
points = Graphics[{RGBColor[0, 0, 0], PointSize[0.022], points}];
lines = Graphics[{RGBColor[0, 0, 0], Line[coordinates]}];
```

```
distributionT = Graphics[{Text[ColumnForm[{"Tumor \ Normal",
        StringJoin[ReplaceAll[Characters[arraynames[[1, equi + 1]]], "_" → " "]],
        ToString[correlation]}, Center], {37 - equi - 1, 0.86}]}];
labelx = "(f) Arrays";
framex = Table[{n - equi - 1, Rotate[arraynames[[1, n]], Pi / 2]}, {n, 1, arrays}];
Do[If[Mod[n - 1, 5] ≠ 0, framex[[n, 2]] = Rotate["       ", Pi / 2]],
  {n, 1, Dimensions[framex][[1]]}];
framey = {0.2, 0.4, 0.6, 0.8, 1};
g = Show[{graph, points, lines, distributionT},
   Frame → True,
   FrameLabel → {None, None, labelx, None},
   FrameTicks → {None, framey, framex, None},
   GridLines → {{{0, RGBColor[0, 0, 0]}}, {{0, RGBColor[0, 0, 0]}}}];
g27 = Show[g,
   PlotRange → {-0.02, 1.02}, AspectRatio → 1.05];
Clear[a, b];

(* Read the Length Data of the Human Transcripts Overexpressed in the Normal Brain *)

stream = path <> "Data/Human_Transcript_Lengths.txt";
matrix = Import[stream, "Table"];
annotations = Position[matrix[[1]], "124_mm"][[1, 1]] - 1;
{genes, arrays} = Dimensions[matrix] - {1, annotations}
Clear[stream]

{4109, 50}

genenames = Take[matrix, {2, genes + 1}, {1, annotations}];
arraynames = Take[matrix, {1, 1}, {annotations + 1, arrays + annotations}];
annotationnames = Take[matrix, {1, 1}, {1, annotations}];
matrix = Take[matrix, {2, genes + 1}, {annotations + 1, arrays + annotations}];
matrix = ToExpression[matrix];
list = Take[genenames, All, {Position[annotationnames,
       "250_Normal-Tumor"][[1, 2]]}];
matrix = Take[Sort[Join[list, matrix, 2], OrderedQ[{#2, #1}] &], {1, Count[list, {"Y"}]},
    {2, arrays + 1}];
genes = Dimensions[matrix][[1]]

102

(* Fit Asymmetric Generalized Coherent State to the Transcript Length Distribution *)

k1 = 0.065;
k2 = 0.065;
equi = 22;

Clear[a, b, α, β, λ]
λ = 0.76;
f1 = NSolve[Sqrt[(α - k2 / 2) / (α + k2 / 2)] == λ, α];
f2 = FindInstance[{2 * Sqrt[(α /. f1) ^2 - β^2] == k2, β > 0}, β];
a = ((α /. f1) + (β /. f2))[[1]]
b = ((α /. f1) - (β /. f2))[[1]] * a / (a - ((α /. f1) - (β /. f2))[[1]])

0.238333

0.00451579

Clear[f, x, p];
f[x_, p_, a_, b_] :=
  If[x < p,
    N[Exp[- (k1 / k2) * a * (x - p) ^2]],
    N[Exp[-a * (x - p) ^2]]] *
   If[p < 0,
    N[Exp[- (k1 / k2) * b * p^2]],
    N[Exp[-b * p^2]]];
Clear[α, β, λ]
```

```
(* Fit Peaks of Transcripts' Profiles' with an Asymmetric Gaussian *)

distribution = Sum[matrix[[n]], {n, 1, genes}];
distribution = distribution / c;
correlation = Dot[distribution, Table[f[p, p, a, b], {p, -equi, arrays - equi - 1}]] /
     Sqrt[Dot[distribution, distribution]] /
    Sqrt[Dot[Table[f[p, p, a, b], {p, -equi, arrays - equi - 1}],
      Table[f[p, p, a, b], {p, -equi, arrays - equi - 1}]]];
correlation = Round[100 * correlation] / 100.

0.99

(* Create Graph Display of the Asymmetric Gaussian Fit *)

graph = Plot[f[p, p, a, b] / f[0, 0, a, b] * distribution[[equi + 1]],
    {p, -equi, arrays - equi - 1},
    PlotStyle → {RGBColor[0, 0, 0], Dashing[{0.03, 0.02}]},
    Axes → False,
    Frame → True,
    PlotRange → {-0.02, 1.02},
    Filling → Bottom, FillingStyle → Opacity[0.1, RGBColor[0, 0, 1]]];

graphR = Plot[f[p - equi - 1, p - equi - 1, a, b] / f[0, 0, a, b] * distribution[[equi + 1]],
    {p, 1, arrays},
    PlotStyle → {RGBColor[1, 0, 0], Thickness[0.006]},
    Axes → False,
    Frame → True,
    PlotRange → {-0.02, 1.02},
    Filling → Bottom, FillingStyle → Opacity[0.1, RGBColor[0, 0, 1]]];
distributionR = distribution;

coordinates = Table[{n - equi - 1, distribution[[n]]}, {n, 1, arrays}];
points = Table[Point[coordinates[[n]]], {n, 1, arrays}];
points = Graphics[{RGBColor[0, 0, 0], PointSize[0.022], points}];
lines = Graphics[{RGBColor[0, 0, 0], Line[coordinates]}];

distributionT = Graphics[{Text[ColumnForm[{"Normal \ Tumor",
       StringJoin[ReplaceAll[Characters[arraynames[[1, equi + 1]]], "_" → " "]],
       ToString[correlation]}, Center], {37 - equi - 1, 0.86}]}];
labelx = "(e) Arrays";
labely = "Relative mRNA Abundance Level";
framex = Table[{n - equi - 1, Rotate[arraynames[[1, n]], Pi / 2]}, {n, 1, arrays}];
Do[If[Mod[n - 1, 5] ≠ 0, framex[[n, 2]] = Rotate["       ", Pi / 2]],
  {n, 1, Dimensions[framex][[1]]}];
framey = {0.2, 0.4, 0.6, 0.8, 1};
g = Show[{graph, points, lines, distributionT},
    Frame → True,
    FrameLabel → {None, labely, labelx, None},
    FrameTicks → {None, framey, framex, None},
    GridLines → {{{0, RGBColor[0, 0, 0]}}, {{0, RGBColor[0, 0, 0]}}}];
g26 = Show[g,
    PlotRange → {-0.02, 1.02}, AspectRatio → 1.05];
Clear[a, b];
```

```
(* Create Display of the Asymmetric Gaussians that Fit the Subsets' Distributions *)

text = Graphics[{Text[ColumnForm[{
        StyleForm["Normal ⋂ Tumor", FontColor → RGBColor[0, 0.5, 0]],
        StyleForm["Tumor \ Normal", FontColor → RGBColor[0, 0, 1]],
        StyleForm["Normal \ Tumor", FontColor → RGBColor[1, 0, 0]]}, Center], {37, 0.86}]}];
labelx = "(h) Arrays";
framex = Table[{n, Rotate[arraynames[[1, n]], Pi / 2]}, {n, 1, arrays}];
Do[If[Mod[n - 1, 5] ≠ 0, framex[[n, 2]] = Rotate["       ", Pi / 2]],
  {n, 1, Dimensions[framex][[1]]}];
framey = {0.2, 0.4, 0.6, 0.8, 1};
g = Show[{graphG, graphB, graphR, text},
   Frame → True,
   FrameLabel → {None, None, labelx, ColumnForm[{"", ""}, Center]},
   FrameTicks → {None, framey, framex, None},
   GridLines → {{
      {15, {RGBColor[0, 0.5, 0], Thickness[0.004]}},
      {18, {RGBColor[0, 0, 1], Thickness[0.004]}},
      {23, {RGBColor[1, 0, 0], Thickness[0.004]}}},
     {{0, RGBColor[0, 0, 0]}}}];
g29 = Show[g,
   PlotRange → {{1, 50}, {-0.02, 1.02}}, AspectRatio → 1.05];

(* Display SVD and Length Distribution Functions of the Subsets of Human Transcripts *)

g = GraphicsGrid[{{g22, g23, g24, g25}, {g26, g27, g28, g29}}, Spacings → {-25, 20}, ImageSize → 1000];

fig4 = Show[{Graphics[{Rectangle[{0, 0}, {1250, 711}, g]}, ImageSize → 1000],
   Graphics[{RGBColor[0, 0, 0], Text[Style[
       ColumnForm[{"Normal \ Tumor", "Overexpression Subset"}, Center],
       FontSize → 12], {189, 757}]}],
   Graphics[{RGBColor[0, 0, 0], Arrowheads[{-0.024, 0.024}], Arrow[{{57, 723}, {318, 723}}]}],
   Graphics[{RGBColor[0, 0, 0], Line[{{57, 717}, {57, 729}}]}],
   Graphics[{RGBColor[0, 0, 0], Line[{{318, 717}, {318, 729}}]}],
   Graphics[{RGBColor[0, 0, 0], Text[Style[
       ColumnForm[{"Tumor \ Normal", "Overexpression Subset"}, Center],
       FontSize → 12], {480, 757}]}],
   Graphics[{RGBColor[0, 0, 0], Arrowheads[{-0.024, 0.024}], Arrow[{{354, 723}, {615, 723}}]}],
   Graphics[{RGBColor[0, 0, 0], Line[{{354, 717}, {354, 729}}]}],
   Graphics[{RGBColor[0, 0, 0], Line[{{615, 717}, {615, 729}}]}],
   Graphics[{RGBColor[0, 0, 0], Text[Style[
       ColumnForm[{"Normal ⋂ Tumor", "Overexpression Subset"}, Center],
       FontSize → 12], {770, 757}]}],
   Graphics[{RGBColor[0, 0, 0], Arrowheads[{-0.024, 0.024}], Arrow[{{651, 723}, {912, 723}}]}],
   Graphics[{RGBColor[0, 0, 0], Line[{{651, 717}, {651, 729}}]}],
   Graphics[{RGBColor[0, 0, 0], Line[{{912, 717}, {912, 729}}]}],
   Graphics[{RGBColor[0, 0, 0], Text[Style[
       ColumnForm[{"Normal or Tumor", "Overexpression Subsets"}, Center],
       FontSize → 12], {1062, 757}]}],
   Graphics[{RGBColor[0, 0, 0], Arrowheads[{-0.024, 0.024}], Arrow[{{948, 723}, {1209, 723}}]}],
   Graphics[{RGBColor[0, 0, 0], Line[{{948, 717}, {948, 729}}]}],
   Graphics[{RGBColor[0, 0, 0], Line[{{1209, 717}, {1209, 729}}]}],
   Graphics[
    {RGBColor[0, 0, 0], Text[Style[Rotate["Eigenvectors", Pi / 2], FontSize → 12], {-6, 500}]}],
   Graphics[{RGBColor[0, 0, 0], Text[Style[Rotate["Overall Transcript Profiles", Pi / 2],
       FontSize → 12], {-6, 136}]}]
  }]
```

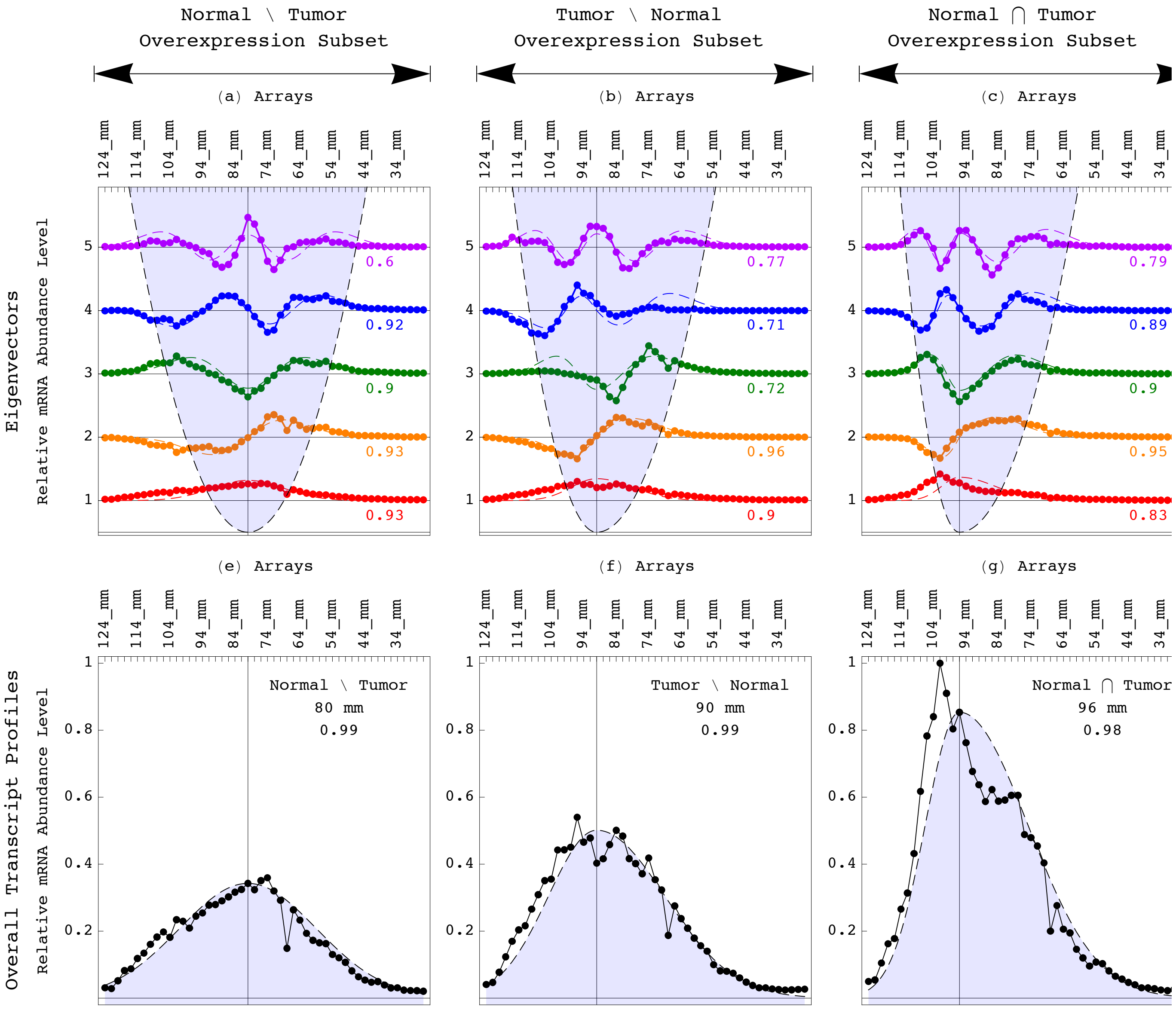


```
Export[path <> "Figures/Figure_4.pdf", fig4, "PDF", ImageSize → 1000, ImageResolution → resolution];
```

```
(* Average Lengths of the Human Subsets Overexpressed in Normal Brain or GBM Tumor *)

(* Read Human Global Transcript Length Data *)

stream = path <> "Data/Human_Transcript_Lengths.txt";
matrix = Import[stream, "Table"];
annotations = Position[matrix[[1]], "124_mm"][[1, 1]] - 1;
{genes, arrays} = Dimensions[matrix] - {1, annotations}
Clear[stream]

{4109, 50}

overexpressed = Take[matrix, {2, genes + 1}, {1, annotations}];
overexpressed = Drop[Drop[Transpose[overexpressed], {4, 12}], {1, 2}];
overexpressed = ReplaceAll[ReplaceAll[overexpressed, "N" → 0], "Y" → 1];
averages = Table[N[Dot[overexpressed[[1]], overexpressed[[a + 5 * b + 1]]] /
      Count[overexpressed[[a + 5 * b + 1]], 1]], {a, 1, 5}, {b, 0, 5}];

labelx = Style[ColumnForm[{"Transcript Lengths"}, Center], FontSize → 12];
labely = "Number of Nucleotides";
framex = Table[{a, 200 + 50 * a}, {a, 1, 6}];
framey = Table[{1500 + a * 200, 1500 + a * 200}, {a, 1, 6}];
framey[[1, 2]] = "  1700";
color = {
   RGBColor[0, 0.5, 0],
   RGBColor[1, 0, 0],
   RGBColor[0.75, 0, 1],
   RGBColor[0, 0, 1],
   RGBColor[1, 0.5, 0]};
points = Table[0, {n, 1, 5}];
lines = Table[0, {n, 1, 5}];
Do[{coordinates = Table[{a, averages[[n, a]]}, {a, 1, 6}],
  points[[n]] = Table[Point[coordinates[[a]]], {a, 1, 6}],
  lines[[n]] = Line[coordinates]}, {n, 1, 5}]
points = Table[Graphics[{color[[Mod[n, 5] + 1]], PointSize[0.025], points[[n]]}], {n, 1, 5}];
lines = Table[Graphics[{Thickness[.0075], color[[Mod[n, 5] + 1]], lines[[n]]}], {n, 1, 5}];
g30 = Show[{points, lines},
   Frame → True,
   FrameLabel → {"(a) Cutoff", labely, labelx, None},
   FrameTicks → {framex, framey, None, None},
   GridLines → {None, {{N[Mean[overexpressed[[1]]]], RGBColor[0, 0, 0]}}},
   PlotRange → {{0.85, 6.15}, {1690, 2710}}, AspectRatio → 1.05];

(* Read Human Global Maximum Gene Length Data *)

stream = path <> "Data/Human_Gene_Lengths.txt";
matrix = Import[stream, "Table"];
{genes, annotations} = Dimensions[matrix] - {1, 0}
Clear[stream]

{11 623, 42}

overexpressed = Take[matrix, {2, genes + 1}, {1, annotations}];
overexpressed = Drop[Drop[Transpose[overexpressed], {3, 12}], {1, 1}];
overexpressed = ReplaceAll[ReplaceAll[overexpressed, "N" → 0], "Y" → 1];
averages = Table[N[Dot[overexpressed[[1]], overexpressed[[a + 5 * b + 1]]] /
      Count[overexpressed[[a + 5 * b + 1]], 1]], {a, 1, 5}, {b, 0, 5}];

labelx = Style[ColumnForm[{"Maximum Gene Lengths"}, Center], FontSize → 12];
framex = Table[{a, 200 + 50 * a}, {a, 1, 6}];
framey = Table[{a * 20 000, a * 20 000}, {a, 1, 6}];
points = Table[0, {n, 1, 5}];
lines = Table[0, {n, 1, 5}];
Do[{coordinates = Table[{a, averages[[n, a]]}, {a, 1, 6}],
  points[[n]] = Table[Point[coordinates[[a]]], {a, 1, 6}],
  lines[[n]] = Line[coordinates]}, {n, 1, 5}]
points = Table[Graphics[{color[[Mod[n, 5] + 1]], PointSize[0.025], points[[n]]}], {n, 1, 5}];
```

```
lines = Table[Graphics[{Thickness[.0075], color[[Mod[n, 5] + 1]], lines[[n]]}], {n, 1, 5}];
g31 = Show[{points, lines},
  Frame → True,
  FrameLabel → {"(b)  Cutoff", "", labelx, None},
  FrameTicks → {framex, framey, None, None},
  GridLines → {None, {{N[Mean[overexpressed[[1]]]], RGBColor[0, 0, 0]}}},
  PlotRange → {{0.85, 6.15}, {19 000, 121 000}}, AspectRatio → 1.05];
```

```
(* Read Human Global Minimum Gene Length Data *)
```

```
stream = path <> "Data/Human_Gene_Lengths.txt";
matrix = Import[stream, "Table"];
{genes, annotations} = Dimensions[matrix] - {1, 0}
Clear[stream]
```

```
{11 623, 42}
```

```
overexpressed = Take[matrix, {2, genes + 1}, {1, annotations}];
overexpressed = Drop[Drop[Transpose[overexpressed], {4, 12}], {1, 2}];
overexpressed = ReplaceAll[ReplaceAll[overexpressed, "N" → 0], "Y" → 1];
averages = Table[N[Dot[overexpressed[[1]], overexpressed[[a + 5 * b + 1]]] /
     Count[overexpressed[[a + 5 * b + 1]], 1]], {a, 1, 5}, {b, 0, 5}];
```

```
labelx = Style[ColumnForm[{"Minimum Gene Lengths"}, Center], FontSize → 12];
framex = Table[{a, 200 + 50 * a}, {a, 1, 6}];
framey = Table[{a * 10 000, a * 10 000}, {a, 1, 6}];
framey[[1, 2]] = " 10000";
points = Table[0, {n, 1, 5}];
lines = Table[0, {n, 1, 5}];
Do[{coordinates = Table[{a, averages[[n, a]]}, {a, 1, 6}],
  points[[n]] = Table[Point[coordinates[[a]]], {a, 1, 6}],
  lines[[n]] = Line[coordinates]}, {n, 1, 5}]
points = Table[Graphics[{color[[Mod[n, 5] + 1]], PointSize[0.025], points[[n]]}], {n, 1, 5}];
lines = Table[Graphics[{Thickness[.0075], color[[Mod[n, 5] + 1]], lines[[n]]}], {n, 1, 5}];
g32 = Show[{points, lines},
   Frame → True,
   FrameLabel → {"(c)  Cutoff", "", labelx, None},
   FrameTicks → {framex, framey, None, None},
   GridLines → {None, {{N[Mean[overexpressed[[1]]]], RGBColor[0, 0, 0]}}},
   PlotRange → {{0.85, 6.15}, {9500, 60 500}}, AspectRatio → 1.05];
```

```
(* Display Average Lengths of the Human Subsets Overexpressed in Normal or Tumor *)
```

```
fig5 = GraphicsGrid[{{g30, g31, g32}}, Spacings → {-18, 0}, ImageSize → 700]
```

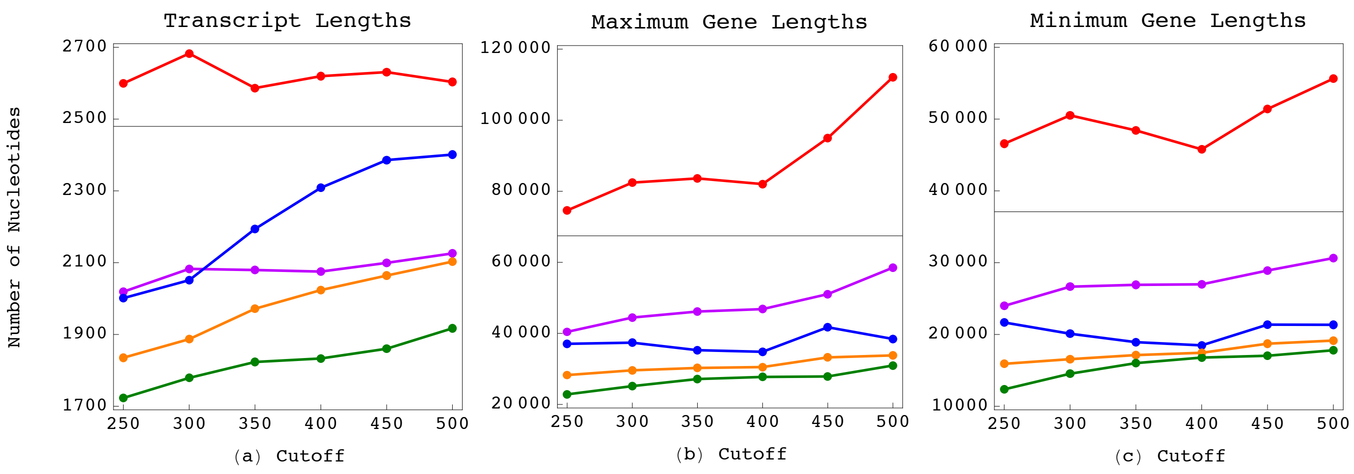


```
Export[path <> "Figures/Figure_5.pdf", fig5, "PDF", ImageSize → 700, ImageResolution → resolution];
```

```
(* Length Distributions and Venn Diagrams of the Subsets of Human Transcripts *)

(* Create Graph Displays of the Length Distributions of the Subsets of Transcripts *)

distributionV = distributionG + distributionR;
distributionO = distributionG + distributionB;
c = Max[Flatten[{distributionV, distributionO}]];

coordinatesG = Table[{n, distributionG[[n]] / c}, {n, 1, arrays}];
pointsG = Table[Point[coordinatesG[[n]]], {n, 1, arrays}];
pointsG = Graphics[{RGBColor[0, 0.5, 0], PointSize[0.022], pointsG}];
linesG = Graphics[{RGBColor[0, 0.5, 0], Line[coordinatesG]}];

coordinatesB = Table[{n, distributionB[[n]] / c}, {n, 1, arrays}];
pointsB = Table[Point[coordinatesB[[n]]], {n, 1, arrays}];
pointsB = Graphics[{RGBColor[0, 0, 1], PointSize[0.022], pointsB}];
linesB = Graphics[{RGBColor[0, 0, 1], Line[coordinatesB]}];

coordinatesR = Table[{n, distributionR[[n]] / c}, {n, 1, arrays}];
pointsR = Table[Point[coordinatesR[[n]]], {n, 1, arrays}];
pointsR = Graphics[{RGBColor[1, 0, 0], PointSize[0.022], pointsR}];
linesR = Graphics[{RGBColor[1, 0, 0], Line[coordinatesR]}];

coordinatesV = Table[{n, distributionV[[n]] / c}, {n, 1, arrays}];
pointsV = Table[Point[coordinatesV[[n]]], {n, 1, arrays}];
pointsV = Graphics[{RGBColor[0.75, 0, 1], PointSize[0.022], pointsV}];
linesV = Graphics[{RGBColor[0.75, 0, 1], Line[coordinatesV]}];

coordinatesO = Table[{n, distributionO[[n]] / c}, {n, 1, arrays}];
pointsO = Table[Point[coordinatesO[[n]]], {n, 1, arrays}];
pointsO = Graphics[{RGBColor[1, 0.5, 0], PointSize[0.022], pointsO}];
linesO = Graphics[{RGBColor[1, 0.5, 0], Line[coordinatesO]}];

text = Graphics[{Text[ColumnForm[{
        StyleForm["Tumor", FontColor → RGBColor[1, 0.5, 0]],
        StyleForm["Normal", FontColor → RGBColor[0.75, 0, 1]], "",
        StyleForm["Normal ⋂ Tumor", FontColor → RGBColor[0, 0.5, 0]],
        StyleForm[StringJoin[ReplaceAll[Characters[arraynames[[1, 15]]], "_" → " "]],
         FontColor → RGBColor[0, 0.5, 0]],
        StyleForm["Tumor \ Normal", FontColor → RGBColor[0, 0, 1]],
        StyleForm[StringJoin[ReplaceAll[Characters[arraynames[[1, 18]]], "_" → " "]],
         FontColor → RGBColor[0, 0, 1]],
        StyleForm["Normal \ Tumor", FontColor → RGBColor[1, 0, 0]],
        StyleForm[StringJoin[ReplaceAll[Characters[arraynames[[1, 23]]], "_" → " "]],
         FontColor → RGBColor[1, 0, 0]], "",
        StyleForm["Human Global", FontColor → RGBColor[0, 0, 0]],
        StyleForm[StringJoin[ReplaceAll[Characters[arraynames[[1, 21]]], "_" → " "]],
         FontColor → RGBColor[0, 0, 0]]}, Center], {40, 0.66}]}];
labelx = "(a) Arrays";
labely = "Relative mRNA Abundance Level";
framex = Table[{n, Rotate[arraynames[[1, n]], Pi / 2]}, {n, 1, arrays}];
Do[If[Mod[n - 1, 5] ≠ 0, framex[[n, 2]] = Rotate["      ", Pi / 2]],
  {n, 1, Dimensions[framex][[1]]}];
framey = {0.2, 0.4, 0.6, 0.8, 1};
g = Show[
   {pointsO, pointsV, pointsG, pointsB, pointsR,
    linesO, linesV, linesG, linesB, linesR,
    text},
   Frame → True,
   FrameLabel → {None, labely, labelx, None},
   FrameTicks → {None, framey, framex, None},
   GridLines → {{
      {15, {RGBColor[0, 0.5, 0], Thickness[0.004]}},
      {18, {RGBColor[0, 0, 1], Thickness[0.004]}},
      {21, {RGBColor[0, 0, 0], Thickness[0.004]}},
      {23, {RGBColor[1, 0, 0], Thickness[0.004]}}},
     {{0, RGBColor[0, 0, 0]}}}];
g37 = Show[{g, Graphics[{Opacity[0.1, RGBColor[0, 0, 1]], Rectangle[{15, -0.02}, {23, 1.02}]}]},
   PlotRange → {{0, 51}, {-0.02, 1.02}}, AspectRatio → 1.05,
   ImageSize → 275];
```

```
text = Graphics[{Text[ColumnForm[{
       StyleForm["Tumor", FontColor → RGBColor[1, 0.5, 0]],
       StyleForm["Normal", FontColor → RGBColor[0.75, 0, 1]], "",
       StyleForm["Normal ⋂ Tumor", FontColor → RGBColor[0, 0.5, 0]],
       StyleForm[StringJoin[ReplaceAll[Characters[arraynames[[1, 15]]], "_" → " "]],
        FontColor → RGBColor[0, 0.5, 0]],
       StyleForm["Normal \ Tumor", FontColor → RGBColor[1, 0, 0]],
       StyleForm[StringJoin[ReplaceAll[Characters[arraynames[[1, 23]]], "_" → " "]],
        FontColor → RGBColor[1, 0, 0]], "", "", "", "", ""}, Center], {40, 0.66}]}];
labelx = "(c) Arrays";
labely = "Relative mRNA Abundance Level";
framex = Table[{n, Rotate[arraynames[[1, n]], Pi / 2]}, {n, 1, arrays}];
Do[If[Mod[n - 1, 5] ≠ 0, framex[[n, 2]] = Rotate["      ", Pi / 2]],
  {n, 1, Dimensions[framex][[1]]}];
framey = {0.2, 0.4, 0.6, 0.8, 1};
g = Show[
   {pointsO, pointsV, pointsG, pointsR,
    linesO, linesV, linesG, linesR,
    text},
   Frame → True,
   FrameLabel → {None, labely, labelx, None},
   FrameTicks → {None, framey, framex, None},
   GridLines → {{
      {15, {RGBColor[0, 0.5, 0], Thickness[0.004]}},
      {23, {RGBColor[1, 0, 0], Thickness[0.004]}}},
     {{0, RGBColor[0, 0, 0]}}}];
g35 = Show[{g, Graphics[{Opacity[0.1, RGBColor[0, 0, 1]], Rectangle[{15, -0.02}, {23, 1.02}]}]},
   PlotRange → {{0, 51}, {-0.02, 1.02}}, AspectRatio → 1.05,
   ImageSize → 275];

text = Graphics[{Text[ColumnForm[{
       StyleForm["Normal ⋂ Tumor", FontColor → RGBColor[0, 0.5, 0]],
       StyleForm[StringJoin[ReplaceAll[Characters[arraynames[[1, 15]]], "_" → " "]],
        FontColor → RGBColor[0, 0.5, 0]],
       StyleForm["Normal \ Tumor", FontColor → RGBColor[1, 0, 0]],
       StyleForm[StringJoin[ReplaceAll[Characters[arraynames[[1, 23]]], "_" → " "]],
        FontColor → RGBColor[1, 0, 0]], "", "", "", "", "", "", "", ""}, Center], {40, 0.66}]}];
labelx = "(e) Arrays";
labely = "Relative mRNA Abundance Level";
framex = Table[{n, Rotate[arraynames[[1, n]], Pi / 2]}, {n, 1, arrays}];
Do[If[Mod[n - 1, 5] ≠ 0, framex[[n, 2]] = Rotate["      ", Pi / 2]],
  {n, 1, Dimensions[framex][[1]]}];
framey = {0.2, 0.4, 0.6, 0.8, 1};
g = Show[
   {pointsG, pointsR,
    linesG, linesR,
    text},
   Frame → True,
   FrameLabel → {None, labely, labelx, None},
   FrameTicks → {None, framey, framex, None},
   GridLines → {{
      {15, {RGBColor[0, 0.5, 0], Thickness[0.004]}},
      {23, {RGBColor[1, 0, 0], Thickness[0.004]}}},
     {{0, RGBColor[0, 0, 0]}}}];
g33 = Show[{g, Graphics[{Opacity[0.1, RGBColor[0, 0, 1]], Rectangle[{15, -0.02}, {23, 1.02}]}]},
   PlotRange → {{0, 51}, {-0.02, 1.02}}, AspectRatio → 1.05,
   ImageSize → 275];
```

```
(* Create Graph Displays of the Venn Diagrams of the Subsets of Human Transcripts *)

labelx = StyleForm[ColumnForm[{"(b) Average Transcript Lengths", "y(M) + x₀", "", ""}, Center],
   FontFamily → "Courier", FontSize → 10, FontColor → RGBColor[0, 0, 0]];
g = Show[{
     RegionPlot[(0.7 ≤ (x + 0.5)^2 + y^2 ≤ 0.8 && (x - 0.5)^2 + y^2 > 0.9) ||
        (0.9 ≤ (x - 0.5)^2 + y^2 ≤ 1 && (x + 0.5)^2 + y^2 < 0.8), {x, -1.5, 1.5}, {y, -1, 1},
      PerformanceGoal → "Quality", PlotPoints → 120, Frame → False, BoundaryStyle → None,
      PlotStyle → RGBColor[1, 0, 0]],
     RegionPlot[(0.7 ≤ (x - 0.5)^2 + y^2 ≤ 0.8 && (x + 0.5)^2 + y^2 > 0.9) ||
        (0.9 ≤ (x + 0.5)^2 + y^2 ≤ 1 && (x - 0.5)^2 + y^2 < 0.8), {x, -1.5, 1.5}, {y, -1, 1},
      PerformanceGoal → "Quality", PlotPoints → 120, Frame → False, BoundaryStyle → None,
      PlotStyle → RGBColor[0, 0, 1]],
     RegionPlot[(0.7 ≤ (x - 0.5)^2 + y^2 ≤ 0.8 && (x + 0.5)^2 + y^2 ≤ 0.8) ||
        (0.7 ≤ (x + 0.5)^2 + y^2 ≤ 0.8 && (x - 0.5)^2 + y^2 ≤ 0.8), {x, -1.5, 1.5}, {y, -1, 1},
      PerformanceGoal → "Quality", PlotPoints → 120, Frame → False, BoundaryStyle → None,
      PlotStyle → RGBColor[0, 0.5, 0]],
     RegionPlot[0.8 ≤ (x + 0.5)^2 + y^2 ≤ 0.9, {x, -1.5, 1.5}, {y, -1, 1},
      PerformanceGoal → "Quality", PlotPoints → 120, Frame → False, BoundaryStyle → None,
      PlotStyle → RGBColor[0.75, 0, 1]],
     RegionPlot[0.8 ≤ (x - 0.5)^2 + y^2 ≤ 0.9, {x, -1.5, 1.5}, {y, -1, 1},
      PerformanceGoal → "Quality", PlotPoints → 120, Frame → False, BoundaryStyle → None,
      PlotStyle → RGBColor[1, 0.5, 0]],
     Graphics[Text[StyleForm[ColumnForm[{"", "2,599 nt", "M = 102"}, Center],
        FontFamily → "Courier", FontSize → 10, FontColor → RGBColor[1, 0, 0]], {-0.9, 0}]],
     Graphics[Text[StyleForm[ColumnForm[{"", "2,001 nt", "M = 135"}, Center],
        FontFamily → "Courier", FontSize → 10, FontColor → RGBColor[0, 0, 1]], {0.9, 0}]],
     Graphics[Text[StyleForm[ColumnForm[{"", "1,723 nt", "M = 200"}, Center],
        FontFamily → "Courier", FontSize → 10, FontColor → RGBColor[0, 0.5, 0]], {0, 0}]],
     Graphics[Text[StyleForm[ColumnForm[{"2,019 nt", "M = 302"}, Center],
        FontFamily → "Courier", FontSize → 10, FontColor → RGBColor[1, 0, 0.75]], {-1.1, -1.1}]],
     Graphics[Text[StyleForm[ColumnForm[{"1,835 nt", "M = 335"}, Center],
        FontFamily → "Courier", FontSize → 10, FontColor → RGBColor[1, 0.5, 0]], {1.1, -1.1}]],
     Graphics[Text[StyleForm[ColumnForm[{"x₀ = 2,480 nt", "N = 4,109"}, Center],
        FontFamily → "Courier", FontSize → 12, FontColor → RGBColor[0, 0, 0]], {1.025, 1.1125}]]},
   PlotRange → {{-1.5, 1.5}, {-1.225, 1.275}}];
g38 = Show[g,
   Frame → True,
   FrameLabel → {None, None, labelx, None},
   FrameTicks → None,
   AspectRatio → 2.5 / 3 * 1.05,
   ImageSize → 330];
```

```
labelx = StyleForm[ColumnForm[{"(d) Average Transcript Length Differences", "y(L) - y(M)", "", ""},
    Center], FontFamily → "Courier", FontSize → 10, FontColor → RGBColor[0, 0, 0]];
g = Show[{
    RegionPlot[(0.7 ≤ (x + 0.5) ^2 + y^2 ≤ 0.8 && (x - 0.5) ^2 + y^2 > 0.9) ||
       (0.9 ≤ (x - 0.5) ^2 + y^2 ≤ 1 && (x + 0.5) ^2 + y^2 < 0.8), {x, -1.5, 1.5}, {y, -1, 1},
     PerformanceGoal → "Quality", PlotPoints → 120, Frame → False, BoundaryStyle → None,
     PlotStyle → RGBColor[1, 0, 0]],
    RegionPlot[(0.7 ≤ (x - 0.5) ^2 + y^2 ≤ 0.8 && (x + 0.5) ^2 + y^2 ≤ 0.8) ||
       (0.7 ≤ (x + 0.5) ^2 + y^2 ≤ 0.8 && (x - 0.5) ^2 + y^2 ≤ 0.8), {x, -1.5, 1.5}, {y, -1, 1},
     PerformanceGoal → "Quality", PlotPoints → 120, Frame → False, BoundaryStyle → None,
     PlotStyle → RGBColor[0, 0.5, 0]],
    RegionPlot[0.8 ≤ (x + 0.5) ^2 + y^2 ≤ 0.9, {x, -1.5, 1.5}, {y, -1, 1},
     PerformanceGoal → "Quality", PlotPoints → 120, Frame → False, BoundaryStyle → None,
     PlotStyle → RGBColor[0.75, 0, 1]],
    RegionPlot[0.8 ≤ (x - 0.5) ^2 + y^2 ≤ 0.9, {x, -1.5, 1.5}, {y, -1, 1},
     PerformanceGoal → "Quality", PlotPoints → 120, Frame → False, BoundaryStyle → None,
     PlotStyle → RGBColor[1, 0.5, 0]],
    Graphics[Text[StyleForm[ColumnForm[{"", "+580 nt", " L = 102"}, Center],
       FontFamily → "Courier", FontSize → 10, FontColor → RGBColor[1, 0, 0]], {-0.9, 0}]],
    Graphics[Text[StyleForm[ColumnForm[{"", "-296 nt", " L = 200"}, Center],
       FontFamily → "Courier", FontSize → 10, FontColor → RGBColor[0, 0.5, 0]], {0, 0}]],
    Graphics[Text[StyleForm[ColumnForm[{"y(M) + x0 = 2,019 nt", " M = 302"}, Center],
       FontFamily → "Courier", FontSize → 12, FontColor → RGBColor[1, 0, 0.75]], {-0.75, -1.2}]],
    Graphics[Text[StyleForm[ColumnForm[{"-184 nt", " L= 335"}, Center],
       FontFamily → "Courier", FontSize → 10, FontColor → RGBColor[1, 0.5, 0]], {1.1, -1.1}]]},
   PlotRange → {{-1.5, 1.5}, {-1.375, 1.125}}];
g36 = Show[g,
   Frame → True,
   FrameLabel → {None, None, labelx, None},
   FrameTicks → None,
   AspectRatio → 2.5 / 3 * 1.05,
   ImageSize → 330];

labelx = StyleForm[ColumnForm[{"(f) Average Transcript Length Differences", "z(L)", "", ""}, Center],
   FontFamily → "Courier", FontSize → 10, FontColor → RGBColor[0, 0, 0]];
g = Show[{
    RegionPlot[(0.7 ≤ (x + 0.5) ^2 + y^2 ≤ 0.8 && (x - 0.5) ^2 + y^2 > 0.9) ||
       (0.85 ≤ (x - 0.5) ^2 + y^2 ≤ 0.95 && (x + 0.5) ^2 + y^2 < 0.8), {x, -1.5, 1.5}, {y, -1, 1},
     PerformanceGoal → "Quality", PlotPoints → 120, Frame → False, BoundaryStyle → None,
     PlotStyle → RGBColor[1, 0, 0]],
    RegionPlot[(0.75 ≤ (x - 0.5) ^2 + y^2 ≤ 0.85 && (x + 0.5) ^2 + y^2 ≤ 0.8) ||
       (0.7 ≤ (x + 0.5) ^2 + y^2 ≤ 0.8 && (x - 0.5) ^2 + y^2 ≤ 0.8), {x, -1.5, 1.5}, {y, -1, 1},
     PerformanceGoal → "Quality", PlotPoints → 120, Frame → False, BoundaryStyle → None,
     PlotStyle → RGBColor[0, 0.5, 0]],
    Graphics[Text[StyleForm[ColumnForm[{"", "+876 nt", " L=102"}, Center],
       FontFamily → "Courier", FontSize → 10, FontColor → RGBColor[1, 0, 0]], {-0.9, 0}]],
    Graphics[Text[StyleForm[ColumnForm[{"", "-876 nt", " L = 200"}, Center],
       FontFamily → "Courier", FontSize → 10, FontColor → RGBColor[0, 0.5, 0]], {0, 0}]],
    Graphics[Text[StyleForm[ColumnForm[{"y(M-L) + x0 = 2,599 nt", "M-L = 102"}, Center],
       FontFamily → "Courier", FontSize → 12, FontColor → RGBColor[1, 0, 0]], {-0.675, -1.2}]],
    Graphics[Text[StyleForm[ColumnForm[{"y(M-L) + x0 = 1,723 nt", "M-L = 200"}, Center],
       FontFamily → "Courier", FontSize → 12, FontColor → RGBColor[0, 0.5, 0]], {0.675, 0.95}]]},
   PlotRange → {{-1.5, 1.5}, {-1.375, 1.125}}];
g34 = Show[g,
   Frame → True,
   FrameLabel → {None, None, labelx, None},
   FrameTicks → None,
   AspectRatio → 2.5 / 3 * 1.05,
   ImageSize → 330];
```

```
(* Display Distributions and Venn Diagrams of the Subsets of Human Transcripts *)
```

```
fig6 = Show[{Graphics[{Rectangle[{0, 0}, {344, 384}, g33]}, ImageSize → 275],
   Graphics[{Rectangle[{354, 0}, {767, 384}, g34]}, ImageSize → 330],
   Graphics[{Rectangle[{0, 404}, {344, 788}, g35]}, ImageSize → 275],
   Graphics[{Rectangle[{354, 404}, {767, 788}, g36]}, ImageSize → 330],
   Graphics[{Rectangle[{0, 808}, {344, 1192}, g37]}, ImageSize → 275],
   Graphics[{Rectangle[{354, 808}, {767, 1192}, g38]}, ImageSize → 330],
   Graphics[{Text[Style["Overall Transcript Profiles", FontSize → 12], {197, 1221}]}],
   Graphics[{RGBColor[0, 0, 0], Arrowheads[{-0.024, 0.024}], Arrow[{{46, 1205}, {343, 1205}}]}],
   Graphics[{RGBColor[0, 0, 0], Line[{{46, 1199}, {46, 1211}}]}],
   Graphics[{RGBColor[0, 0, 0], Line[{{343, 1199}, {343, 1211}}]}],
   Graphics[{Text[Style["Venn Diagrams", FontSize → 12], {532, 1221}]}],
   Graphics[{RGBColor[0, 0, 0], Arrowheads[{-0.024, 0.024}], Arrow[{{354, 1205}, {710, 1205}}]}],
   Graphics[{RGBColor[0, 0, 0], Line[{{354, 1199}, {354, 1211}}]}],
   Graphics[{RGBColor[0, 0, 0], Line[{{710, 1199}, {710, 1211}}]}]
  }, ImageSize → 615]
```

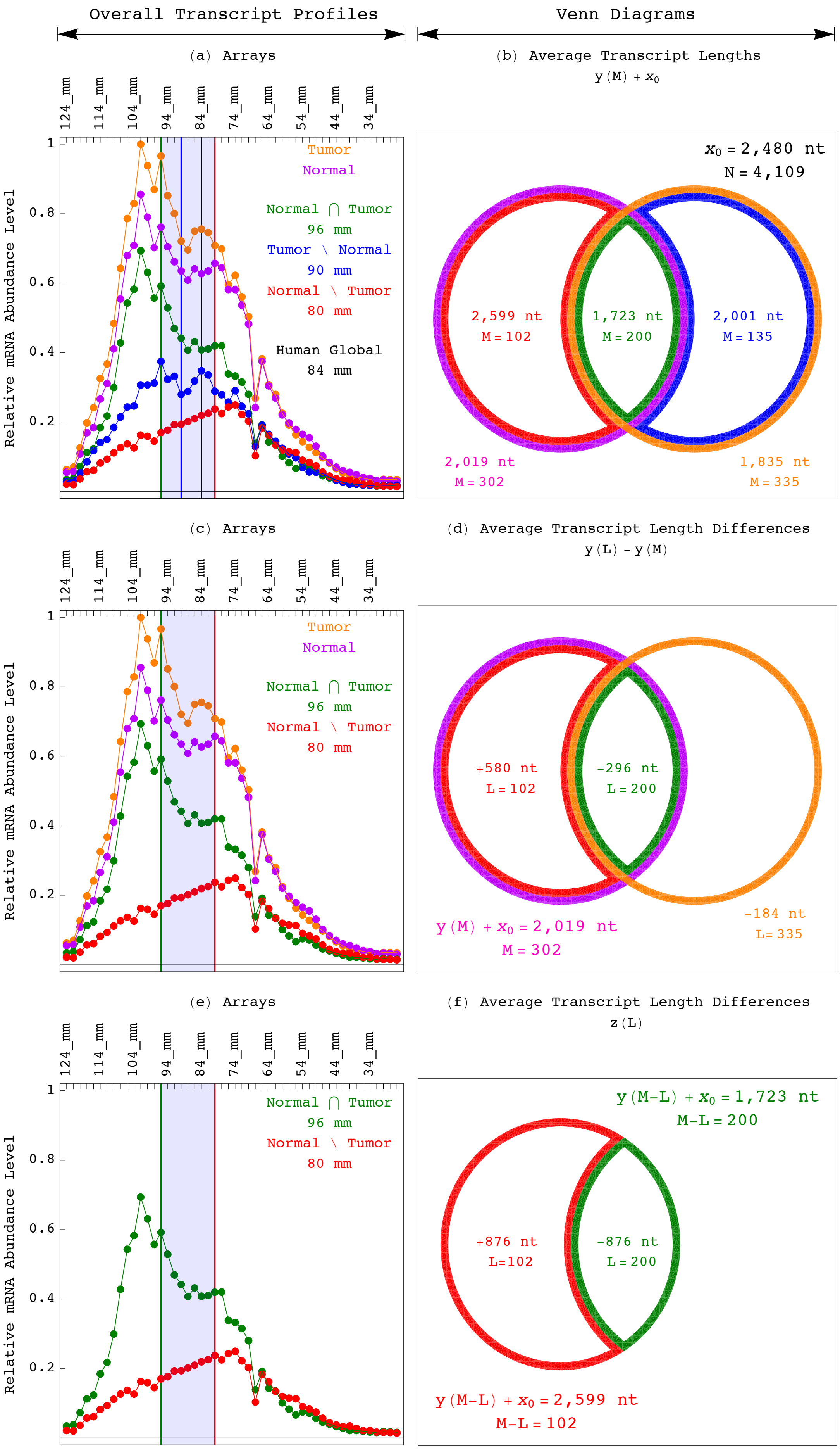

Overall Transcript Profiles
Venn Diagrams
(a) Arrays
(b) Average Transcript Lengths
y(M) + x0
124_mm
114_mm
104_mm
94_mm
84_mm
74_mm
64_mm
54_mm
44_mm
34_mm
Relative mRNA Abundance Level
Tumor
Normal
Normal ⋂ Tumor
96 mm
Tumor \ Normal
90 mm
Normal \ Tumor
80 mm
Human Global
84 mm
x0 = 2,480 nt
N = 4,109
2,599 nt
M = 102
1,723 nt
M = 200
2,001 nt
M = 135
2,019 nt
M = 302
1,835 nt
M = 335
(c) Arrays
(d) Average Transcript Length Differences
y(L) - y(M)
+580 nt
L = 102
-296 nt
L = 200
y(M) + x0 = 2,019 nt
M = 302
-184 nt
L= 335
(e) Arrays
(f) Average Transcript Length Differences
z(L)
y(M-L) + x0 = 1,723 nt
M-L = 200
+876 nt
L=102
-876 nt
L = 200
y(M-L) + x0 = 2,599 nt
M-L = 102

```
Export[path <> "Figures/Figure_6.pdf", fig6, "PDF", ImageSize → 615, ImageResolution → resolution];
```

```
(* SVD of the Transcript Length Data of the Human Mitochondrial Metabolism Subsets *)
```

```
(* Read the Data of the Human Respiratory Electron Transport Chain Subset *)

stream = path <> "Data/Human_Transcript_Lengths.txt";
matrix = Import[stream, "Table"];
annotations = Position[matrix[[1]], "124_mm"][[1, 1]] - 1;
{genes, arrays} = Dimensions[matrix] - {1, annotations}
Clear[stream]
```

```
{4109, 50}
```

```
genenames = Take[matrix, {2, genes + 1}, {1, annotations}];
arraynames = Take[matrix, {1, 1}, {annotations + 1, arrays + annotations}];
annotationnames = Take[matrix, {1, 1}, {1, annotations}];
matrix = Take[matrix, {2, genes + 1}, {annotations + 1, arrays + annotations}];
matrix = ToExpression[matrix];
list = Take[genenames, All, {Position[annotationnames,
       "Respiratory_Electron_Transport_Chain_GO:0022904"][[1, 2]]}];
matrix = Take[Sort[Join[list, matrix, 2], OrderedQ[{#2, #1}] &], {1, Count[list, {"Y"}]},
    {2, arrays + 1}];
Dimensions[matrix]
```

```
{55, 50}
```

```
(* Calculate SVD *)

{eigenarrays, eigenabundances, eigengenes} =
  SingularValueDecomposition[matrix, Min[Dimensions[matrix]]];
eigengenes = Transpose[eigengenes];
eigenabundances = Diagonal[eigenabundances];
list = {1, 2, 3, 4, 5};
Do[{eigengenes[[list[[a]]]] = -eigengenes[[list[[a]]]]}, {a, 1, Dimensions[list][[1]]}]
```

```
(* Fit Eigenvectors with a Series of Asymmetric Hermite Functions *)

k1 = 0.195;
k2 = 0.0325;
equi = 12;

Clear[g, h];
h[x_, n_, gamma_] := Exp[-gamma * x ^ 2 / 2] * HermiteH[n, Sqrt[gamma] * x] *
    Sqrt[Sqrt[gamma / Pi] / Factorial[n] / (2 ^ n)];
g[x_, n_] := If[x - equi < 0, h[x - equi, n - 1, k1] * (1 / k1) ^ 0.25,
    h[x - equi, n - 1, k2] * (1 / k2) ^ 0.25];
normalization = Table[
    Sqrt[Sum[g[x, n] ^ 2., {x, 0, arrays - 1}]],
    {n, 1, 5}];
correlation = Table[
    Sum[g[x, n] * eigengenes[[n, x + 1]] / normalization[[n]], {x, 0, arrays - 1}],
    {n, 1, 5}];
meancorrelation = Round[100. * Sqrt[Sum[0.2 * correlation[[n]] ^ 2, {n, 1, 5}]]] / 100.
correlation = Round[100. * correlation] / 100.;
```

```
0.79
```

```
(* Fit Differential Equation with an Asymmetric Parabola *)

Clear[f];
f[x_] := If[(x - equi) < 0, 0.5 * k1 * (x - equi) ^ 2, 0.5 * k2 * (x - equi) ^ 2];
```

```
(* Create Selected Eigenvectors Graph Display with Fitting Graphs *)

labelx = "(a) Arrays";
labely = "Relative mRNA Abundance Level";
framex = Table[{a - 1, Rotate[arraynames[[1, a]], Pi / 2]}, {a, 1, arrays}];
Do[If[Mod[a - 1, 5] ≠ 0, framex[[a, 2]] = Rotate["      ", Pi / 2]], {a, 1, Dimensions[framex][[1]]}]
framey = Table[{n - 0.5, n}, {n, 1, 5}];
color = {
   RGBColor[0.75, 0, 1],
   RGBColor[1, 0, 0],
   RGBColor[1, 0.5, 0],
   RGBColor[0, 0.5, 0],
   RGBColor[0, 0, 1]};
points = Table[0, {n, 1, 5}];
lines = Table[0, {n, 1, 5}];
Do[{coordinates = Table[{a - 1, eigengenes[[n, a]] + n - 0.5}, {a, 1, arrays}],
  points[[n]] = Table[Point[coordinates[[a]]], {a, 1, arrays}],
  lines[[n]] = Line[coordinates]}, {n, 1, 5}]
points = Table[Graphics[{color[[Mod[n, 5] + 1]], PointSize[0.022], points[[n]]}], {n, 1, 5}];
lines = Table[Graphics[{Thickness[.005], color[[Mod[n, 5] + 1]], lines[[n]]}], {n, 1, 5}];
graphs = Table[Plot[g[x, n] / normalization[[n]] + n - 0.5, {x, 0, arrays - 1},
    PlotStyle → {color[[Mod[n, 5] + 1]], Dashing[{0.03, 0.02}]}, PlotRange → All], {n, 1, 5}];
texts = Table[Graphics[{color[[Mod[n, 5] + 1]], Text[correlation[[n]], {40, n - 0.75}, {-1, 0}]}],
   {n, 1, 5}];
inflection = Plot[f[x], {x, equi - Sqrt[5.45 * 2 / k1], equi + Sqrt[5.45 * 2 / k2]},
   PlotStyle → {RGBColor[0, 0, 0], Dashing[{0.03, 0.02}]},
   Filling → Top, FillingStyle → Opacity[0.1, RGBColor[0, 0, 1]]];

g39 = Show[{points, lines, graphs, texts, inflection},
   Frame → True,
   FrameLabel → {None, labely, labelx, None},
   FrameTicks → {None, framey, framex, None},
   GridLines → {{{equi, RGBColor[0, 0, 0]}},
     Join[{{0, RGBColor[0, 0, 0]}}, Table[{a - 0.5, RGBColor[0, 0, 0]}, {a, 1, 5}]]},
   PlotRange → {-0.05, 5.45}, AspectRatio → 1.05];

(* Read the Length Data of the Human Mitochondrial Respiratory Chain Complex I Subset *)

stream = path <> "Data/Human_Transcript_Lengths.txt";
matrix = Import[stream, "Table"];
annotations = Position[matrix[[1]], "124_mm"][[1, 1]] - 1;
{genes, arrays} = Dimensions[matrix] - {1, annotations}
Clear[stream]

{4109, 50}

genenames = Take[matrix, {2, genes + 1}, {1, annotations}];
arraynames = Take[matrix, {1, 1}, {annotations + 1, arrays + annotations}];
annotationnames = Take[matrix, {1, 1}, {1, annotations}];
matrix = Take[matrix, {2, genes + 1}, {annotations + 1, arrays + annotations}];
matrix = ToExpression[matrix];
list = Take[genenames, All, {Position[annotationnames,
      "Mitochondrial_Respiratory_Chain_Complex_I_GO:0005747"][[1, 2]]}];
matrix = Take[Sort[Join[list, matrix, 2], OrderedQ[{#2, #1}] &], {1, Count[list, {"Y"}]},
   {2, arrays + 1}];
Dimensions[matrix]

{25, 50}

(* Calculate SVD *)

{eigenarrays, eigenabundances, eigengenes} =
  SingularValueDecomposition[matrix, Min[Dimensions[matrix]]];
eigengenes = Transpose[eigengenes];
eigenabundances = Diagonal[eigenabundances];
list = {1, 3, 5};
Do[{eigengenes[[list[[a]]]] = -eigengenes[[list[[a]]]]}, {a, 1, Dimensions[list][[1]]}]
```

```
(* Fit Eigenvectors with a Series of Asymmetric Hermite Functions *)

k1 = 0.195;
k2 = 0.0325;
equi = 11;

Clear[g, h];
h[x_, n_, gamma_] := Exp[-gamma * x^2 / 2] * HermiteH[n, Sqrt[gamma] * x] *
   Sqrt[Sqrt[gamma / Pi] / Factorial[n] / (2^n)];
g[x_, n_] := If[x - equi < 0, h[x - equi, n - 1, k1] * (1 / k1)^0.25,
   h[x - equi, n - 1, k2] * (1 / k2)^0.25];
normalization = Table[
   Sqrt[Sum[g[x, n]^2., {x, 0, arrays - 1}]],
   {n, 1, 5}];
correlation = Table[
   Sum[g[x, n] * eigengenes[[n, x + 1]] / normalization[[n]], {x, 0, arrays - 1}],
   {n, 1, 5}];
meancorrelation = Round[100. * Sqrt[Sum[0.2 * correlation[[n]]^2, {n, 1, 5}]]] / 100.
correlation = Round[100. * correlation] / 100.;

0.85

(* Fit Differential Equation with an Asymmetric Parabola *)

Clear[f];
f[x_] := If[(x - equi) < 0, 0.5 * k1 * (x - equi)^2, 0.5 * k2 * (x - equi)^2];

(* Create Selected Eigenvectors Graph Display with Fitting Graphs *)

labelx = "(b) Arrays";
framex = Table[{a - 1, Rotate[arraynames[[1, a]], Pi / 2]}, {a, 1, arrays}];
Do[If[Mod[a - 1, 5] ≠ 0, framex[[a, 2]] = Rotate["      ", Pi / 2]], {a, 1, Dimensions[framex][[1]]}]
framey = Table[{n - 0.5, n}, {n, 1, 5}];
points = Table[0, {n, 1, 5}];
lines = Table[0, {n, 1, 5}];

Do[{coordinates = Table[{a - 1, eigengenes[[n, a]] + n - 0.5}, {a, 1, arrays}],
  points[[n]] = Table[Point[coordinates[[a]]], {a, 1, arrays}],
  lines[[n]] = Line[coordinates]}, {n, 1, 5}]
points = Table[Graphics[{color[[Mod[n, 5] + 1]], PointSize[0.022], points[[n]]}], {n, 1, 5}];
lines = Table[Graphics[{Thickness[.005], color[[Mod[n, 5] + 1]], lines[[n]]}], {n, 1, 5}];
graphs = Table[Plot[g[x, n] / normalization[[n]] + n - 0.5, {x, 0, arrays - 1},
    PlotStyle → {color[[Mod[n, 5] + 1]], Dashing[{0.03, 0.02}]}, PlotRange → All], {n, 1, 5}];
texts = Table[Graphics[{color[[Mod[n, 5] + 1]], Text[correlation[[n]], {40, n - 0.75}, {-1, 0}]}],
   {n, 1, 5}];
inflection = Plot[f[x], {x, equi - Sqrt[5.45 * 2 / k1], equi + Sqrt[5.45 * 2 / k2]},
   PlotStyle → {RGBColor[0, 0, 0], Dashing[{0.03, 0.02}]},
   Filling → Top, FillingStyle → Opacity[0.1, RGBColor[0, 0, 1]]];

g40 = Show[{points, lines, graphs, texts, inflection},
   Frame → True,
   FrameLabel → {None, None, labelx, None},
   FrameTicks → {None, framey, framex, None},
   GridLines → {{{equi, RGBColor[0, 0, 0]}},
     Join[{{0, RGBColor[0, 0, 0]}}, Table[{a - 0.5, RGBColor[0, 0, 0]}, {a, 1, 5}]]},
   PlotRange → {-0.05, 5.45}, AspectRatio → 1.05];
```

```
(* Read the Transcript Length Data of the Human Cytochrome-c Oxidase Activity Subset *)

stream = path <> "Data/Human_Transcript_Lengths.txt";
matrix = Import[stream, "Table"];
annotations = Position[matrix[[1]], "124_mm"][[1, 1]] - 1;
{genes, arrays} = Dimensions[matrix] - {1, annotations}
Clear[stream]

{4109, 50}

genenames = Take[matrix, {2, genes + 1}, {1, annotations}];
arraynames = Take[matrix, {1, 1}, {annotations + 1, arrays + annotations}];
annotationnames = Take[matrix, {1, 1}, {1, annotations}];
matrix = Take[matrix, {2, genes + 1}, {annotations + 1, arrays + annotations}];
matrix = ToExpression[matrix];
list = Take[genenames, All, {Position[annotationnames,
       "Cytochrome-c_Oxidase_Activity_GO:0004129"][[1, 2]]}];
matrix = Take[Sort[Join[list, matrix, 2], OrderedQ[{#2, #1}] &], {1, Count[list, {"Y"}]},
    {2, arrays + 1}];
Dimensions[matrix]

{14, 50}

(* Calculate SVD *)

{eigenarrays, eigenabundances, eigengenes} =
  SingularValueDecomposition[matrix, Min[Dimensions[matrix]]];
eigengenes = Transpose[eigengenes];
eigenabundances = Diagonal[eigenabundances];
list = {1, 3, 4, 5};
Do[{eigengenes[[list[[a]]]] = -eigengenes[[list[[a]]]]}, {a, 1, Dimensions[list][[1]]}]

(* Fit Eigenvectors with a Series of Asymmetric Hermite Functions *)

k1 = 0.195;
k2 = 0.0975;
equi = 11;

Clear[g, h];
h[x_, n_, gamma_] := Exp[-gamma * x^2 / 2] * HermiteH[n, Sqrt[gamma] * x] *
    Sqrt[Sqrt[gamma / Pi] / Factorial[n] / (2^n)];
g[x_, n_] := If[x - equi < 0, h[x - equi, n - 1, k1] * (1 / k1) ^0.25,
    h[x - equi, n - 1, k2] * (1 / k2) ^0.25];
normalization = Table[
    Sqrt[Sum[g[x, n]^2., {x, 0, arrays - 1}]],
    {n, 1, 5}];
correlation = Table[
    Sum[g[x, n] * eigengenes[[n, x + 1]] / normalization[[n]], {x, 0, arrays - 1}],
    {n, 1, 5}];
meancorrelation = Round[100. * Sqrt[Sum[0.2 * correlation[[n]]^2, {n, 1, 5}]]] / 100.
correlation = Round[100. * correlation] / 100.;

0.78

(* Fit Differential Equation with an Asymmetric Parabola *)

Clear[f];
f[x_] := If[(x - equi) < 0, 0.5 * k1 * (x - equi)^2, 0.5 * k2 * (x - equi)^2];
```

```
(* Create Selected Eigenvectors Graph Display with Fitting Graphs *)

labelx = "(c) Arrays";
framex = Table[{a - 1, Rotate[arraynames[[1, a]], Pi / 2]}, {a, 1, arrays}];
Do[If[Mod[a - 1, 5] ≠ 0, framex[[a, 2]] = Rotate["       ", Pi / 2]], {a, 1, Dimensions[framex][[1]]}]
framey = Table[{n - 0.5, n}, {n, 1, 5}];
points = Table[0, {n, 1, 5}];
lines = Table[0, {n, 1, 5}];
Do[{coordinates = Table[{a - 1, eigengenes[[n, a]] + n - 0.5}, {a, 1, arrays}],
  points[[n]] = Table[Point[coordinates[[a]]], {a, 1, arrays}],
  lines[[n]] = Line[coordinates]}, {n, 1, 5}]
points = Table[Graphics[{color[[Mod[n, 5] + 1]], PointSize[0.022], points[[n]]}], {n, 1, 5}];
lines = Table[Graphics[{Thickness[.005], color[[Mod[n, 5] + 1]], lines[[n]]}], {n, 1, 5}];
graphs = Table[Plot[g[x, n] / normalization[[n]] + n - 0.5, {x, 0, arrays - 1},
    PlotStyle → {color[[Mod[n, 5] + 1]], Dashing[{0.03, 0.02}]}, PlotRange → All], {n, 1, 5}];
texts = Table[Graphics[{color[[Mod[n, 5] + 1]], Text[correlation[[n]], {40, n - 0.75}, {-1, 0}]}],
   {n, 1, 5}];
inflection = Plot[f[x], {x, equi - Sqrt[5.45 * 2 / k1], equi + Sqrt[5.45 * 2 / k2]},
   PlotStyle → {RGBColor[0, 0, 0], Dashing[{0.03, 0.02}]},
   Filling → Top, FillingStyle → Opacity[0.1, RGBColor[0, 0, 1]]];

g41 = Show[{points, lines, graphs, texts, inflection},
   Frame → True,
   FrameLabel → {None, None, labelx, ""},
   FrameTicks → {None, framey, framex, None},
   GridLines → {{{equi, RGBColor[0, 0, 0]}},
     Join[{{0, RGBColor[0, 0, 0]}}, Table[{a - 0.5, RGBColor[0, 0, 0]}, {a, 1, 5}]]},
   PlotRange → {-0.05, 5.45}, AspectRatio → 1.05];

(* SVD of the Transcript Length Data of the Yeast Mitochondrial Metabolism Subsets *)

(* Read the Data of the Yeast Respiratory Electron Transport Chain Subset *)

stream = path <> "Data/Yeast_Transcript_Lengths.txt";
matrix = Import[stream, "Table"];
annotations = Position[matrix[[1]], "100_mm"][[1, 1]] - 1;
{genes, arrays} = Dimensions[matrix] - {1, annotations}
Clear[stream]

{3620, 30}

genenames = Take[matrix, {2, genes + 1}, {1, annotations}];
arraynames = Take[matrix, {1, 1}, {annotations + 1, arrays + annotations}];
annotationnames = Take[matrix, {1, 1}, {1, annotations}];
matrix = Take[matrix, {2, genes + 1}, {annotations + 1, arrays + annotations}];
matrix = ToExpression[matrix];
list = Take[genenames, All, {Position[annotationnames,
     "Respiratory_Electron_Transport_Chain_GO:0022904"][[1, 2]]}];
matrix = Take[Sort[Join[list, matrix, 2], OrderedQ[{#2, #1}] &], {1, Count[list, {"Y"}]},
  {2, arrays + 1}];
Dimensions[matrix]

{22, 30}

(* Calculate SVD *)

{eigenarrays, eigenabundances, eigengenes} =
  SingularValueDecomposition[matrix, Min[Dimensions[matrix]]];
eigengenes = Transpose[eigengenes];
eigenabundances = Diagonal[eigenabundances];
list = {1, 2, 3, 5};
Do[{eigengenes[[list[[a]]]] = -eigengenes[[list[[a]]]]}, {a, 1, Dimensions[list][[1]]}]
```

```
(* Fit Eigenvectors with a Series of Asymmetric Hermite Functions *)

k1 = 0.4;
k2 = 0.2;
equi = 9;

Clear[g, h];
h[x_, n_, gamma_] := Exp[-gamma * x^2 / 2] * HermiteH[n, Sqrt[gamma] * x] *
   Sqrt[Sqrt[gamma / Pi] / Factorial[n] / (2^n)];
g[x_, n_] := If[x - equi < 0, h[x - equi, n - 1, k1] * (1 / k1) ^0.25,
   h[x - equi, n - 1, k2] * (1 / k2) ^0.25];
normalization = Table[
   Sqrt[Sum[g[x, n] ^2., {x, 0, arrays - 1}]],
   {n, 1, 5}];
correlation = Table[
   Sum[g[x, n] * eigengenes[[n, x + 1]] / normalization[[n]], {x, 0, arrays - 1}],
   {n, 1, 5}];
meancorrelation = Round[100. * Sqrt[Sum[0.2 * correlation[[n]] ^2, {n, 1, 5}]]] / 100.
correlation = Round[100. * correlation] / 100.;

0.7

(* Fit Differential Equation with an Asymmetric Parabola *)

Clear[f];
f[x_] := If[(x - equi) < 0, 0.5 * k1 * (x - equi) ^2, 0.5 * k2 * (x - equi) ^2];

(* Create Selected Eigenvectors Graph Display with Fitting Graphs *)

labelx = "(d) Arrays";
labely = "Relative mRNA Abundance Level";
framex = Table[{a - 1, Rotate[arraynames[[1, a]], Pi / 2]}, {a, 1, arrays}];
Do[If[Mod[a + 3, 3] ≠ 0, framex[[a, 2]] = Rotate["       ", Pi / 2]], {a, 1, Dimensions[framex][[1]]}]
framey = Table[{n - 0.5, n}, {n, 1, 5}];
points = Table[0, {n, 1, 5}];
lines = Table[0, {n, 1, 5}];
Do[{coordinates = Table[{a - 1, eigengenes[[n, a]] + n - 0.5}, {a, 1, arrays}],
  points[[n]] = Table[Point[coordinates[[a]]], {a, 1, arrays}],
  lines[[n]] = Line[coordinates]}, {n, 1, 5}]
points = Table[Graphics[{color[[Mod[n, 5] + 1]], PointSize[0.022], points[[n]]}], {n, 1, 5}];
lines = Table[Graphics[{Thickness[.005], color[[Mod[n, 5] + 1]], lines[[n]]}], {n, 1, 5}];
graphs = Table[Plot[g[x, n] / normalization[[n]] + n - 0.5, {x, 0, arrays - 1},
    PlotStyle → {color[[Mod[n, 5] + 1]], Dashing[{0.03, 0.02}]}, PlotRange → All], {n, 1, 5}];
texts = Table[Graphics[{color[[Mod[n, 5] + 1]], Text[correlation[[n]], {24, n - 0.75}, {-1, 0}]}],
   {n, 1, 5}];
inflection = Plot[f[x], {x, equi - Sqrt[5.45 * 2 / k1], equi + Sqrt[5.45 * 2 / k2]},
   PlotStyle → {RGBColor[0, 0, 0], Dashing[{0.03, 0.02}]},
   Filling → Top, FillingStyle → Opacity[0.1, RGBColor[0, 0, 1]]];

g42 = Show[{points, lines, graphs, texts, inflection},
   Frame → True,
   FrameLabel → {None, labely, labelx, None},
   FrameTicks → {None, framey, framex, None},
   GridLines → {{{equi, RGBColor[0, 0, 0]}},
     Join[{{0, RGBColor[0, 0, 0]}}, Table[{a - 0.5, RGBColor[0, 0, 0]}, {a, 1, 5}]]},
   PlotRange → {-0.05, 5.45}, AspectRatio → 1.05];
```

```
(* Read the Transcript Length Data the Yeast Cytochrome-c Oxidase Activity Subset *)

stream = path <> "Data/Yeast_Transcript_Lengths.txt";
matrix = Import[stream, "Table"];
annotations = Position[matrix[[1]], "100_mm"][[1, 1]] - 1;
{genes, arrays} = Dimensions[matrix] - {1, annotations}
Clear[stream]

{3620, 30}

genenames = Take[matrix, {2, genes + 1}, {1, annotations}];
arraynames = Take[matrix, {1, 1}, {annotations + 1, arrays + annotations}];
annotationnames = Take[matrix, {1, 1}, {1, annotations}];
matrix = Take[matrix, {2, genes + 1}, {annotations + 1, arrays + annotations}];
matrix = ToExpression[matrix];
list = Take[genenames, All, {Position[annotationnames,
       "Cytochrome-c_Oxidase_Activity_GO:0004129"][[1, 2]]}];
matrix = Take[Sort[Join[list, matrix, 2], OrderedQ[{#2, #1}] &], {1, Count[list, {"Y"}]},
    {2, arrays + 1}];
Dimensions[matrix]

{8, 30}

(* Calculate SVD *)

{eigenarrays, eigenabundances, eigengenes} =
  SingularValueDecomposition[matrix, Min[Dimensions[matrix]]];
eigengenes = Transpose[eigengenes];
eigenabundances = Diagonal[eigenabundances];
list = {1, 2, 3, 5};
Do[{eigengenes[[list[[a]]]] = -eigengenes[[list[[a]]]]}, {a, 1, Dimensions[list][[1]]}]
{eigengenes[[4]], eigengenes[[5]]} = {eigengenes[[5]], eigengenes[[4]]};

(* Fit Eigenvectors with a Series of Asymmetric Hermite Functions *)

k1 = 0.4;
k2 = 0.2;
equi = 8;

Clear[g, h];
h[x_, n_, gamma_] := Exp[-gamma * x^2 / 2] * HermiteH[n, Sqrt[gamma] * x] *
    Sqrt[Sqrt[gamma / Pi] / Factorial[n] / (2^n)];
g[x_, n_] := If[x - equi < 0, h[x - equi, n - 1, k1] * (1 / k1) ^0.25,
    h[x - equi, n - 1, k2] * (1 / k2) ^0.25];
normalization = Table[
    Sqrt[Sum[g[x, n]^2., {x, 0, arrays - 1}]],
    {n, 1, 5}];
correlation = Table[
    Sum[g[x, n] * eigengenes[[n, x + 1]] / normalization[[n]], {x, 0, arrays - 1}],
    {n, 1, 5}];
meancorrelation = Round[100. * Sqrt[Sum[0.2 * correlation[[n]]^2, {n, 1, 5}]]] / 100.
correlation = Round[100. * correlation] / 100.;

0.76

(* Fit Differential Equation with an Asymmetric Parabola *)

Clear[f];
f[x_] := If[(x - equi) < 0, 0.5 * k1 * (x - equi)^2, 0.5 * k2 * (x - equi)^2];
```

```
(* Create Selected Eigenvectors Graph Display with Fitting Graphs *)

labelx = "(e) Arrays";
framex = Table[{a - 1, Rotate[arraynames[[1, a]], Pi / 2]}, {a, 1, arrays}];
Do[If[Mod[a + 3, 3] ≠ 0, framex[[a, 2]] = Rotate["       ", Pi / 2]],
 {a, 1, Dimensions[framex][[1]]}]
framey = Table[{n - 0.5, n}, {n, 1, 5}];
points = Table[0, {n, 1, 5}];
lines = Table[0, {n, 1, 5}];

Do[{coordinates = Table[{a - 1, eigengenes[[n, a]] + n - 0.5}, {a, 1, arrays}],
  points[[n]] = Table[Point[coordinates[[a]]], {a, 1, arrays}],
  lines[[n]] = Line[coordinates]}, {n, 1, 5}]
points = Table[Graphics[{color[[Mod[n, 5] + 1]], PointSize[0.022], points[[n]]}], {n, 1, 5}];
lines = Table[Graphics[{Thickness[.005], color[[Mod[n, 5] + 1]], lines[[n]]}], {n, 1, 5}];
graphs = Table[Plot[g[x, n] / normalization[[n]] + n - 0.5, {x, 0, arrays - 1},
    PlotStyle → {color[[Mod[n, 5] + 1]], Dashing[{0.03, 0.02}]}, PlotRange → All], {n, 1, 5}];
texts = Table[Graphics[{color[[Mod[n, 5] + 1]], Text[correlation[[n]], {24, n - 0.75}, {-1, 0}]}],
   {n, 1, 5}];
inflection = Plot[f[x], {x, equi - Sqrt[5.45 * 2 / k1], equi + Sqrt[5.45 * 2 / k2]},
   PlotStyle → {RGBColor[0, 0, 0], Dashing[{0.03, 0.02}]},
   Filling → Top, FillingStyle → Opacity[0.1, RGBColor[0, 0, 1]]];

g43 = Show[{points, lines, graphs, texts, inflection},
   Frame → True,
   FrameLabel → {None, None, labelx, ""},
   FrameTicks → {None, framey, framex, None},
   GridLines → {{{equi, RGBColor[0, 0, 0]}},
     Join[{{0, RGBColor[0, 0, 0]}}, Table[{a - 0.5, RGBColor[0, 0, 0]}, {a, 1, 5}]]},
   PlotRange → {-0.05, 5.45}, AspectRatio → 1.05];

(* Display the SVD of the Human and Yeast Mitochondrial Metabolism Subsets *)

g = GraphicsGrid[{{g39, g40, g41}, {g42, , g43}}, Spacings → {-45, 20}, ImageSize → 700];

figS1 = Show[{Graphics[{Rectangle[{0, 0}, {875, 681}, g]}, ImageSize → 700],
   Graphics[{RGBColor[0, 0, 0], Text[Style[
        ColumnForm[{"Respiratory Electron", "Transport Chain (ETC)", "Subset"}, Center],
        FontSize → 12], {174, 730}]}],
   Graphics[{RGBColor[0, 0, 0], Arrowheads[{-0.024, 0.024}], Arrow[{{51, 693}, {297, 693}}]}],
   Graphics[{RGBColor[0, 0, 0], Line[{{51, 687}, {51, 699}}]}],
   Graphics[{RGBColor[0, 0, 0], Line[{{297, 687}, {297, 699}}]}],
   Graphics[{RGBColor[0, 0, 0], Text[Style[
        ColumnForm[{"Mitochndrial Respiratory", "Chain Complex (MRCC) I", "Subset"}, Center],
        FontSize → 12], {442, 730}]}],
   Graphics[{RGBColor[0, 0, 0], Arrowheads[{-0.024, 0.024}], Arrow[{{319, 693}, {565, 693}}]}],
   Graphics[{RGBColor[0, 0, 0], Line[{{319, 687}, {319, 699}}]}],
   Graphics[{RGBColor[0, 0, 0], Line[{{565, 687}, {565, 699}}]}],
   Graphics[{RGBColor[0, 0, 0], Text[Style[
        ColumnForm[{"Cytochrome-c Oxidase", "(COX) Activity", "Subset"}, Center],
        FontSize → 12], {710, 730}]}],
   Graphics[{RGBColor[0, 0, 0], Arrowheads[{-0.024, 0.024}], Arrow[{{587, 693}, {833, 693}}]}],
   Graphics[{RGBColor[0, 0, 0], Line[{{587, 687}, {587, 699}}]}],
   Graphics[{RGBColor[0, 0, 0], Line[{{834, 687}, {834, 699}}]}],
   Graphics[{RGBColor[0, 0, 0], Text[Style[Rotate["Human", Pi / 2], FontSize → 12], {0, 479}]}],
   Graphics[{RGBColor[0, 0, 0], Text[Style[Rotate["Yeast", Pi / 2], FontSize → 12], {0, 130}]}]
  }]
```

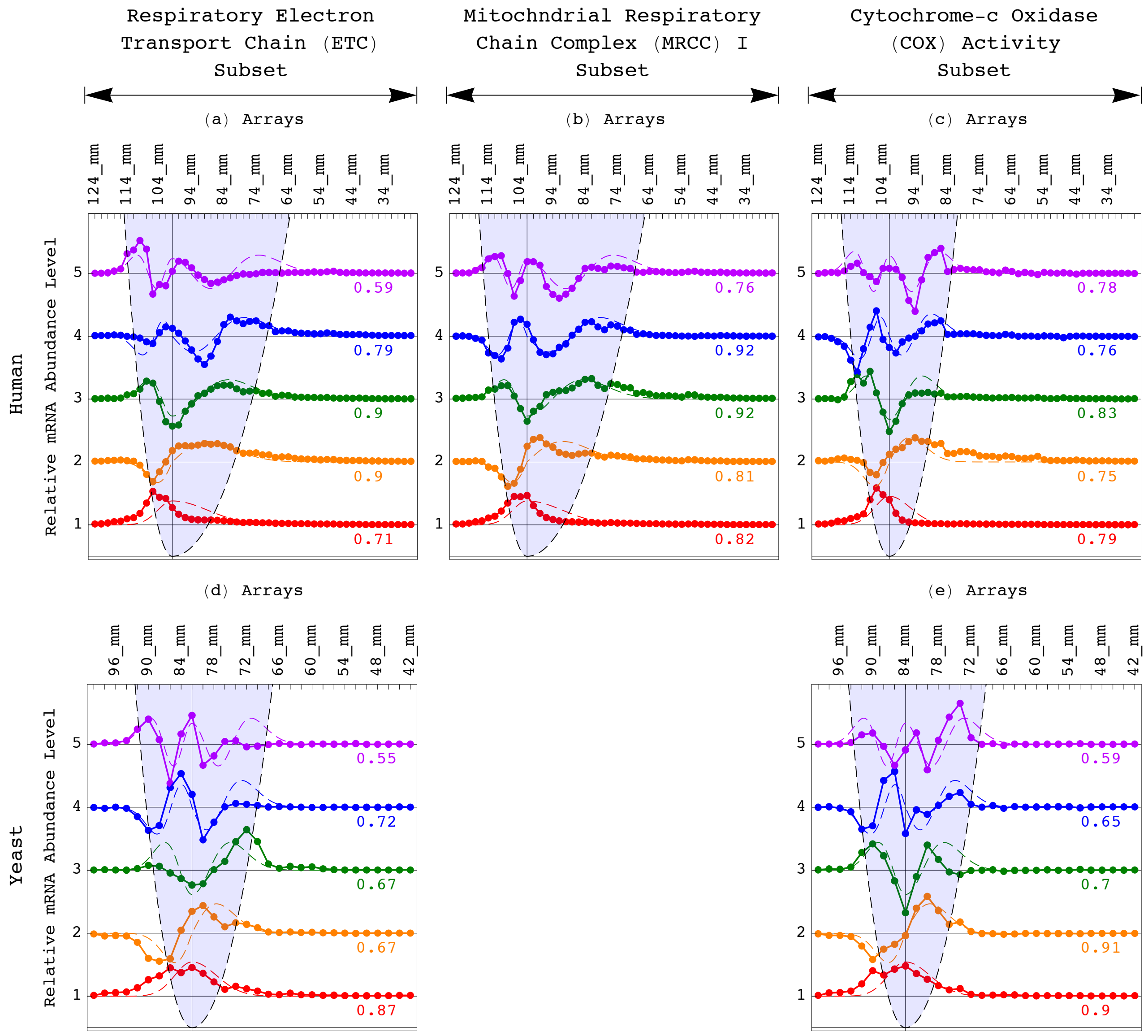


```
Export[path <> "Figures/Figure_S1.pdf", figS1, "PDF", ImageSize → 700, ImageResolution → resolution];
```

```
(* SVD of the Transcript Length Data of the Human Glucose Metabolism Subsets *)

(* Read the Transcript Length Data of the Human Glucose Metabolic Process Subset *)

stream = path <> "Data/Human_Transcript_Lengths.txt";
matrix = Import[stream, "Table"];
annotations = Position[matrix[[1]], "124_mm"][[1, 1]] - 1;
{genes, arrays} = Dimensions[matrix] - {1, annotations}
Clear[stream]

{4109, 50}

genenames = Take[matrix, {2, genes + 1}, {1, annotations}];
arraynames = Take[matrix, {1, 1}, {annotations + 1, arrays + annotations}];
annotationnames = Take[matrix, {1, 1}, {1, annotations}];
matrix = Take[matrix, {2, genes + 1}, {annotations + 1, arrays + annotations}];
matrix = ToExpression[matrix];
list = Take[genenames, All, {Position[annotationnames,
       "Glucose_Metabolic_Process_GO:0006006"][[1, 2]]}];
matrix = Take[Sort[Join[list, matrix, 2], OrderedQ[{#2, #1}] &], {1, Count[list, {"Y"}]},
    {2, arrays + 1}];
Dimensions[matrix]

{100, 50}

(* Calculate SVD *)

{eigenarrays, eigenabundances, eigengenes} =
  SingularValueDecomposition[matrix, Min[Dimensions[matrix]]];
eigengenes = Transpose[eigengenes];
eigenabundances = Diagonal[eigenabundances];
list = {1, 2, 3, 4, 5};
Do[{eigengenes[[list[[a]]]] = -eigengenes[[list[[a]]]]}, {a, 1, Dimensions[list][[1]]}]
{eigengenes[[3]], eigengenes[[4]], eigengenes[[5]]} =
  {eigengenes[[4]], eigengenes[[5]], eigengenes[[3]]};

(* Fit Eigenvectors with a Series of Asymmetric Hermite Functions *)

k1 = 0.065;
k2 = 0.065;
equi = 24;

Clear[g, h];
h[x_, n_, gamma_] := Exp[-gamma * x ^ 2 / 2] * HermiteH[n, Sqrt[gamma] * x] *
    Sqrt[Sqrt[gamma / Pi] / Factorial[n] / (2 ^ n)];
g[x_, n_] := If[x - equi < 0, h[x - equi, n - 1, k1] * (1 / k1) ^ 0.25,
    h[x - equi, n - 1, k2] * (1 / k2) ^ 0.25];
normalization = Table[
    Sqrt[Sum[g[x, n] ^ 2., {x, 0, arrays - 1}]],
    {n, 1, 5}];
correlation = Table[
    Sum[g[x, n] * eigengenes[[n, x + 1]] / normalization[[n]], {x, 0, arrays - 1}],
    {n, 1, 5}];
meancorrelation = Round[100. * Sqrt[Sum[0.2 * correlation[[n]] ^ 2, {n, 1, 5}]]] / 100.
correlation = Round[100. * correlation] / 100.;

0.75

(* Fit Differential Equation with an Asymmetric Parabola *)

Clear[f];
f[x_] := If[(x - equi) < 0, 0.5 * k1 * (x - equi) ^ 2, 0.5 * k2 * (x - equi) ^ 2];
```

```
(* Create Selected Eigenvectors Graph Display with Fitting Graphs *)

labelx = "(a) Arrays";
labely = "Relative mRNA Abundance Level";
framex = Table[{a - 1, Rotate[arraynames[[1, a]], Pi / 2]}, {a, 1, arrays}];
Do[If[Mod[a - 1, 5] ≠ 0, framex[[a, 2]] = Rotate["       ", Pi / 2]], {a, 1, Dimensions[framex][[1]]}]
framey = Table[{n - 0.5, n}, {n, 1, 5}];
points = Table[0, {n, 1, 5}];
lines = Table[0, {n, 1, 5}];
Do[{coordinates = Table[{a - 1, eigengenes[[n, a]] + n - 0.5}, {a, 1, arrays}],
  points[[n]] = Table[Point[coordinates[[a]]], {a, 1, arrays}],
  lines[[n]] = Line[coordinates]}, {n, 1, 5}]
points = Table[Graphics[{color[[Mod[n, 5] + 1]], PointSize[0.022], points[[n]]}], {n, 1, 5}];
lines = Table[Graphics[{Thickness[.005], color[[Mod[n, 5] + 1]], lines[[n]]}], {n, 1, 5}];
graphs = Table[Plot[g[x, n] / normalization[[n]] + n - 0.5, {x, 0, arrays - 1},
    PlotStyle → {color[[Mod[n, 5] + 1]], Dashing[{0.03, 0.02}]}, PlotRange → All], {n, 1, 5}];
texts = Table[Graphics[{color[[Mod[n, 5] + 1]], Text[correlation[[n]], {40, n - 0.75}, {-1, 0}]}],
   {n, 1, 5}];
inflection = Plot[f[x], {x, equi - Sqrt[5.45 * 2 / k1], equi + Sqrt[5.45 * 2 / k2]},
   PlotStyle → {RGBColor[0, 0, 0], Dashing[{0.03, 0.02}]},
   Filling → Top, FillingStyle → Opacity[0.1, RGBColor[0, 0, 1]]];

g44 = Show[{points, lines, graphs, texts, inflection},
   Frame → True,
   FrameLabel → {None, labely, labelx, None},
   FrameTicks → {None, framey, framex, None},
   GridLines → {{{equi, RGBColor[0, 0, 0]}},
     Join[{{0, RGBColor[0, 0, 0]}}, Table[{a - 0.5, RGBColor[0, 0, 0]}, {a, 1, 5}]]},
   PlotRange → {-0.05, 5.45}, AspectRatio → 1.05];

(* Read the Transcript Length Data of the Human Glycolysis Subset *)

stream = path <> "Data/Human_Transcript_Lengths.txt";
matrix = Import[stream, "Table"];
annotations = Position[matrix[[1]], "124_mm"][[1, 1]] - 1;
{genes, arrays} = Dimensions[matrix] - {1, annotations}
Clear[stream]

{4109, 50}

genenames = Take[matrix, {2, genes + 1}, {1, annotations}];
arraynames = Take[matrix, {1, 1}, {annotations + 1, arrays + annotations}];
annotationnames = Take[matrix, {1, 1}, {1, annotations}];
matrix = Take[matrix, {2, genes + 1}, {annotations + 1, arrays + annotations}];
matrix = ToExpression[matrix];
list = Take[genenames, All, {Position[annotationnames,
      "Glycolysis_GO:0006096"][[1, 2]]}];
matrix = Take[Sort[Join[list, matrix, 2], OrderedQ[{#2, #1}] &], {1, Count[list, {"Y"}]},
   {2, arrays + 1}];
Dimensions[matrix]

{29, 50}

(* Calculate SVD *)

{eigenarrays, eigenabundances, eigengenes} =
  SingularValueDecomposition[matrix, Min[Dimensions[matrix]]];
eigengenes = Transpose[eigengenes];
eigenabundances = Diagonal[eigenabundances];
list = {1, 2, 4, 5};
Do[{eigengenes[[list[[a]]]] = -eigengenes[[list[[a]]]]}, {a, 1, Dimensions[list][[1]]}]
{eigengenes[[4]], eigengenes[[5]]} = {eigengenes[[4]], eigengenes[[6]]};
```

```
(* Fit Eigenvectors with a Series of Asymmetric Hermite Functions *)

k1 = 0.195;
k2 = 0.195;
equi = 23;

Clear[g, h];
h[x_, n_, gamma_] := Exp[-gamma * x^2 / 2] * HermiteH[n, Sqrt[gamma] * x] *
   Sqrt[Sqrt[gamma / Pi] / Factorial[n] / (2^n)];
g[x_, n_] := If[x - equi < 0, h[x - equi, n - 1, k1] * (1 / k1)^0.25,
   h[x - equi, n - 1, k2] * (1 / k2)^0.25];
normalization = Table[
   Sqrt[Sum[g[x, n]^2., {x, 0, arrays - 1}]],
   {n, 1, 5}];
correlation = Table[
   Sum[g[x, n] * eigengenes[[n, x + 1]] / normalization[[n]], {x, 0, arrays - 1}],
   {n, 1, 5}];
meancorrelation = Round[100. * Sqrt[Sum[0.2 * correlation[[n]]^2, {n, 1, 5}]]] / 100.
correlation = Round[100. * correlation] / 100.;

0.65

(* Fit Differential Equation with an Asymmetric Parabola *)

Clear[f];
f[x_] := If[(x - equi) < 0, 0.5 * k1 * (x - equi)^2, 0.5 * k2 * (x - equi)^2];

(* Create Selected Eigenvectors Graph Display with Fitting Graphs *)

labelx = "(b) Arrays";
framex = Table[{a - 1, Rotate[arraynames[[1, a]], Pi / 2]}, {a, 1, arrays}];
Do[If[Mod[a - 1, 5] ≠ 0, framex[[a, 2]] = Rotate["       ", Pi / 2]], {a, 1, Dimensions[framex][[1]]}]
framey = Table[{n - 0.5, n}, {n, 1, 5}];
points = Table[0, {n, 1, 5}];
lines = Table[0, {n, 1, 5}];
Do[{coordinates = Table[{a - 1, eigengenes[[n, a]] + n - 0.5}, {a, 1, arrays}],
  points[[n]] = Table[Point[coordinates[[a]]], {a, 1, arrays}],
  lines[[n]] = Line[coordinates]}, {n, 1, 5}]
points = Table[Graphics[{color[[Mod[n, 5] + 1]], PointSize[0.022], points[[n]]}], {n, 1, 5}];
lines = Table[Graphics[{Thickness[.005], color[[Mod[n, 5] + 1]], lines[[n]]}], {n, 1, 5}];
graphs = Table[Plot[g[x, n] / normalization[[n]] + n - 0.5, {x, 0, arrays - 1},
    PlotStyle → {color[[Mod[n, 5] + 1]], Dashing[{0.03, 0.02}]}, PlotRange → All], {n, 1, 5}];
texts = Table[Graphics[{color[[Mod[n, 5] + 1]], Text[correlation[[n]], {40, n - 0.75}, {-1, 0}]}],
   {n, 1, 5}];
inflection = Plot[f[x], {x, equi - Sqrt[5.45 * 2 / k1], equi + Sqrt[5.45 * 2 / k2]},
   PlotStyle → {RGBColor[0, 0, 0], Dashing[{0.03, 0.02}]},
   Filling → Top, FillingStyle → Opacity[0.1, RGBColor[0, 0, 1]]];

g45 = Show[{points, lines, graphs, texts, inflection},
   Frame → True,
   FrameLabel → {None, None, labelx, None},
   FrameTicks → {None, framey, framex, None},
   GridLines → {{{equi, RGBColor[0, 0, 0]}},
     Join[{{0, RGBColor[0, 0, 0]}}, Table[{a - 0.5, RGBColor[0, 0, 0]}, {a, 1, 5}]]},
   PlotRange → {-0.05, 5.45}, AspectRatio → 1.05];
```

```
(* SVD of the Transcript Length Data of the Yeast Glucose Metabolism Subsets *)

(* Read the Transcript Length Data of the Yeast Glucose Metabolic Process Subset *)

stream = path <> "Data/Yeast_Transcript_Lengths.txt";
matrix = Import[stream, "Table"];
annotations = Position[matrix[[1]], "100_mm"][[1, 1]] - 1;
{genes, arrays} = Dimensions[matrix] - {1, annotations}
Clear[stream]

{3620, 30}

genenames = Take[matrix, {2, genes + 1}, {1, annotations}];
arraynames = Take[matrix, {1, 1}, {annotations + 1, arrays + annotations}];
annotationnames = Take[matrix, {1, 1}, {1, annotations}];
matrix = Take[matrix, {2, genes + 1}, {annotations + 1, arrays + annotations}];
matrix = ToExpression[matrix];
list = Take[genenames, All, {Position[annotationnames,
       "Glucose_Metabolic_Process_GO:0006006"][[1, 2]]}];
matrix = Take[Sort[Join[list, matrix, 2], OrderedQ[{#2, #1}] &], {1, Count[list, {"Y"}]},
    {2, arrays + 1}];
Dimensions[matrix]

{66, 30}

(* Calculate SVD *)

{eigenarrays, eigenabundances, eigengenes} =
  SingularValueDecomposition[matrix, Min[Dimensions[matrix]]];
eigengenes = Transpose[eigengenes];
eigenabundances = Diagonal[eigenabundances];
list = {1, 2, 4};
Do[{eigengenes[[list[[a]]]] = -eigengenes[[list[[a]]]]}, {a, 1, Dimensions[list][[1]]}]

(* Fit Eigenvectors with a Series of Asymmetric Hermite Functions *)

k1 = 0.2;
k2 = 0.2;
equi = 15;

Clear[g, h];
h[x_, n_, gamma_] := Exp[-gamma * x^2 / 2] * HermiteH[n, Sqrt[gamma] * x] *
    Sqrt[Sqrt[gamma / Pi] / Factorial[n] / (2^n)];
g[x_, n_] := If[x - equi < 0, h[x - equi, n - 1, k1] * (1 / k1)^0.25,
    h[x - equi, n - 1, k2] * (1 / k2)^0.25];
normalization = Table[
    Sqrt[Sum[g[x, n]^2., {x, 0, arrays - 1}]],
    {n, 1, 5}];
correlation = Table[
    Sum[g[x, n] * eigengenes[[n, x + 1]] / normalization[[n]], {x, 0, arrays - 1}],
    {n, 1, 5}];
meancorrelation = Round[100. * Sqrt[Sum[0.2 * correlation[[n]]^2, {n, 1, 5}]]] / 100.
correlation = Round[100. * correlation] / 100.;

0.81

(* Fit Differential Equation with an Asymmetric Parabola *)

Clear[f];
f[x_] := If[(x - equi) < 0, 0.5 * k1 * (x - equi)^2, 0.5 * k2 * (x - equi)^2];
```

```
(* Create Selected Eigenvectors Graph Display with Fitting Graphs *)

labelx = "(c) Arrays";
labely = "Relative mRNA Abundance Level";
framex = Table[{a - 1, Rotate[arraynames[[1, a]], Pi / 2]}, {a, 1, arrays}];
Do[If[Mod[a + 3, 3] ≠ 0, framex[[a, 2]] = Rotate["        ", Pi / 2]], {a, 1, Dimensions[framex][[1]]}]
framey = Table[{n - 0.5, n}, {n, 1, 5}];
points = Table[0, {n, 1, 5}];
lines = Table[0, {n, 1, 5}];
Do[{coordinates = Table[{a - 1, eigengenes[[n, a]] + n - 0.5}, {a, 1, arrays}],
  points[[n]] = Table[Point[coordinates[[a]]], {a, 1, arrays}],
  lines[[n]] = Line[coordinates]}, {n, 1, 5}]
points = Table[Graphics[{color[[Mod[n, 5] + 1]], PointSize[0.022], points[[n]]}], {n, 1, 5}];
lines = Table[Graphics[{Thickness[.005], color[[Mod[n, 5] + 1]], lines[[n]]}], {n, 1, 5}];
graphs = Table[Plot[g[x, n] / normalization[[n]] + n - 0.5, {x, 0, arrays - 1},
     PlotStyle → {color[[Mod[n, 5] + 1]], Dashing[{0.03, 0.02}]}, PlotRange → All], {n, 1, 5}];
texts = Table[Graphics[{color[[Mod[n, 5] + 1]], Text[correlation[[n]], {24, n - 0.75}, {-1, 0}]}],
    {n, 1, 5}];
inflection = Plot[f[x], {x, equi - Sqrt[5.45 * 2 / k1], equi + Sqrt[5.45 * 2 / k2]},
    PlotStyle → {RGBColor[0, 0, 0], Dashing[{0.03, 0.02}]},
    Filling → Top, FillingStyle → Opacity[0.1, RGBColor[0, 0, 1]]];

g46 = Show[{points, lines, graphs, texts, inflection},
    Frame → True,
    FrameLabel → {None, labely, labelx, None},
    FrameTicks → {None, framey, framex, None},
    GridLines → {{{equi, RGBColor[0, 0, 0]}},
      Join[{{0, RGBColor[0, 0, 0]}}, Table[{a - 0.5, RGBColor[0, 0, 0]}, {a, 1, 5}]]},
    PlotRange → {-0.05, 5.45}, AspectRatio → 1.05];

(* Read the Transcript Length Data of the Yeast Glycolysis Subset *)

stream = path <> "Data/Yeast_Transcript_Lengths.txt";
matrix = Import[stream, "Table"];
annotations = Position[matrix[[1]], "100_mm"][[1, 1]] - 1;
{genes, arrays} = Dimensions[matrix] - {1, annotations}
Clear[stream]

{3620, 30}

genenames = Take[matrix, {2, genes + 1}, {1, annotations}];
arraynames = Take[matrix, {1, 1}, {annotations + 1, arrays + annotations}];
annotationnames = Take[matrix, {1, 1}, {1, annotations}];
matrix = Take[matrix, {2, genes + 1}, {annotations + 1, arrays + annotations}];
matrix = ToExpression[matrix];
list = Take[genenames, All, {Position[annotationnames,
       "Glycolysis_GO:0006096"][[1, 2]]}];
matrix = Take[Sort[Join[list, matrix, 2], OrderedQ[{#2, #1}] &], {1, Count[list, {"Y"}]},
    {2, arrays + 1}];
Dimensions[matrix]

{23, 30}

(* Calculate SVD *)

{eigenarrays, eigenabundances, eigengenes} =
  SingularValueDecomposition[matrix, Min[Dimensions[matrix]]];
eigengenes = Transpose[eigengenes];
eigenabundances = Diagonal[eigenabundances];
list = {1, 5};
Do[{eigengenes[[list[[a]]]] = -eigengenes[[list[[a]]]]}, {a, 1, Dimensions[list][[1]]}]
{eigengenes[[4]], eigengenes[[5]]} =
  {(eigengenes[[4]] + eigengenes[[5]]) / Sqrt[2], (eigengenes[[4]] - eigengenes[[5]]) / Sqrt[2]};
```

```
(* Fit Eigenvectors with a Series of Asymmetric Hermite Functions *)

k1 = 0.4;
k2 = 0.4;
equi = 15;

Clear[g, h];
h[x_, n_, gamma_] := Exp[-gamma * x^2 / 2] * HermiteH[n, Sqrt[gamma] * x] *
   Sqrt[Sqrt[gamma / Pi] / Factorial[n] / (2^n)];
g[x_, n_] := If[x - equi < 0, h[x - equi, n - 1, k1] * (1 / k1)^0.25,
   h[x - equi, n - 1, k2] * (1 / k2)^0.25];
normalization = Table[
   Sqrt[Sum[g[x, n]^2., {x, 0, arrays - 1}]],
   {n, 1, 5}];
correlation = Table[
   Sum[g[x, n] * eigengenes[[n, x + 1]] / normalization[[n]], {x, 0, arrays - 1}],
   {n, 1, 5}];
meancorrelation = Round[100. * Sqrt[Sum[0.2 * correlation[[n]]^2, {n, 1, 5}]]] / 100.
correlation = Round[100. * correlation] / 100.;

0.67

(* Fit Differential Equation with an Asymmetric Parabola *)

Clear[f];
f[x_] := If[(x - equi) < 0, 0.5 * k1 * (x - equi)^2, 0.5 * k2 * (x - equi)^2];

(* Create Selected Eigenvectors Graph Display with Fitting Graphs *)

labelx = "(d) Arrays";
framex = Table[{a - 1, Rotate[arraynames[[1, a]], Pi / 2]}, {a, 1, arrays}];
Do[If[Mod[a + 3, 3] ≠ 0, framex[[a, 2]] = Rotate["       ", Pi / 2]], {a, 1, Dimensions[framex][[1]]}]
framey = Table[{n - 0.5, n}, {n, 1, 5}];
points = Table[0, {n, 1, 5}];
lines = Table[0, {n, 1, 5}];

Do[{coordinates = Table[{a - 1, eigengenes[[n, a]] + n - 0.5}, {a, 1, arrays}],
  points[[n]] = Table[Point[coordinates[[a]]], {a, 1, arrays}],
  lines[[n]] = Line[coordinates]}, {n, 1, 5}]
points = Table[Graphics[{color[[Mod[n, 5] + 1]], PointSize[0.022], points[[n]]}], {n, 1, 5}];
lines = Table[Graphics[{Thickness[.005], color[[Mod[n, 5] + 1]], lines[[n]]}], {n, 1, 5}];
graphs = Table[Plot[g[x, n] / normalization[[n]] + n - 0.5, {x, 0, arrays - 1},
    PlotStyle → {color[[Mod[n, 5] + 1]], Dashing[{0.03, 0.02}]}, PlotRange → All], {n, 1, 5}];
texts = Table[Graphics[{color[[Mod[n, 5] + 1]], Text[correlation[[n]], {24, n - 0.75}, {-1, 0}]}],
   {n, 1, 5}];
inflection = Plot[f[x], {x, equi - Sqrt[5.45 * 2 / k1], equi + Sqrt[5.45 * 2 / k2]},
   PlotStyle → {RGBColor[0, 0, 0], Dashing[{0.03, 0.02}]},
   Filling → Top, FillingStyle → Opacity[0.1, RGBColor[0, 0, 1]]];

g47 = Show[{points, lines, graphs, texts, inflection},
   Frame → True,
   FrameLabel → {None, None, labelx, None},
   FrameTicks → {None, framey, framex, None},
   GridLines → {{{equi, RGBColor[0, 0, 0]}},
     Join[{{0, RGBColor[0, 0, 0]}}, Table[{a - 0.5, RGBColor[0, 0, 0]}, {a, 1, 5}]]},
   PlotRange → {-0.05, 5.45}, AspectRatio → 1.05];
```

```
(* Display the SVD of the Human and Yeast Glucose Metabolism Subsets *)

g = GraphicsGrid[{{g44, g45}, {g46, g47}}, Spacings → {-40, 20}, ImageSize → 468];

figS2 = Show[{Graphics[{Rectangle[{0, 0}, {585, 667}, g]}, ImageSize → 468],
   Graphics[{RGBColor[0, 0, 0], Text[Style[
        ColumnForm[{"Glucose Metabolic Process", "Subset"}, Center],
        FontSize → 12], {170, 706}]}],
   Graphics[{RGBColor[0, 0, 0], Arrowheads[{-0.024, 0.024}], Arrow[{{50, 679}, {290, 679}}]}],
   Graphics[{RGBColor[0, 0, 0], Line[{{50, 673}, {50, 685}}]}],
   Graphics[{RGBColor[0, 0, 0], Line[{{290, 673}, {290, 685}}]}],
   Graphics[{RGBColor[0, 0, 0], Text[Style[
        ColumnForm[{"Glycolysis", "Subset"}, Center],
        FontSize → 12], {434, 706}]}],
   Graphics[{RGBColor[0, 0, 0], Arrowheads[{-0.024, 0.024}], Arrow[{{314, 679}, {554, 679}}]}],
   Graphics[{RGBColor[0, 0, 0], Line[{{314, 673}, {314, 685}}]}],
   Graphics[{RGBColor[0, 0, 0], Line[{{554, 673}, {554, 685}}]}],
   Graphics[{RGBColor[0, 0, 0], Text[Style[Rotate["Human", Pi / 2], FontSize → 12], {0, 467}]}],
   Graphics[{RGBColor[0, 0, 0], Text[Style[Rotate["Yeast", Pi / 2], FontSize → 12], {0, 127}]}]
  }]
```

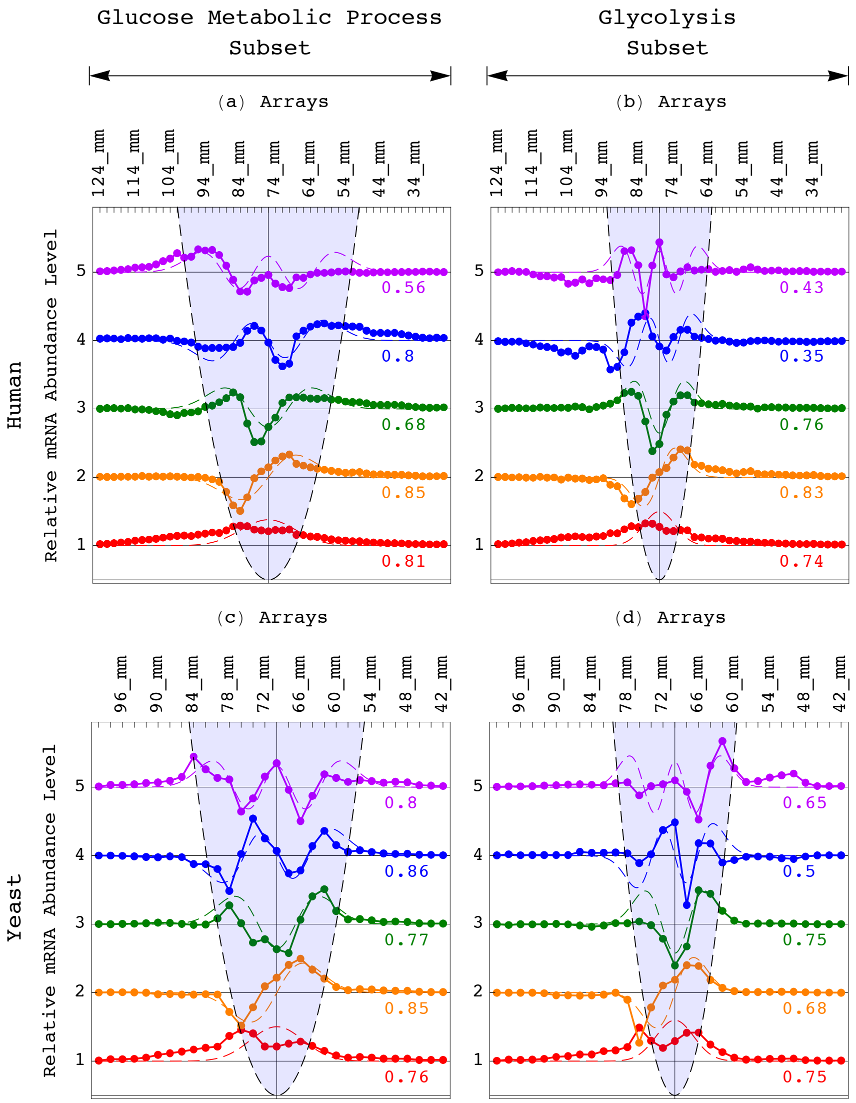


```
Export[path <> "Figures/Figure_S2.pdf", figS2, "PDF", ImageSize → 468, ImageResolution → resolution];
```

```
(* SVD of the Transcript Length Data of the Human Brain Activity Subsets *)

(* Read the Transcript Length Data of the Human Neuron Projection Subset *)

stream = path <> "Data/Human_Transcript_Lengths.txt";
matrix = Import[stream, "Table"];
annotations = Position[matrix[[1]], "124_mm"][[1, 1]] - 1;
{genes, arrays} = Dimensions[matrix] - {1, annotations}
Clear[stream]

{4109, 50}

genenames = Take[matrix, {2, genes + 1}, {1, annotations}];
arraynames = Take[matrix, {1, 1}, {annotations + 1, arrays + annotations}];
annotationnames = Take[matrix, {1, 1}, {1, annotations}];
matrix = Take[matrix, {2, genes + 1}, {annotations + 1, arrays + annotations}];
matrix = ToExpression[matrix];
list = Take[genenames, All, {Position[annotationnames,
       "Neuron_Projection_GO:0043005"][[1, 2]]}];
matrix = Take[Sort[Join[list, matrix, 2], OrderedQ[{#2, #1}] &], {1, Count[list, {"Y"}]},
    {2, arrays + 1}];
Dimensions[matrix]

{259, 50}

(* Calculate SVD *)

{eigenarrays, eigenabundances, eigengenes} =
  SingularValueDecomposition[matrix, Min[Dimensions[matrix]]];
eigengenes = Transpose[eigengenes];
eigenabundances = Diagonal[eigenabundances];
list = {1, 3, 4, 5};
Do[{eigengenes[[list[[a]]]] = -eigengenes[[list[[a]]]]}, {a, 1, Dimensions[list][[1]]}]

(* Fit Eigenvectors with a Series of Asymmetric Hermite Functions *)

k1 = 0.0325;
k2 = 0.0325;
equi = 23;

Clear[g, h];
h[x_, n_, gamma_] := Exp[-gamma * x ^ 2 / 2] * HermiteH[n, Sqrt[gamma] * x] *
    Sqrt[Sqrt[gamma / Pi] / Factorial[n] / (2 ^ n)];
g[x_, n_] := If[x - equi < 0, h[x - equi, n - 1, k1] * (1 / k1) ^ 0.25,
    h[x - equi, n - 1, k2] * (1 / k2) ^ 0.25];
normalization = Table[
    Sqrt[Sum[g[x, n] ^ 2., {x, 0, arrays - 1}]],
    {n, 1, 5}];
correlation = Table[
    Sum[g[x, n] * eigengenes[[n, x + 1]] / normalization[[n]], {x, 0, arrays - 1}],
    {n, 1, 5}];
meancorrelation = Round[100. * Sqrt[Sum[0.2 * correlation[[n]] ^ 2, {n, 1, 5}]]] / 100.
correlation = Round[100. * correlation] / 100.;

0.86

(* Fit Differential Equation with an Asymmetric Parabola *)

Clear[f];
f[x_] := If[(x - equi) < 0, 0.5 * k1 * (x - equi) ^ 2, 0.5 * k2 * (x - equi) ^ 2];
```

```
(* Create Selected Eigenvectors Graph Display with Fitting Graphs *)

labelx = "(a) Arrays";
labely = "Relative mRNA Abundance Level";
framex = Table[{a - 1, Rotate[arraynames[[1, a]], Pi / 2]}, {a, 1, arrays}];
Do[If[Mod[a - 1, 5] ≠ 0, framex[[a, 2]] = Rotate["       ", Pi / 2]], {a, 1, Dimensions[framex][[1]]}]
framey = Table[{n - 0.5, n}, {n, 1, 5}];
points = Table[0, {n, 1, 5}];
lines = Table[0, {n, 1, 5}];
Do[{coordinates = Table[{a - 1, eigengenes[[n, a]] + n - 0.5}, {a, 1, arrays}],
  points[[n]] = Table[Point[coordinates[[a]]], {a, 1, arrays}],
  lines[[n]] = Line[coordinates]}, {n, 1, 5}]
points = Table[Graphics[{color[[Mod[n, 5] + 1]], PointSize[0.022], points[[n]]}], {n, 1, 5}];
lines = Table[Graphics[{Thickness[.005], color[[Mod[n, 5] + 1]], lines[[n]]}], {n, 1, 5}];
graphs = Table[Plot[g[x, n] / normalization[[n]] + n - 0.5, {x, 0, arrays - 1},
     PlotStyle → {color[[Mod[n, 5] + 1]], Dashing[{0.03, 0.02}]}, PlotRange → All], {n, 1, 5}];
texts = Table[Graphics[{color[[Mod[n, 5] + 1]], Text[correlation[[n]], {40, n - 0.75}, {-1, 0}]}],
    {n, 1, 5}];
inflection = Plot[f[x], {x, equi - Sqrt[5.45 * 2 / k1], equi + Sqrt[5.45 * 2 / k2]},
    PlotStyle → {RGBColor[0, 0, 0], Dashing[{0.03, 0.02}]},
    Filling → Top, FillingStyle → Opacity[0.1, RGBColor[0, 0, 1]]];

g48 = Show[{points, lines, graphs, texts, inflection},
    Frame → True,
    FrameLabel → {None, labely, labelx, None},
    FrameTicks → {None, framey, framex, None},
    GridLines → {{{equi, RGBColor[0, 0, 0]}},
      Join[{{0, RGBColor[0, 0, 0]}}, Table[{a - 0.5, RGBColor[0, 0, 0]}, {a, 1, 5}]]},
    PlotRange → {-0.05, 5.45}, AspectRatio → 1.05];

(* Read the Transcript Length Data of the Human Synaptic Transmission Subset *)

stream = path <> "Data/Human_Transcript_Lengths.txt";
matrix = Import[stream, "Table"];
annotations = Position[matrix[[1]], "124_mm"][[1, 1]] - 1;
{genes, arrays} = Dimensions[matrix] - {1, annotations}
Clear[stream]

{4109, 50}

genenames = Take[matrix, {2, genes + 1}, {1, annotations}];
arraynames = Take[matrix, {1, 1}, {annotations + 1, arrays + annotations}];
annotationnames = Take[matrix, {1, 1}, {1, annotations}];
matrix = Take[matrix, {2, genes + 1}, {annotations + 1, arrays + annotations}];
matrix = ToExpression[matrix];
list = Take[genenames, All, {Position[annotationnames,
      "Synaptic_Transmission_GO:0007268"][[1, 2]]}];
matrix = Take[Sort[Join[list, matrix, 2], OrderedQ[{#2, #1}] &], {1, Count[list, {"Y"}]},
    {2, arrays + 1}];
Dimensions[matrix]

{238, 50}

(* Calculate SVD *)

{eigenarrays, eigenabundances, eigengenes} =
  SingularValueDecomposition[matrix, Min[Dimensions[matrix]]];
eigengenes = Transpose[eigengenes];
eigenabundances = Diagonal[eigenabundances];
list = {1, 3, 4, 5};
Do[{eigengenes[[list[[a]]]] = -eigengenes[[list[[a]]]]}, {a, 1, Dimensions[list][[1]]}]
```

```
(* Fit Eigenvectors with a Series of Asymmetric Hermite Functions *)

k1 = 0.0325;
k2 = 0.0325;
equi = 22;

Clear[g, h];
h[x_, n_, gamma_] := Exp[-gamma * x^2 / 2] * HermiteH[n, Sqrt[gamma] * x] *
   Sqrt[Sqrt[gamma / Pi] / Factorial[n] / (2^n)];
g[x_, n_] := If[x - equi < 0, h[x - equi, n - 1, k1] * (1 / k1)^0.25,
   h[x - equi, n - 1, k2] * (1 / k2)^0.25];
normalization = Table[
   Sqrt[Sum[g[x, n]^2., {x, 0, arrays - 1}]],
   {n, 1, 5}];
correlation = Table[
   Sum[g[x, n] * eigengenes[[n, x + 1]] / normalization[[n]], {x, 0, arrays - 1}],
   {n, 1, 5}];
meancorrelation = Round[100. * Sqrt[Sum[0.2 * correlation[[n]]^2, {n, 1, 5}]]] / 100.
correlation = Round[100. * correlation] / 100.;

0.79

(* Fit Differential Equation with an Asymmetric Parabola *)

Clear[f];
f[x_] := If[(x - equi) < 0, 0.5 * k1 * (x - equi)^2, 0.5 * k2 * (x - equi)^2];

(* Create Selected Eigenvectors Graph Display with Fitting Graphs *)

labelx = "(b) Arrays";
framex = Table[{a - 1, Rotate[arraynames[[1, a]], Pi / 2]}, {a, 1, arrays}];
Do[If[Mod[a - 1, 5] ≠ 0, framex[[a, 2]] = Rotate["       ", Pi / 2]], {a, 1, Dimensions[framex][[1]]}]
framey = Table[{n - 0.5, n}, {n, 1, 5}];
points = Table[0, {n, 1, 5}];
lines = Table[0, {n, 1, 5}];

Do[{coordinates = Table[{a - 1, eigengenes[[n, a]] + n - 0.5}, {a, 1, arrays}],
  points[[n]] = Table[Point[coordinates[[a]]], {a, 1, arrays}],
  lines[[n]] = Line[coordinates]}, {n, 1, 5}]
points = Table[Graphics[{color[[Mod[n, 5] + 1]], PointSize[0.022], points[[n]]}], {n, 1, 5}];
lines = Table[Graphics[{Thickness[.005], color[[Mod[n, 5] + 1]], lines[[n]]}], {n, 1, 5}];
graphs = Table[Plot[g[x, n] / normalization[[n]] + n - 0.5, {x, 0, arrays - 1},
    PlotStyle → {color[[Mod[n, 5] + 1]], Dashing[{0.03, 0.02}]}, PlotRange → All], {n, 1, 5}];
texts = Table[Graphics[{color[[Mod[n, 5] + 1]], Text[correlation[[n]], {40, n - 0.75}, {-1, 0}]}],
   {n, 1, 5}];
inflection = Plot[f[x], {x, equi - Sqrt[5.45 * 2 / k1], equi + Sqrt[5.45 * 2 / k2]},
   PlotStyle → {RGBColor[0, 0, 0], Dashing[{0.03, 0.02}]},
   Filling → Top, FillingStyle → Opacity[0.1, RGBColor[0, 0, 1]]];

g49 = Show[{points, lines, graphs, texts, inflection},
   Frame → True,
   FrameLabel → {None, None, labelx, None},
   FrameTicks → {None, framey, framex, None},
   GridLines → {{{equi, RGBColor[0, 0, 0]}},
     Join[{{0, RGBColor[0, 0, 0]}}, Table[{a - 0.5, RGBColor[0, 0, 0]}, {a, 1, 5}]]},
   PlotRange → {-0.05, 5.45}, AspectRatio → 1.05];
```

```
(* Display the SVD of the Human Brain Activity Subsets *)
```

```
g = GraphicsGrid[{{g48, g49}}, Spacings → {-40, 20}, ImageSize → 468];

figS3 = Show[{Graphics[{Rectangle[{0, 0}, {585, 351}, g]}, ImageSize → 468],
   Graphics[{RGBColor[0, 0, 0], Text[Style[
        ColumnForm[{"Neuron Projection", "Subset"}, Center],
        FontSize → 12], {170, 374}]}],
   Graphics[{RGBColor[0, 0, 0], Arrowheads[{-0.024, 0.024}], Arrow[{{50, 348}, {290, 348}}]}],
   Graphics[{RGBColor[0, 0, 0], Line[{{50, 342}, {50, 354}}]}],
   Graphics[{RGBColor[0, 0, 0], Line[{{290, 342}, {290, 354}}]}],
   Graphics[{RGBColor[0, 0, 0], Text[Style[
        ColumnForm[{"Synaptic Transmission", "Subset"}, Center],
        FontSize → 12], {434, 374}]}],
   Graphics[{RGBColor[0, 0, 0], Arrowheads[{-0.024, 0.024}], Arrow[{{314, 348}, {554, 348}}]}],
   Graphics[{RGBColor[0, 0, 0], Line[{{314, 342}, {314, 354}}]}],
   Graphics[{RGBColor[0, 0, 0], Line[{{554, 342}, {554, 354}}]}],
   Graphics[{RGBColor[0, 0, 0], Text[Style[Rotate["Human", Pi / 2], FontSize → 12], {0, 140}]}]
  }]
```

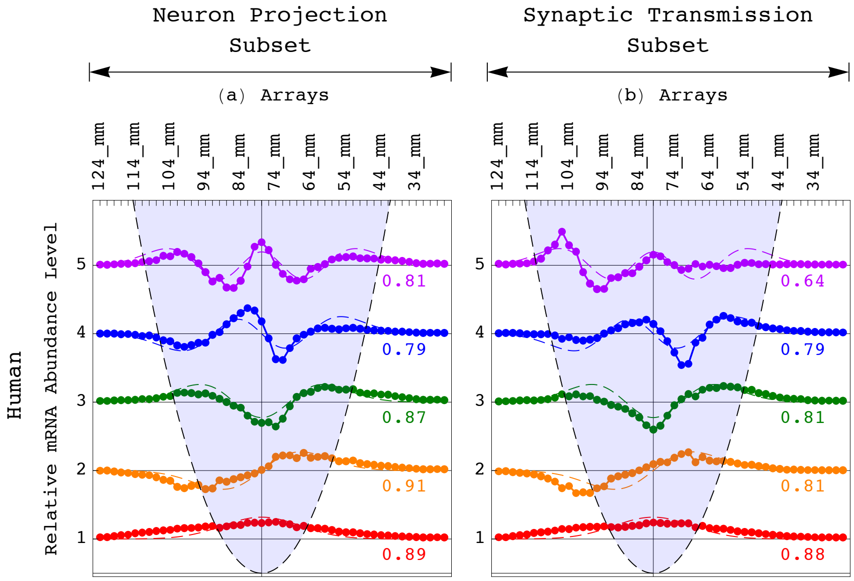


```
Export[path <> "Figures/Figure_S3.pdf", figS3, "PDF", ImageSize → 468, ImageResolution → resolution];
```

```
(* SVD of the Transcript Length Data of the Yeast DNA Damage Response Subsets *)

(* Read the Data of the Yeast Transcripts Overexpressed in Response to DNA Damage *)

stream = path <> "Data/Yeast_Transcript_Lengths.txt";
matrix = Import[stream, "Table"];
annotations = Position[matrix[[1]], "100_mm"][[1, 1]] - 1;
{genes, arrays} = Dimensions[matrix] - {1, annotations}
Clear[stream]

{3620, 30}

genenames = Take[matrix, {2, genes + 1}, {1, annotations}];
arraynames = Take[matrix, {1, 1}, {annotations + 1, arrays + annotations}];
annotationnames = Take[matrix, {1, 1}, {1, annotations}];
matrix = Take[matrix, {2, genes + 1}, {annotations + 1, arrays + annotations}];
matrix = ToExpression[matrix];
list = Take[genenames, All, {Position[annotationnames,
      "Jelinsky_et_al_DNA_Damage_Up"][[1, 2]]}];
matrix = Take[Sort[Join[list, matrix, 2], OrderedQ[{#2, #1}] &], {1, Count[list, {"Y"}]},
   {2, arrays + 1}];
Dimensions[matrix]

{194, 30}

(* Calculate SVD *)

{eigenarrays, eigenabundances, eigengenes} =
  SingularValueDecomposition[matrix, Min[Dimensions[matrix]]];
eigengenes = Transpose[eigengenes];
eigenabundances = Diagonal[eigenabundances];
list = {1, 3, 4, 5};
Do[{eigengenes[[list[[a]]]] = -eigengenes[[list[[a]]]]}, {a, 1, Dimensions[list][[1]]}]

(* Fit Eigenvectors with a Series of Asymmetric Hermite Functions *)

k1 = 0.2;
k2 = 0.1;
equi = 13;

Clear[g, h];
h[x_, n_, gamma_] := Exp[-gamma * x ^ 2 / 2] * HermiteH[n, Sqrt[gamma] * x] *
   Sqrt[Sqrt[gamma / Pi] / Factorial[n] / (2 ^ n)];
g[x_, n_] := If[x - equi < 0, h[x - equi, n - 1, k1] * (1 / k1) ^ 0.25,
   h[x - equi, n - 1, k2] * (1 / k2) ^ 0.25];
normalization = Table[
   Sqrt[Sum[g[x, n] ^ 2., {x, 0, arrays - 1}]],
   {n, 1, 5}];
correlation = Table[
   Sum[g[x, n] * eigengenes[[n, x + 1]] / normalization[[n]], {x, 0, arrays - 1}],
   {n, 1, 5}];
meancorrelation = Round[100. * Sqrt[Sum[0.2 * correlation[[n]] ^ 2, {n, 1, 5}]]] / 100.
correlation = Round[100. * correlation] / 100.;

0.81

(* Fit Differential Equation with an Asymmetric Parabola *)

Clear[f];
f[x_] := If[(x - equi) < 0, 0.5 * k1 * (x - equi) ^ 2, 0.5 * k2 * (x - equi) ^ 2];
```

```
(* Create Selected Eigenvectors Graph Display with Fitting Graphs *)

labelx = "(a) Arrays";
labely = "Relative mRNA Abundance Level";
framex = Table[{a - 1, Rotate[arraynames[[1, a]], Pi / 2]}, {a, 1, arrays}];
Do[If[Mod[a + 3, 3] ≠ 0, framex[[a, 2]] = Rotate["       ", Pi / 2]], {a, 1, Dimensions[framex][[1]]}]
framey = Table[{n - 0.5, n}, {n, 1, 5}];
points = Table[0, {n, 1, 5}];
lines = Table[0, {n, 1, 5}];
Do[{coordinates = Table[{a - 1, eigengenes[[n, a]] + n - 0.5}, {a, 1, arrays}],
  points[[n]] = Table[Point[coordinates[[a]]], {a, 1, arrays}],
  lines[[n]] = Line[coordinates]}, {n, 1, 5}]
points = Table[Graphics[{color[[Mod[n, 5] + 1]], PointSize[0.022], points[[n]]}], {n, 1, 5}];
lines = Table[Graphics[{Thickness[.005], color[[Mod[n, 5] + 1]], lines[[n]]}], {n, 1, 5}];
graphs = Table[Plot[g[x, n] / normalization[[n]] + n - 0.5, {x, 0, arrays - 1},
    PlotStyle → {color[[Mod[n, 5] + 1]], Dashing[{0.03, 0.02}]}, PlotRange → All], {n, 1, 5}];
texts = Table[Graphics[{color[[Mod[n, 5] + 1]], Text[correlation[[n]], {24, n - 0.75}, {-1, 0}]}],
   {n, 1, 5}];
inflection = Plot[f[x], {x, equi - Sqrt[5.45 * 2 / k1], equi + Sqrt[5.45 * 2 / k2]},
   PlotStyle → {RGBColor[0, 0, 0], Dashing[{0.03, 0.02}]},
   Filling → Top, FillingStyle → Opacity[0.1, RGBColor[0, 0, 1]]];

g50 = Show[{points, lines, graphs, texts, inflection},
   Frame → True,
   FrameLabel → {None, labely, labelx, None},
   FrameTicks → {None, framey, framex, None},
   GridLines → {{{equi, RGBColor[0, 0, 0]}},
     Join[{{0, RGBColor[0, 0, 0]}}, Table[{a - 0.5, RGBColor[0, 0, 0]}, {a, 1, 5}]]},
   PlotRange → {-0.05, 5.45}, AspectRatio → 1.05];

(* Read the Data of the Yeast Transcripts Underexpressed in Response to DNA Damage *)

stream = path <> "Data/Yeast_Transcript_Lengths.txt";
matrix = Import[stream, "Table"];
annotations = Position[matrix[[1]], "100_mm"][[1, 1]] - 1;
{genes, arrays} = Dimensions[matrix] - {1, annotations}
Clear[stream]

{3620, 30}

genenames = Take[matrix, {2, genes + 1}, {1, annotations}];
arraynames = Take[matrix, {1, 1}, {annotations + 1, arrays + annotations}];
annotationnames = Take[matrix, {1, 1}, {1, annotations}];
matrix = Take[matrix, {2, genes + 1}, {annotations + 1, arrays + annotations}];
matrix = ToExpression[matrix];
list = Take[genenames, All, {Position[annotationnames,
      "Jelinsky_et_al_DNA_Damage_Down"][[1, 2]]}];
matrix = Take[Sort[Join[list, matrix, 2], OrderedQ[{#2, #1}] &], {1, Count[list, {"Y"}]},
   {2, arrays + 1}];
Dimensions[matrix]

{52, 30}

(* Calculate SVD *)

{eigenarrays, eigenabundances, eigengenes} =
  SingularValueDecomposition[matrix, Min[Dimensions[matrix]]];
eigengenes = Transpose[eigengenes];
eigenabundances = Diagonal[eigenabundances];
list = {1, 2, 3};
Do[{eigengenes[[list[[a]]]] = -eigengenes[[list[[a]]]]}, {a, 1, Dimensions[list][[1]]}]
```

```
(* Fit Eigenvectors with a Series of Asymmetric Hermite Functions *)

k1 = 0.2;
k2 = 0.1;
equi = 10;

Clear[g, h];
h[x_, n_, gamma_] := Exp[-gamma * x^2 / 2] * HermiteH[n, Sqrt[gamma] * x] *
   Sqrt[Sqrt[gamma / Pi] / Factorial[n] / (2^n)];
g[x_, n_] := If[x - equi < 0, h[x - equi, n - 1, k1] * (1 / k1) ^0.25,
   h[x - equi, n - 1, k2] * (1 / k2) ^0.25];
normalization = Table[
   Sqrt[Sum[g[x, n] ^2., {x, 0, arrays - 1}]],
   {n, 1, 5}];
correlation = Table[
   Sum[g[x, n] * eigengenes[[n, x + 1]] / normalization[[n]], {x, 0, arrays - 1}],
   {n, 1, 5}];
meancorrelation = Round[100. * Sqrt[Sum[0.2 * correlation[[n]] ^2, {n, 1, 5}]]] / 100.
correlation = Round[100. * correlation] / 100.;

0.7

(* Fit Differential Equation with an Asymmetric Parabola *)

Clear[f];
f[x_] := If[(x - equi) < 0, 0.5 * k1 * (x - equi) ^2, 0.5 * k2 * (x - equi) ^2];

(* Create Selected Eigenvectors Graph Display with Fitting Graphs *)

labelx = "(b) Arrays";
framex = Table[{a - 1, Rotate[arraynames[[1, a]], Pi / 2]}, {a, 1, arrays}];
Do[If[Mod[a + 3, 3] ≠ 0, framex[[a, 2]] = Rotate["       ", Pi / 2]], {a, 1, Dimensions[framex][[1]]}]
framey = Table[{n - 0.5, n}, {n, 1, 5}];
points = Table[0, {n, 1, 5}];
lines = Table[0, {n, 1, 5}];

Do[{coordinates = Table[{a - 1, eigengenes[[n, a]] + n - 0.5}, {a, 1, arrays}],
  points[[n]] = Table[Point[coordinates[[a]]], {a, 1, arrays}],
  lines[[n]] = Line[coordinates]}, {n, 1, 5}]
points = Table[Graphics[{color[[Mod[n, 5] + 1]], PointSize[0.022], points[[n]]}], {n, 1, 5}];
lines = Table[Graphics[{Thickness[.005], color[[Mod[n, 5] + 1]], lines[[n]]}], {n, 1, 5}];
graphs = Table[Plot[g[x, n] / normalization[[n]] + n - 0.5, {x, 0, arrays - 1},
    PlotStyle → {color[[Mod[n, 5] + 1]], Dashing[{0.03, 0.02}]}, PlotRange → All], {n, 1, 5}];
texts = Table[Graphics[{color[[Mod[n, 5] + 1]], Text[correlation[[n]], {24, n - 0.75}, {-1, 0}]}],
   {n, 1, 5}];
inflection = Plot[f[x], {x, equi - Sqrt[5.45 * 2 / k1], equi + Sqrt[5.45 * 2 / k2]},
   PlotStyle → {RGBColor[0, 0, 0], Dashing[{0.03, 0.02}]},
   Filling → Top, FillingStyle → Opacity[0.1, RGBColor[0, 0, 1]]];

g51 = Show[{points, lines, graphs, texts, inflection},
   Frame → True,
   FrameLabel → {None, None, labelx, None},
   FrameTicks → {None, framey, framex, None},
   GridLines → {{{equi, RGBColor[0, 0, 0]}},
     Join[{{0, RGBColor[0, 0, 0]}}, Table[{a - 0.5, RGBColor[0, 0, 0]}, {a, 1, 5}]]},
   PlotRange → {-0.05, 5.45}, AspectRatio → 1.05];
```

```
(* Display the SVD of the Yeast DNA Damage Response Subsets *)

g = GraphicsGrid[{{g50, g51}}, Spacings → {-40, 20}, ImageSize → 468];

figS4 = Show[{Graphics[{Rectangle[{0, 0}, {585, 351}, g]}, ImageSize → 468],
   Graphics[{RGBColor[0, 0, 0], Text[Style[
       ColumnForm[{"DNA Damage", "Overexpression Subset"}, Center],
       FontSize → 12], {170, 374}]}],
   Graphics[{RGBColor[0, 0, 0], Arrowheads[{-0.024, 0.024}], Arrow[{{50, 348}, {290, 348}}]}],
   Graphics[{RGBColor[0, 0, 0], Line[{{50, 342}, {50, 354}}]}],
   Graphics[{RGBColor[0, 0, 0], Line[{{290, 342}, {290, 354}}]}],
   Graphics[{RGBColor[0, 0, 0], Text[Style[
       ColumnForm[{"DNA Damage", "Underexpression Subset"}, Center],
       FontSize → 12], {434, 374}]}],
   Graphics[{RGBColor[0, 0, 0], Arrowheads[{-0.024, 0.024}], Arrow[{{314, 348}, {554, 348}}]}],
   Graphics[{RGBColor[0, 0, 0], Line[{{314, 342}, {314, 354}}]}],
   Graphics[{RGBColor[0, 0, 0], Line[{{554, 342}, {554, 354}}]}],
   Graphics[{RGBColor[0, 0, 0], Text[Style[Rotate["Yeast", Pi / 2], FontSize → 12], {0, 140}]}]
  }]
```

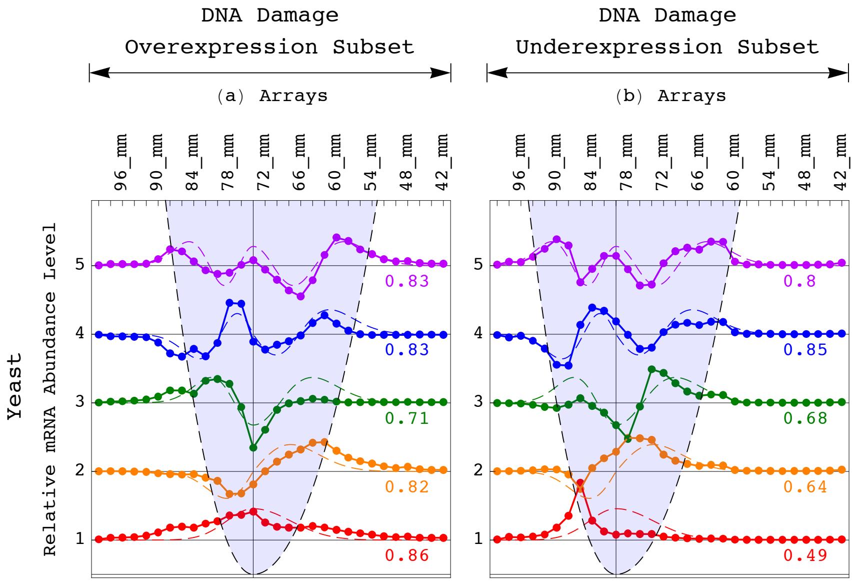


```
Export[path <> "Figures/Figure_S4.pdf", figS4, "PDF", ImageSize → 468, ImageResolution → resolution];
```
